# Supplementary material for: Safety, Efficacy, and Immunogenicity of Varying Types of COVID-19 Vaccines in Children Younger Than 18 Years: An Update of Systematic Review and Meta-Analysis
Source: Vaccines (Basel). 2022 Dec 30;11(1):87. doi: 10.3390/vaccines11010087 (PMC9864967; doi:10.3390/vaccines11010087)
Supplement: Supplementary file 1 [file vaccines-11-00087-s001.zip › vaccines-2100003-supplementary.pdf]

Figure S1. Funnel plot for the total adverse reactions.

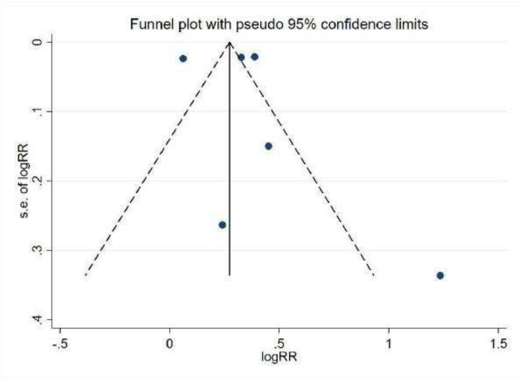

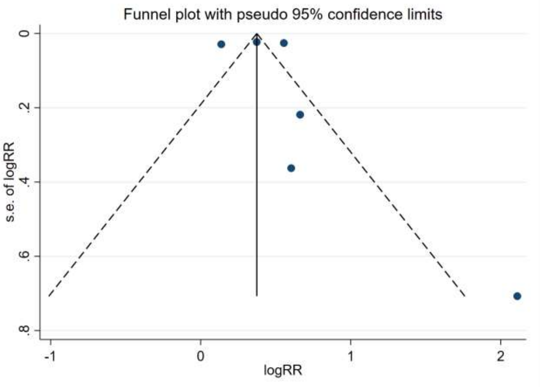

Egger test：$P = 0.766$

(**A**). Total adverse reactions after dose 1

Egger test：$P = 0.734$

(**B**). Total adverse reactions after dose 2

Figure S2. Funnel plot for the systemic and local adverse reactions.

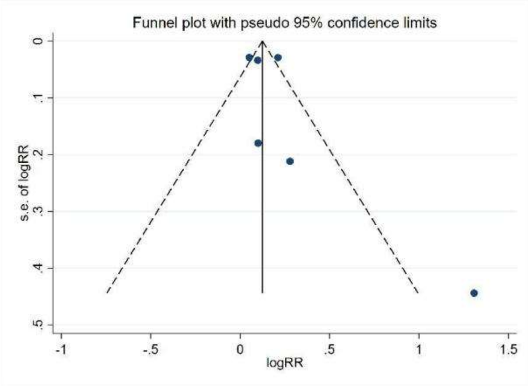

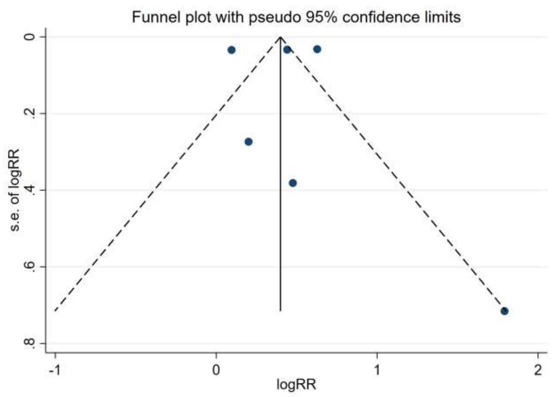

Egger test：$P = 0.439$

(**A**). Systemic adverse reactions after dose 1

Egger test：$P = 0.926$

(**B**). Systemic adverse reactions after dose 2

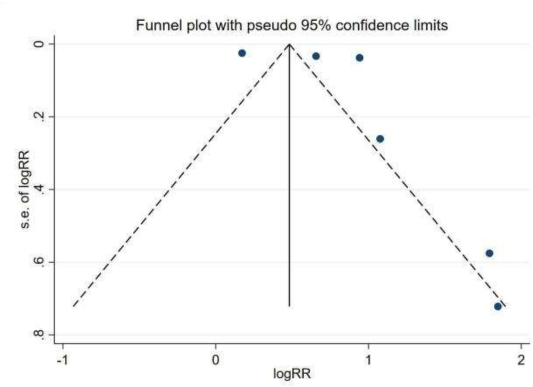

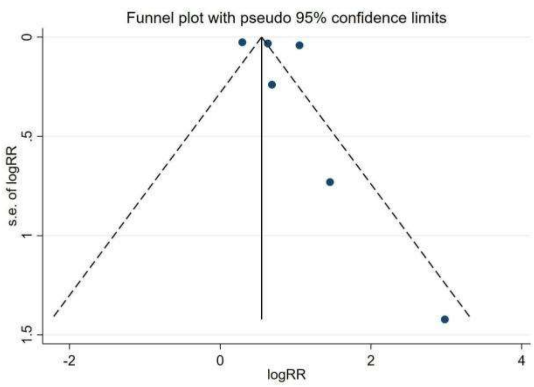

Egger test：$P = 0.460$

(**C**). Local adverse reactions after dose 1

Egger test：$P = 0.539$

(**D**). Local adverse reactions after dose 2

Figure S3. Funnel plot for the neutralizing antibody 28 days after dose 2.

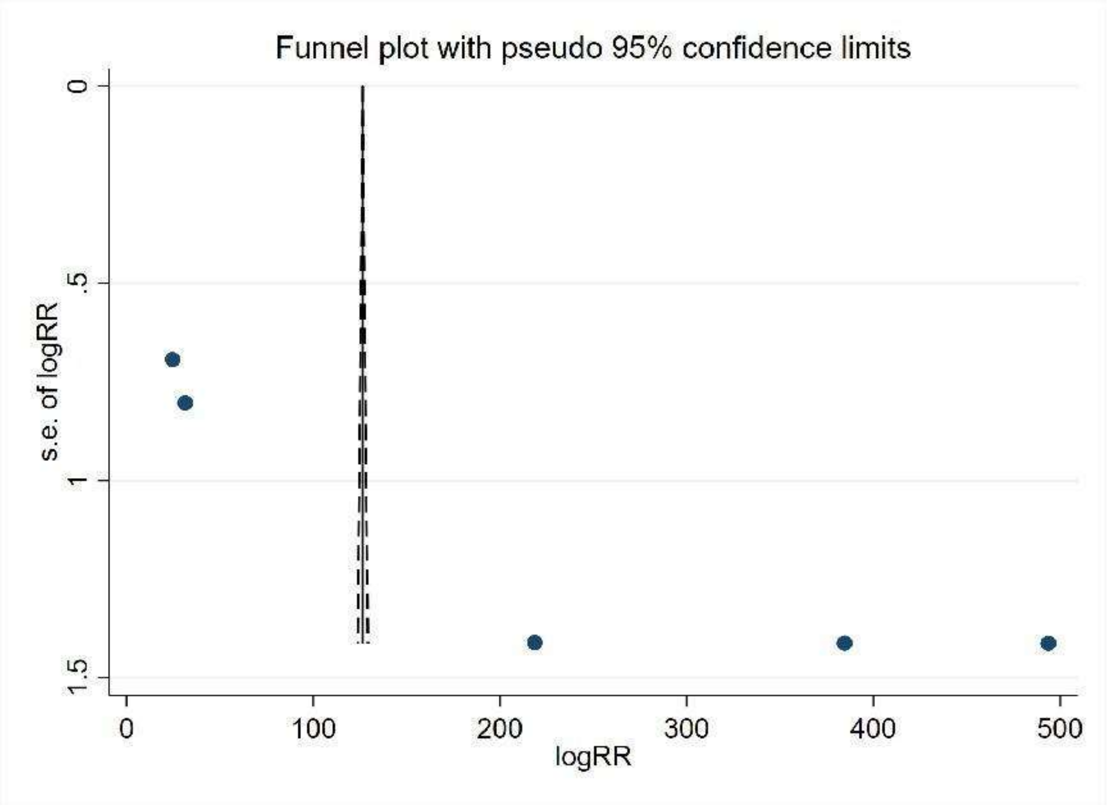

Egger test：$P = 0.026$

Figure S4. Adverse reactions among vaccination group versus control group: A) Total adverse reactions after dose 1; B) Total adverse reactions after dose 2; C) Systemic adverse reactions after dose 1; D) Systemic adverse reactions after dose 2; E) Local adverse reactions after dose 1; F) Local adverse reactions after dose 2:

A) Total adverse reactions after dose 1

Study or Subgroup	Experimental Events	Experimental Total	Control Events	Control Total	Weight	Risk Ratio M-H, Random, 95% CI
**2.1.1 mRNA vaccine**						
Ali 2021	2381	2482	806	1238	24.4%	1.47 [1.41, 1.54]
Anderson 2022	1592	1895	460	582	24.3%	1.06 [1.01, 1.11]
Buddy Creech 2022	3217	3382	681	993	24.4%	1.39 [1.33, 1.45]
**Subtotal (95% CI)**		**7759**		**2813**	**73.1%**	**1.30 [1.07, 1.57]**
Total events	7190		1947			
Heterogeneity: Tau² = 0.03; Chi² = 118.89, df = 2 (P < 0.00001); I² = 98%						
Test for overall effect: Z = 2.62 (P = 0.009)						
**2.1.2 Inactivated vaccine**						
Han 2021	73	436	15	114	7.5%	1.27 [0.76, 2.13]
**Subtotal (95% CI)**		**436**		**114**	**7.5%**	**1.27 [0.76, 2.13]**
Total events	73		15			
Heterogeneity: Not applicable						
Test for overall effect: Z = 0.92 (P = 0.36)						
**2.1.3 subunit vaccine**						
Liu 2022	240	341	26	58	14.2%	1.57 [1.17, 2.11]
**Subtotal (95% CI)**		**341**		**58**	**14.2%**	**1.57 [1.17, 2.11]**
Total events	240		26			
Heterogeneity: Not applicable						
Test for overall effect: Z = 3.01 (P = 0.003)						
**2.1.4 Adenovirus vaccine**						
Zhu 2021	55	100	8	50	5.2%	3.44 [1.78, 6.65]
**Subtotal (95% CI)**		**100**		**50**	**5.2%**	**3.44 [1.78, 6.65]**
Total events	55		8			
Heterogeneity: Not applicable						
Test for overall effect: Z = 3.67 (P = 0.0002)						
**Total (95% CI)**		**8636**		**3035**	**100.0%**	**1.40 [1.18, 1.66]**
Total events	7558		1996			
Heterogeneity: Tau² = 0.03; Chi² = 130.12, df = 5 (P < 0.00001); I² = 96%						
Test for overall effect: Z = 3.88 (P = 0.0001)						
Test for subgroup differences: Chi² = 8.38, df = 3 (P = 0.04), I² = 64.2%						

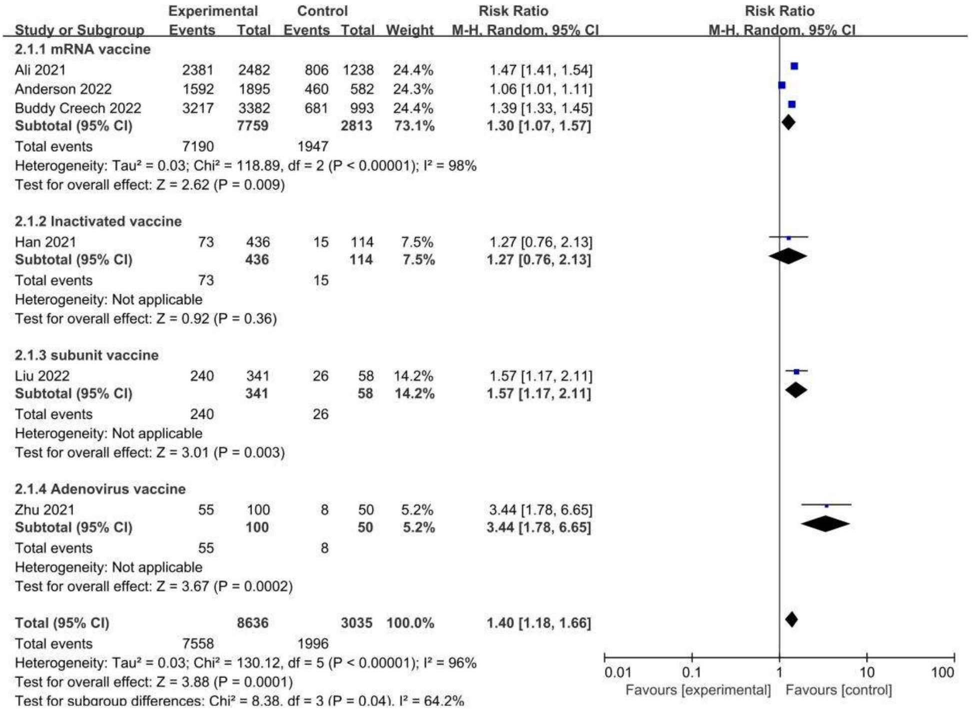

B) Total adverse reactions after dose 2

Study or Subgroup	Experimental Events	Experimental Total	Control Events	Control Total	Weight	Risk Ratio M-H, Random, 95% CI
**2.2.1 mRNA vaccine**						
Ali 2021	2405	2478	680	1220	26.5%	1.74 [1.66, 1.83]
Anderson 2022	1451	1746	381	526	26.3%	1.15 [1.08, 1.21]
Buddy Creech 2022	3262	3367	646	969	26.5%	1.45 [1.39, 1.52]
**Subtotal (95% CI)**		**7591**		**2715**	**79.3%**	**1.43 [1.14, 1.79]**
Total events	7118		1707			
Heterogeneity: Tau² = 0.04; Chi² = 118.58, df = 2 (P < 0.00001); I² = 98%						
Test for overall effect: Z = 3.10 (P = 0.002)						
**2.2.2 Inactivated vaccine**						
Han 2021	56	425	8	111	6.3%	1.83 [0.90, 3.72]
**Subtotal (95% CI)**		**425**		**111**	**6.3%**	**1.83 [0.90, 3.72]**
Total events	56		8			
Heterogeneity: Not applicable						
Test for overall effect: Z = 1.66 (P = 0.10)						
**2.2.3 subunit vaccine**						
Liu 2022	182	340	16	58	12.3%	1.94 [1.26, 2.98]
**Subtotal (95% CI)**		**340**		**58**	**12.3%**	**1.94 [1.26, 2.98]**
Total events	182		16			
Heterogeneity: Not applicable						
Test for overall effect: Z = 3.03 (P = 0.002)						
**2.2.4 Adenovirus vaccine**						
Zhu 2021	33	100	2	50	2.0%	8.25 [2.06, 33.00]
**Subtotal (95% CI)**		**100**		**50**	**2.0%**	**8.25 [2.06, 33.00]**
Total events	33		2			
Heterogeneity: Not applicable						
Test for overall effect: Z = 2.98 (P = 0.003)						
**Total (95% CI)**		**8456**		**2934**	**100.0%**	**1.56 [1.27, 1.91]**
Total events	7389		1733			
Heterogeneity: Tau² = 0.04; Chi² = 128.52, df = 5 (P < 0.00001); I² = 96%						
Test for overall effect: Z = 4.26 (P < 0.0001)						
Test for subgroup differences: Chi² = 7.29, df = 3 (P = 0.06), I² = 58.8%						

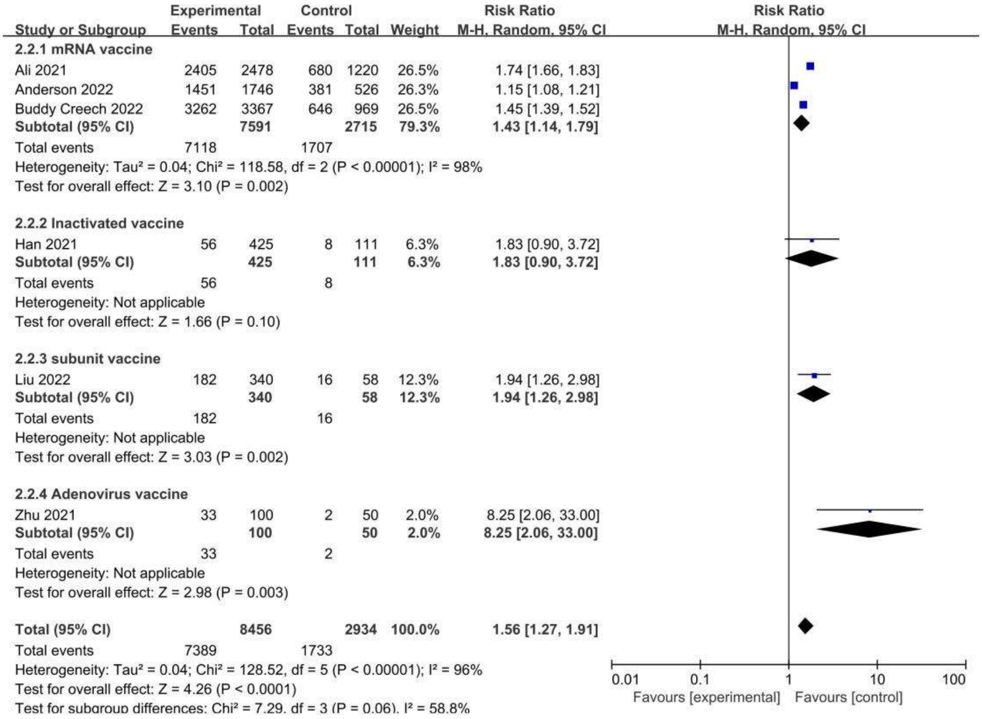

C) Systemic adverse reactions after dose 1

Study or Subgroup	Experimental Events	Experimental Total	Control Events	Control Total	Weight	Risk Ratio M-H, Random, 95% CI
**2.3.1 mRNA vaccine**						
Ali 2021	1701	2482	687	1238	29.2%	1.23 [1.17, 1.31]
Anderson 2022	1441	1894	421	582	29.2%	1.05 [0.99, 1.11]
Buddy Creech 2022	1947	3382	518	993	28.3%	1.10 [1.03, 1.18]
**Subtotal (95% CI)**		**7758**		**2813**	**86.6%**	**1.13 [1.02, 1.24]**
Total events	5089		1626			
Heterogeneity: Tau² = 0.01; Chi² = 16.52, df = 2 (P = 0.0003); I² = 88%						
Test for overall effect: Z = 2.39 (P = 0.02)						
**2.3.2 Inactivated vaccine**						
Xia 2021	99	755	25	252	5.2%	1.32 [0.87, 2.00]
**Subtotal (95% CI)**		**755**		**252**	**5.2%**	**1.32 [0.87, 2.00]**
Total events	99		25			
Heterogeneity: Not applicable						
Test for overall effect: Z = 1.32 (P = 0.19)						
**2.3.3 subunit vaccine**						
Liu 2022	143	341	22	58	6.8%	1.11 [0.78, 1.57]
**Subtotal (95% CI)**		**341**		**58**	**6.8%**	**1.11 [0.78, 1.57]**
Total events	143		22			
Heterogeneity: Not applicable						
Test for overall effect: Z = 0.56 (P = 0.58)						
**2.3.4 Adenovirus vaccine**						
Zhu 2021	37	100	5	50	1.4%	3.70 [1.55, 8.83]
**Subtotal (95% CI)**		**100**		**50**	**1.4%**	**3.70 [1.55, 8.83]**
Total events	37		5			
Heterogeneity: Not applicable						
Test for overall effect: Z = 2.95 (P = 0.003)						
**Total (95% CI)**		**8954**		**3173**	**100.0%**	**1.15 [1.04, 1.28]**
Total events	5368		1678			
Heterogeneity: Tau² = 0.01; Chi² = 24.66, df = 5 (P = 0.0002); I² = 80%						
Test for overall effect: Z = 2.71 (P = 0.007)						
Test for subgroup differences: Chi² = 7.56, df = 3 (P = 0.06), I² = 60.3%						

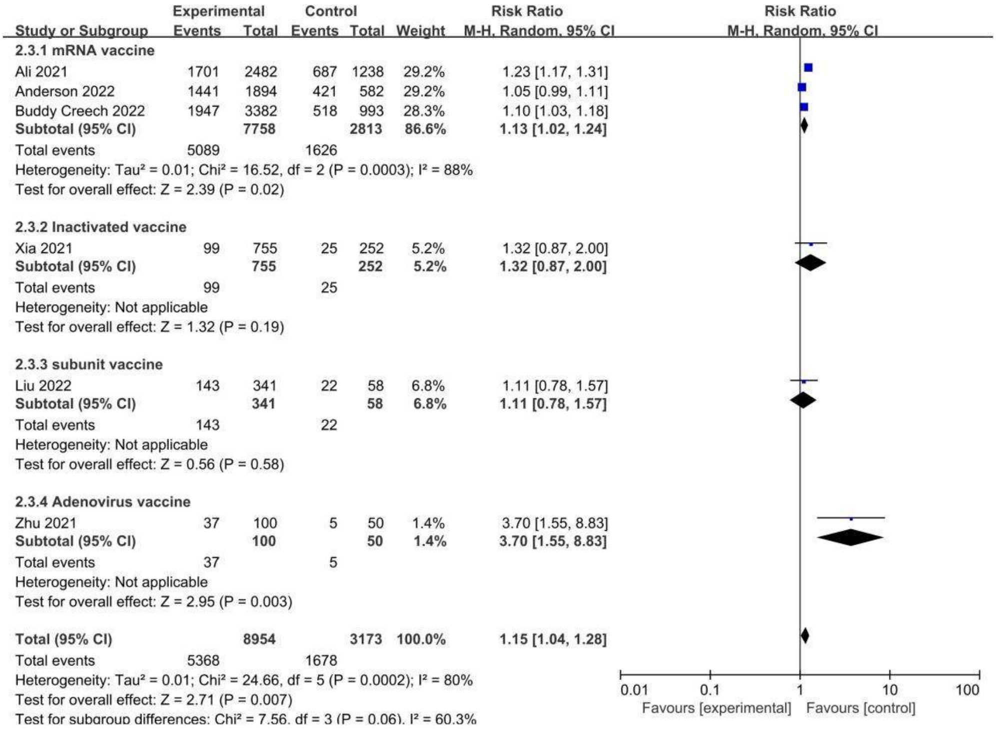

D) Systemic adverse reactions after dose 2

Study or Subgroup	Experimental Events	Experimental Total	Control Events	Control Total	Weight	Risk Ratio M-H, Random, 95% CI
**2.4.1 mRNA vaccine**						
Ali 2021	2134	2478	561	1220	25.2%	1.87 [1.76, 1.99]
Anderson 2022	1278	1746	350	526	25.2%	1.10 [1.03, 1.18]
Buddy Creech 2022	2619	3367	485	969	25.2%	1.55 [1.46, 1.66]
**Subtotal (95% CI)**		**7591**		**2715**	**75.5%**	**1.47 [1.08, 2.01]**
Total events	6031		1396			
Heterogeneity: Tau² = 0.07; Chi² = 135.95, df = 2 (P < 0.00001); I² = 99%						
Test for overall effect: Z = 2.46 (P = 0.01)						
**2.4.2 Inactivated vaccine**						
Xia 2021	39	751	8	248	8.6%	1.61 [0.76, 3.40]
**Subtotal (95% CI)**		**751**		**248**	**8.6%**	**1.61 [0.76, 3.40]**
Total events	39		8			
Heterogeneity: Not applicable						
Test for overall effect: Z = 1.25 (P = 0.21)						
**2.4.3 subunit vaccine**						
Liu 2022	86	340	12	58	12.7%	1.22 [0.72, 2.09]
**Subtotal (95% CI)**		**340**		**58**	**12.7%**	**1.22 [0.72, 2.09]**
Total events	86		12			
Heterogeneity: Not applicable						
Test for overall effect: Z = 0.73 (P = 0.46)						
**2.4.4 Adenovirus vaccine**						
Zhu 2021	24	100	2	50	3.2%	6.00 [1.48, 24.38]
**Subtotal (95% CI)**		**100**		**50**	**3.2%**	**6.00 [1.48, 24.38]**
Total events	24		2			
Heterogeneity: Not applicable						
Test for overall effect: Z = 2.50 (P = 0.01)						
**Total (95% CI)**		**8782**		**3071**	**100.0%**	**1.52 [1.16, 1.99]**
Total events	6180		1418			
Heterogeneity: Tau² = 0.07; Chi² = 140.71, df = 5 (P < 0.00001); I² = 96%						
Test for overall effect: Z = 3.04 (P = 0.002)						
Test for subgroup differences: Chi² = 4.36, df = 3 (P = 0.23), I² = 31.2%						

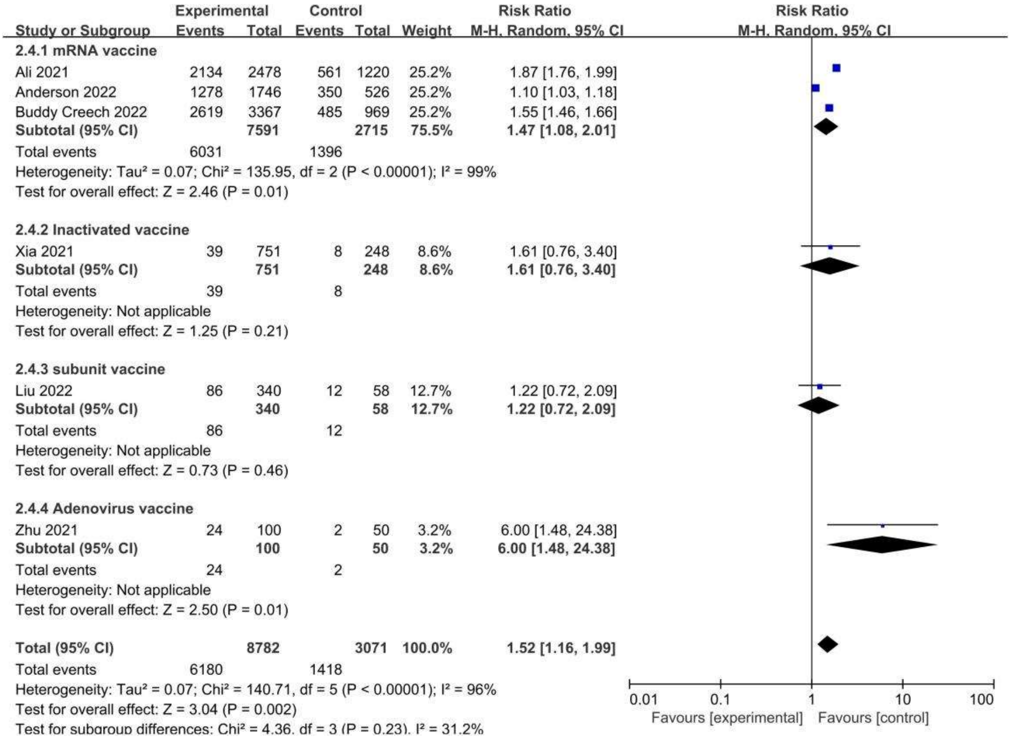

E) Local adverse reactions after dose 1

Study or Subgroup	Experimental Events	Experimental Total	Control Events	Control Total	Weight	Risk Ratio M-H, Random, 95% CI
**2.5.1 mRNA vaccine**						
Ali 2021	2339	2482	455	1238	22.9%	2.56 [2.38, 2.76]
Anderson 2022	2376	3026	641	970	23.0%	1.19 [1.13, 1.25]
Buddy Creech 2022	3153	3382	480	993	22.9%	1.93 [1.81, 2.06]
**Subtotal (95% CI)**		**8890**		**3201**	**68.7%**	**1.80 [1.11, 2.92]**
Total events	7868		1576			
Heterogeneity: Tau² = 0.18; Chi² = 365.58, df = 2 (P < 0.00001); I² = 99%						
Test for overall effect: Z = 2.40 (P = 0.02)						
**2.5.2 Inactivated vaccine**						
Xia 2021	38	755	2	252	6.1%	6.34 [1.54, 26.10]
**Subtotal (95% CI)**		**755**		**252**	**6.1%**	**6.34 [1.54, 26.10]**
Total events	38		2			
Heterogeneity: Not applicable						
Test for overall effect: Z = 2.56 (P = 0.01)						
**2.5.3 subunit vaccine**						
Liu 2022	207	341	12	58	16.9%	2.93 [1.76, 4.89]
**Subtotal (95% CI)**		**341**		**58**	**16.9%**	**2.93 [1.76, 4.89]**
Total events	207		12			
Heterogeneity: Not applicable						
Test for overall effect: Z = 4.13 (P < 0.0001)						
**2.5.4 Adenovirus vaccine**						
Zhu 2021	36	100	3	50	8.3%	6.00 [1.94, 18.53]
**Subtotal (95% CI)**		**100**		**50**	**8.3%**	**6.00 [1.94, 18.53]**
Total events	36		3			
Heterogeneity: Not applicable						
Test for overall effect: Z = 3.11 (P = 0.002)						
**Total (95% CI)**		**10086**		**3561**	**100.0%**	**2.33 [1.56, 3.50]**
Total events	8149		1593			
Heterogeneity: Tau² = 0.19; Chi² = 385.40, df = 5 (P < 0.00001); I² = 99%						
Test for overall effect: Z = 4.09 (P < 0.0001)						
Test for subgroup differences: Chi² = 6.06, df = 3 (P = 0.11), I² = 50.5%						

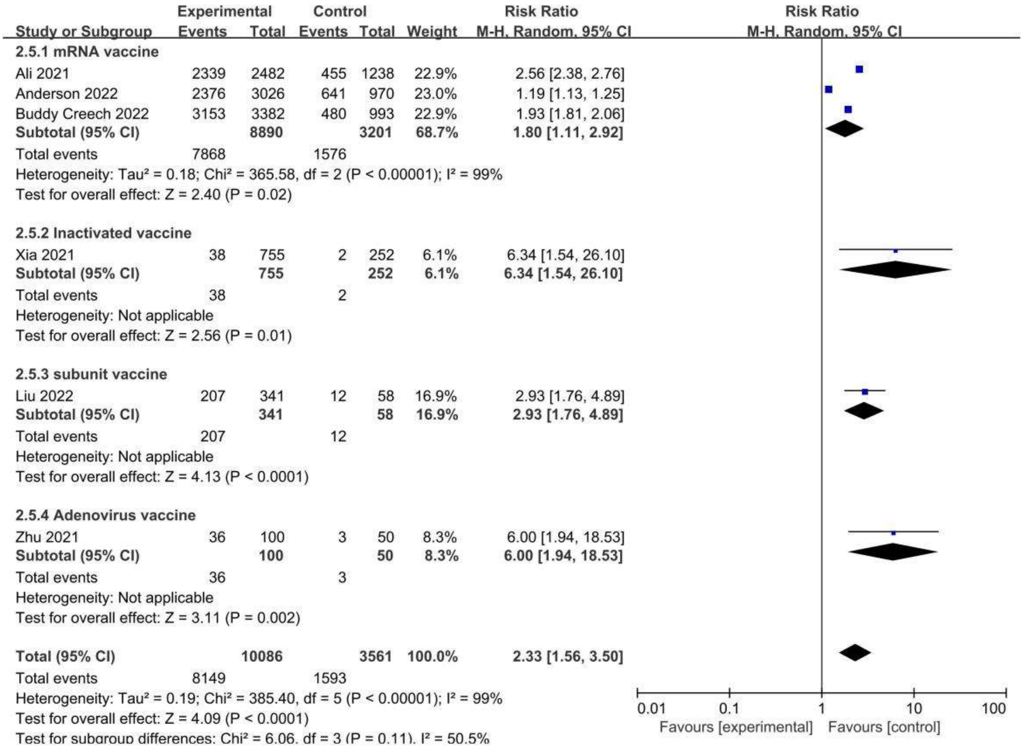

F) Local adverse reactions after dose 2

Study or Subgroup	Experimental Events	Experimental Total	Control Events	Control Total	Weight	Risk Ratio M-H, Random, 95% CI
**2.6.1 mRNA vaccine**						
Ali 2021	2314	2478	398	1220	24.9%	2.86 [2.64, 3.10]
Anderson 2022	2536	3007	603	959	25.1%	1.34 [1.27, 1.41]
Buddy Creech 2022	3204	3367	490	969	25.0%	1.88 [1.77, 2.00]
**Subtotal (95% CI)**		**8852**		**3148**	**74.9%**	**1.93 [1.25, 2.97]**
Total events	8054		1491			
Heterogeneity: Tau² = 0.14; Chi² = 275.15, df = 2 (P < 0.00001); I² = 99%						
Test for overall effect: Z = 2.99 (P = 0.003)						
**2.6.2 Inactivated vaccine**						
Xia 2021	26	751	2	248	5.4%	4.29 [1.03, 17.96]
**Subtotal (95% CI)**		**751**		**248**	**5.4%**	**4.29 [1.03, 17.96]**
Total events	26		2			
Heterogeneity: Not applicable						
Test for overall effect: Z = 2.00 (P = 0.05)						
**2.6.3 subunit vaccine**						
Liu 2022	163	340	14	58	18.0%	1.99 [1.24, 3.18]
**Subtotal (95% CI)**		**340**		**58**	**18.0%**	**1.99 [1.24, 3.18]**
Total events	163		14			
Heterogeneity: Not applicable						
Test for overall effect: Z = 2.86 (P = 0.004)						
**2.6.4 Adenovirus vaccine**						
Zhu 2021	19	100	0	50	1.7%	19.69 [1.21, 319.62]
**Subtotal (95% CI)**		**100**		**50**	**1.7%**	**19.69 [1.21, 319.62]**
Total events	19		0			
Heterogeneity: Not applicable						
Test for overall effect: Z = 2.10 (P = 0.04)						
**Total (95% CI)**		**10043**		**3504**	**100.0%**	**2.11 [1.45, 3.06]**
Total events	8262		1507			
Heterogeneity: Tau² = 0.14; Chi² = 281.97, df = 5 (P < 0.00001); I² = 98%						
Test for overall effect: Z = 3.91 (P < 0.0001)						
Test for subgroup differences: Chi² = 3.63, df = 3 (P = 0.30), I² = 17.4%						

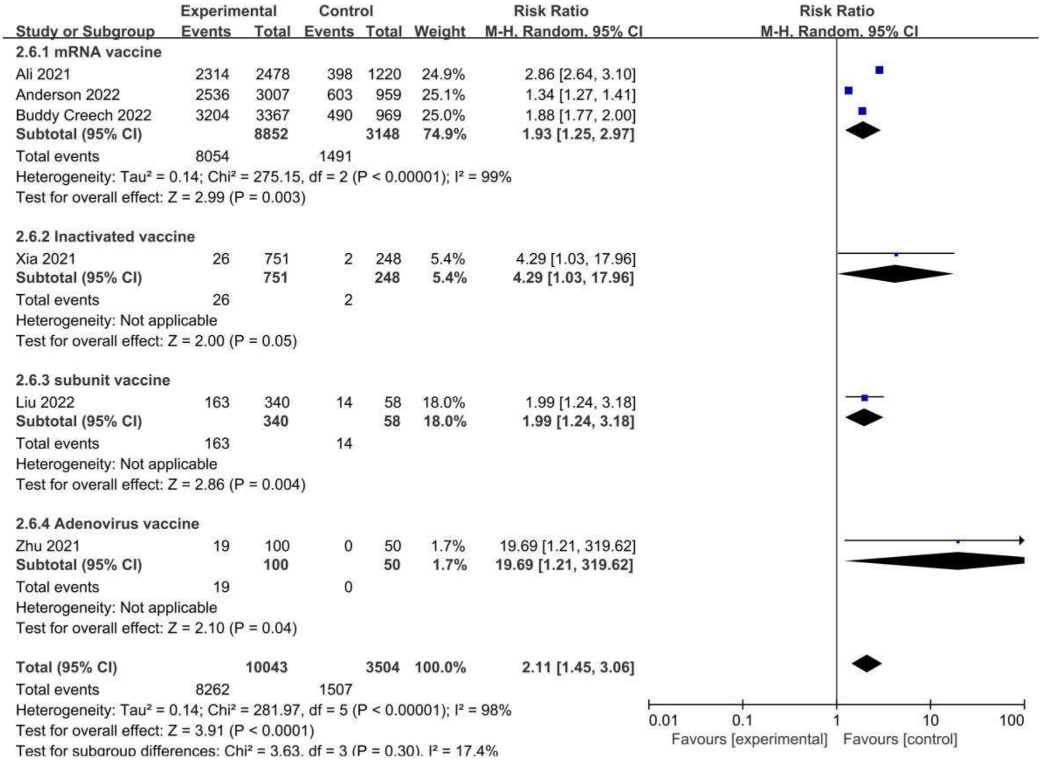

Figure S5. Specific adverse reactions in the mRNA vaccine group versus the control group:

A) After dose 1

Study or Subgroup	Experimental Events	Experimental Total	Control Events	Control Total	Weight	Risk Ratio M-H, Random, 95% CI
**2.7.1 Local pain (local adverse reactions)**						
Ali 2021	2310	2482	431	1238	2.2%	2.67 [2.48, 2.89]
Anderson 2022	2552	4916	557	1552	2.2%	1.45 [1.35, 1.55]
Buddy Creech 2022	3132	3382	465	993	2.2%	1.98 [1.85, 2.11]
Frenck 2021	972	1131	260	1129	2.2%	3.73 [3.35, 4.16]
Walter 2021	1124	1518	233	750	2.2%	2.38 [2.13, 2.66]
**Subtotal (95% CI)**		**13429**		**5662**	**11.1%**	**2.32 [1.72, 3.13]**
Total events	10090		1946			
Heterogeneity: $Tau^2 = 0.11$; $Chi^2 = 256.03$, df = 4 (P < 0.00001); $I^2 = 98\%$						
Test for overall effect: Z = 5.55 (P < 0.00001)						
**2.7.2 Erythema/Redness (local adverse reactions)**						
Ali 2021	334	2482	8	1238	1.3%	20.82 [10.36, 41.85]
Anderson 2022	329	4917	38	1552	1.9%	2.73 [1.96, 3.80]
Buddy Creech 2022	393	3382	13	993	1.5%	8.88 [5.13, 15.35]
Frenck 2021	68	1131	12	1129	1.4%	5.66 [3.08, 10.39]
Walter 2021	228	1518	45	750	1.9%	2.50 [1.84, 3.40]
**Subtotal (95% CI)**		**13430**		**5662**	**8.1%**	**5.66 [2.75, 11.65]**
Total events	1352		116			
Heterogeneity: $Tau^2 = 0.61$; $Chi^2 = 50.81$, df = 4 (P < 0.00001); $I^2 = 92\%$						
Test for overall effect: Z = 4.70 (P < 0.00001)						
**2.7.3 Swelling / Hardness (local adverse reactions)**						
Ali 2021	403	2482	12	1238	1.5%	16.75 [9.47, 29.62]
Anderson 2022	298	4917	32	1552	1.9%	2.94 [2.05, 4.21]
Buddy Creech 2022	394	3382	12	993	1.5%	9.64 [5.45, 17.05]
Frenck 2021	80	1131	12	1129	1.4%	6.65 [3.65, 12.14]
Walter 2021	152	1518	23	750	1.7%	3.27 [2.12, 5.02]
**Subtotal (95% CI)**		**13430**		**5662**	**8.0%**	**6.21 [3.14, 12.28]**
Total events	1327		91			
Heterogeneity: $Tau^2 = 0.54$; $Chi^2 = 38.61$, df = 4 (P < 0.00001); $I^2 = 90\%$						
Test for overall effect: Z = 5.26 (P < 0.00001)						
**2.7.4 Axillary Swelling (local adverse reactions)**						
Ali 2021	578	2482	101	1238	2.1%	2.85 [2.34, 3.49]
Anderson 2022	323	4915	82	1552	2.1%	1.24 [0.98, 1.57]
Buddy Creech 2022	506	3382	84	993	2.1%	1.77 [1.42, 2.20]
**Subtotal (95% CI)**		**10779**		**3783**	**6.2%**	**1.85 [1.15, 2.98]**
Total events	1407		267			
Heterogeneity: $Tau^2 = 0.17$; $Chi^2 = 28.80$, df = 2 (P < 0.00001); $I^2 = 93\%$						
Test for overall effect: Z = 2.53 (P = 0.01)						
**2.7.5 Fever (systemic adverse reactions)**						
Ali 2021	63	2480	12	1238	1.4%	2.62 [1.42, 4.84]
Anderson 2022	202	1892	49	582	2.0%	1.27 [0.94, 1.71]
Buddy Creech 2022	110	3381	15	993	1.5%	2.15 [1.26, 3.68]
Frenck 2021	114	1131	12	1129	1.4%	9.48 [5.26, 17.09]
Walter 2021	107	1518	8	750	1.2%	6.61 [3.24, 13.48]
**Subtotal (95% CI)**		**10402**		**4692**	**7.6%**	**3.31 [1.47, 7.45]**
Total events	596		96			
Heterogeneity: $Tau^2 = 0.77$; $Chi^2 = 49.10$, df = 4 (P < 0.00001); $I^2 = 92\%$						
Test for overall effect: Z = 2.89 (P = 0.004)						
**2.7.6 Headache (systemic adverse reactions)**						
Ali 2021	1106	2480	477	1238	2.2%	1.16 [1.07, 1.26]
Buddy Creech 2022	1047	3380	306	993	2.2%	1.01 [0.90, 1.12]
Frenck 2021	623	1131	396	1129	2.2%	1.57 [1.43, 1.73]
Walter 2021	334	1518	180	750	2.2%	0.92 [0.78, 1.07]
**Subtotal (95% CI)**		**8509**		**4110**	**8.8%**	**1.14 [0.92, 1.43]**
Total events	3110		1359			
Heterogeneity: $Tau^2 = 0.05$; $Chi^2 = 53.44$, df = 3 (P < 0.00001); $I^2 = 94\%$						
Test for overall effect: Z = 1.18 (P = 0.24)						
**2.7.7 Fatigue (systemic adverse reactions)**						
Ali 2021	1188	2481	453	1238	2.2%	1.31 [1.20, 1.42]
Buddy Creech 2022	1452	3380	334	993	2.2%	1.28 [1.16, 1.41]
Frenck 2021	679	1131	463	1129	2.2%	1.46 [1.35, 1.59]
Walter 2021	517	1518	233	750	2.2%	1.10 [0.97, 1.25]
**Subtotal (95% CI)**		**8510**		**4110**	**8.8%**	**1.29 [1.16, 1.43]**
Total events	3836		1483			
Heterogeneity: $Tau^2 = 0.01$; $Chi^2 = 14.50$, df = 3 (P = 0.002); $I^2 = 79\%$						
Test for overall effect: Z = 4.72 (P < 0.00001)						
**2.7.8 Myalgia (systemic adverse reactions)**						
Ali 2021	668	2480	205	1238	2.2%	1.63 [1.41, 1.87]
Buddy Creech 2022	478	3380	96	993	2.1%	1.46 [1.19, 1.80]
Frenck 2021	272	1131	147	1129	2.1%	1.85 [1.54, 2.22]
Walter 2021	137	1518	53	750	2.0%	1.28 [0.94, 1.73]
**Subtotal (95% CI)**		**8509**		**4110**	**8.4%**	**1.59 [1.39, 1.81]**
Total events	1555		501			
Heterogeneity: $Tau^2 = 0.01$; $Chi^2 = 5.24$, df = 3 (P = 0.16); $I^2 = 43\%$						
Test for overall effect: Z = 6.96 (P < 0.00001)						
**2.7.9 Arthralgia (systemic adverse reactions)**						
Ali 2021	371	2480	143	1238	2.1%	1.30 [1.08, 1.55]
Buddy Creech 2022	287	3380	75	993	2.0%	1.12 [0.88, 1.44]
Frenck 2021	114	1131	80	1129	2.0%	1.42 [1.08, 1.87]
Walter 2021	46	1518	38	750	1.8%	0.60 [0.39, 0.91]
**Subtotal (95% CI)**		**8509**		**4110**	**7.9%**	**1.10 [0.84, 1.45]**
Total events	818		336			
Heterogeneity: $Tau^2 = 0.06$; $Chi^2 = 12.96$, df = 3 (P = 0.005); $I^2 = 77\%$						
Test for overall effect: Z = 0.71 (P = 0.48)						
**2.7.10 Nausea / vomiting(systemic adverse reactions)**						
Ali 2021	281	2480	110	1238	2.1%	1.28 [1.03, 1.57]
Buddy Creech 2022	361	3380	107	993	2.1%	0.99 [0.81, 1.22]
Frenck 2021	34	1131	12	1129	1.3%	2.83 [1.47, 5.43]
Walter 2021	31	1518	8	750	1.2%	1.91 [0.88, 4.14]
**Subtotal (95% CI)**		**8509**		**4110**	**6.7%**	**1.41 [0.99, 1.99]**
Total events	707		237			
Heterogeneity: $Tau^2 = 0.08$; $Chi^2 = 11.78$, df = 3 (P = 0.008); $I^2 = 75\%$						
Test for overall effect: Z = 1.93 (P = 0.05)						
**2.7.11 Chills （systemic adverse reactions）**						
Ali 2021	456	2480	138	1238	2.1%	1.65 [1.38, 1.97]
Buddy Creech 2022	342	3380	67	993	2.0%	1.50 [1.17, 1.93]
Frenck 2021	317	1131	120	1129	2.1%	2.64 [2.17, 3.20]
Walter 2021	76	1518	38	750	1.8%	0.99 [0.68, 1.44]
**Subtotal (95% CI)**		**8509**		**4110**	**8.1%**	**1.63 [1.15, 2.33]**
Total events	1191		363			
Heterogeneity: $Tau^2 = 0.11$; $Chi^2 = 27.54$, df = 3 (P < 0.00001); $I^2 = 89\%$						
Test for overall effect: Z = 2.71 (P = 0.007)						
**2.7.12 Diarrhea（systemic adverse reactions）**						
Frenck 2021	91	1131	80	1129	2.0%	1.14 [0.85, 1.52]
Walter 2021	92	1518	30	750	1.8%	1.52 [1.01, 2.27]
**Subtotal (95% CI)**		**2649**		**1879**	**3.8%**	**1.27 [0.96, 1.67]**
Total events	183		110			
Heterogeneity: $Tau^2 = 0.01$; $Chi^2 = 1.31$, df = 1 (P = 0.25); $I^2 = 23\%$						
Test for overall effect: Z = 1.68 (P = 0.09)						
**2.7.13 Irritability/Crying（systemic adverse reactions）**						
Anderson 2022	1269	1886	361	581	2.2%	1.08 [1.01, 1.16]
**Subtotal (95% CI)**		**1886**		**581**	**2.2%**	**1.08 [1.01, 1.16]**
Total events	1269		361			
Heterogeneity: Not applicable						
Test for overall effect: Z = 2.20 (P = 0.03)						
**2.7.14 Sleepiness (systemic adverse reactions)**						
Anderson 2022	684	1888	217	581	2.2%	0.97 [0.86, 1.09]
**Subtotal (95% CI)**		**1888**		**581**	**2.2%**	**0.97 [0.86, 1.09]**
Total events	684		217			
Heterogeneity: Not applicable						
Test for overall effect: Z = 0.49 (P = 0.62)						
**2.7.15 Loss of Appetite（systemic adverse reactions）**						
Anderson 2022	552	1886	152	581	2.2%	1.12 [0.96, 1.30]
**Subtotal (95% CI)**		**1886**		**581**	**2.2%**	**1.12 [0.96, 1.30]**
Total events	552		152			
Heterogeneity: Not applicable						
Test for overall effect: Z = 1.43 (P = 0.15)						
**Total (95% CI)**		**120834**		**53743**	**100.0%**	**1.91 [1.70, 2.16]**
Total events	28677		7635			
Heterogeneity: $Tau^2 = 0.17$; $Chi^2 = 1477.82$, df = 51 (P < 0.00001); $I^2 = 97\%$						
Test for overall effect: Z = 10.66 (P < 0.00001)						
Test for subgroup differences: $Chi^2 = 112.64$, df = 14 (P < 0.00001), $I^2 = 87.6\%$						

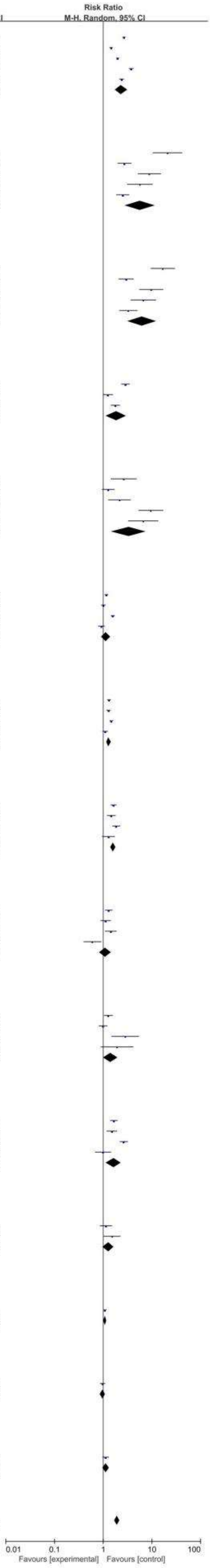

B) After dose 2

Study or Subgroup	Experimental Events	Experimental Total	Control Events	Control Total	Weight	Risk Ratio M-H, Random, 95% CI
**2.8.1 Local pain（local adverse reactions）**						
Ali 2021	2290	2478	370	1220	2.2%	3.05 [2.80, 3.32]
Anderson 2022	2949	4753	530	1485	2.2%	1.74 [1.62, 1.87]
Buddy Creech 2022	3182	3367	480	969	2.2%	1.91 [1.79, 2.03]
Frenck 2021	894	1131	204	1129	2.1%	4.37 [3.85, 4.97]
Walter 2021	1075	1514	217	747	2.1%	2.44 [2.18, 2.75]
**Subtotal (95% CI)**		**13243**		**5550**	**10.7%**	**2.54 [1.89, 3.42]**
Total events	10390		1801			
Heterogeneity: Tau² = 0.11; Chi² = 233.39, df = 4 (P < 0.00001); I² = 98%						
Test for overall effect: Z = 6.17 (P < 0.00001)						
**2.8.2 Erythema/Redness（local adverse reactions）**						
Ali 2021	484	2478	11	1220	1.5%	21.66 [11.96, 39.22]
Anderson 2022	500	4753	35	1485	1.9%	4.46 [3.18, 6.26]
Buddy Creech 2022	640	3367	10	969	1.5%	18.42 [9.90, 34.26]
Frenck 2021	57	1131	12	1129	1.5%	4.74 [2.56, 8.79]
Walter 2021	288	1514	38	747	1.9%	3.74 [2.70, 5.18]
**Subtotal (95% CI)**		**13243**		**5550**	**8.4%**	**7.73 [3.76, 15.90]**
Total events	1969		106			
Heterogeneity: Tau² = 0.61; Chi² = 48.55, df = 4 (P < 0.00001); I² = 92%						
Test for overall effect: Z = 5.56 (P < 0.00001)						
**2.8.3 Swelling / Hardness（local adverse reactions）**						
Ali 2021	509	2478	12	1220	1.6%	20.88 [11.83, 36.86]
Anderson 2022	507	4753	22	1485	1.8%	7.20 [4.72, 10.99]
Buddy Creech 2022	588	3367	12	969	1.6%	14.10 [8.00, 24.86]
Frenck 2021	57	1131	12	1129	1.5%	4.74 [2.56, 8.79]
Walter 2021	228	1514	23	747	1.8%	4.89 [3.21, 7.44]
**Subtotal (95% CI)**		**13243**		**5550**	**8.3%**	**8.59 [4.86, 15.19]**
Total events	1889		81			
Heterogeneity: Tau² = 0.35; Chi² = 25.19, df = 4 (P < 0.0001); I² = 84%						
Test for overall effect: Z = 7.39 (P < 0.00001)						
**2.8.4 Axillary Swelling (local adverse reactions)**						
Ali 2021	519	2477	61	1220	2.0%	4.19 [3.24, 5.41]
Anderson 2022	427	4753	59	1485	2.0%	2.26 [1.73, 2.95]
Buddy Creech 2022	583	3367	65	969	2.0%	2.58 [2.02, 3.30]
**Subtotal (95% CI)**		**10597**		**3674**	**6.1%**	**2.90 [2.02, 4.18]**
Total events	1529		185			
Heterogeneity: Tau² = 0.09; Chi² = 12.19, df = 2 (P = 0.002); I² = 84%						
Test for overall effect: Z = 5.73 (P < 0.00001)						
**2.8.5 Fever (systemic adverse reactions)**						
Ali 2021	302	2477	12	1219	1.6%	12.39 [6.98, 21.96]
Anderson 2022	249	1744	44	526	2.0%	1.71 [1.26, 2.32]
Buddy Creech 2022	792	3367	19	969	1.8%	12.00 [7.65, 18.80]
Frenck 2021	227	1131	12	1129	1.6%	18.88 [10.63, 33.55]
Walter 2021	106	1514	8	747	1.4%	6.54 [3.20, 13.34]
**Subtotal (95% CI)**		**10233**		**4590**	**8.2%**	**7.85 [2.58, 23.91]**
Total events	1676		95			
Heterogeneity: Tau² = 1.54; Chi² = 103.79, df = 4 (P < 0.00001); I² = 96%						
Test for overall effect: Z = 3.63 (P = 0.0003)						
**2.8.6 Headache (systemic adverse reactions)**						
Ali 2021	1739	2478	370	1220	2.2%	2.31 [2.12, 2.53]
Buddy Creech 2022	1740	3365	275	969	2.1%	1.82 [1.64, 2.02]
Frenck 2021	736	1131	271	1129	2.1%	2.71 [2.42, 3.03]
Walter 2021	424	1514	142	747	2.1%	1.47 [1.24, 1.74]
**Subtotal (95% CI)**		**8488**		**4065**	**8.5%**	**2.04 [1.63, 2.56]**
Total events	4639		1058			
Heterogeneity: Tau² = 0.05; Chi² = 47.97, df = 3 (P < 0.00001); I² = 94%						
Test for overall effect: Z = 6.16 (P < 0.00001)						
**2.8.7 Fatigue (systemic adverse reactions)**						
Ali 2021	1679	2478	353	1220	2.2%	2.34 [2.14, 2.57]
Buddy Creech 2022	2141	3365	335	969	2.2%	1.84 [1.68, 2.01]
Frenck 2021	747	1131	283	1129	2.1%	2.63 [2.36, 2.94]
Walter 2021	591	1514	180	747	2.1%	1.62 [1.41, 1.87]
**Subtotal (95% CI)**		**8488**		**4065**	**8.6%**	**2.08 [1.70, 2.54]**
Total events	5158		1151			
Heterogeneity: Tau² = 0.04; Chi² = 42.94, df = 3 (P < 0.00001); I² = 93%						
Test for overall effect: Z = 7.18 (P < 0.00001)						
**2.8.8 Myalgia (systemic adverse reactions)**						
Ali 2021	1154	2477	153	1220	2.1%	3.71 [3.18, 4.33]
Buddy Creech 2022	932	3365	105	969	2.1%	2.56 [2.12, 3.09]
Frenck 2021	362	1131	91	1129	2.1%	3.97 [3.20, 4.92]
Walter 2021	182	1514	53	747	2.0%	1.69 [1.26, 2.27]
**Subtotal (95% CI)**		**8487**		**4065**	**8.2%**	**2.87 [2.07, 3.98]**
Total events	2630		402			
Heterogeneity: Tau² = 0.10; Chi² = 30.75, df = 3 (P < 0.00001); I² = 90%						
Test for overall effect: Z = 6.32 (P < 0.00001)						
**2.8.9 Arthralgia (systemic adverse reactions)**						
Ali 2021	716	2477	113	1220	2.1%	3.12 [2.59, 3.76]
Buddy Creech 2022	525	3365	84	969	2.1%	1.80 [1.45, 2.24]
Frenck 2021	181	1131	57	1129	2.0%	3.17 [2.38, 4.22]
Walter 2021	76	1514	30	747	1.8%	1.25 [0.83, 1.89]
**Subtotal (95% CI)**		**8487**		**4065**	**7.9%**	**2.22 [1.50, 3.28]**
Total events	1498		284			
Heterogeneity: Tau² = 0.14; Chi² = 27.45, df = 3 (P < 0.00001); I² = 89%						
Test for overall effect: Z = 4.02 (P < 0.0001)						
**2.8.10 Nausea / vomiting (systemic adverse reactions)**						
Ali 2021	591	2447	106	1220	2.1%	2.78 [2.29, 3.38]
Buddy Creech 2022	795	3365	97	969	2.1%	2.36 [1.94, 2.88]
Frenck 2021	34	1131	12	1129	1.4%	2.83 [1.47, 5.43]
Walter 2021	31	1514	8	747	1.3%	1.91 [0.88, 4.14]
**Subtotal (95% CI)**		**8457**		**4065**	**6.9%**	**2.55 [2.23, 2.92]**
Total events	1451		223			
Heterogeneity: Tau² = 0.00; Chi² = 1.97, df = 3 (P = 0.58); I² = 0%						
Test for overall effect: Z = 13.72 (P < 0.00001)						
**2.8.11 Chills (systemic adverse reactions)**						
Ali 2021	1066	2477	91	1220	2.1%	5.77 [4.71, 7.07]
Buddy Creech 2022	981	3365	74	969	2.1%	3.82 [3.05, 4.78]
Frenck 2021	476	1131	80	1129	2.1%	5.94 [4.76, 7.42]
Walter 2021	152	1514	30	747	1.9%	2.50 [1.71, 3.66]
**Subtotal (95% CI)**		**8487**		**4065**	**8.0%**	**4.37 [3.14, 6.09]**
Total events	2675		275			
Heterogeneity: Tau² = 0.10; Chi² = 21.90, df = 3 (P < 0.0001); I² = 86%						
Test for overall effect: Z = 8.73 (P < 0.00001)						
**2.8.12 Diarrhea（systemic adverse reactions）**						
Frenck 2021	68	1131	46	1129	1.9%	1.48 [1.02, 2.13]
Walter 2021	76	1514	38	747	1.9%	0.99 [0.68, 1.44]
**Subtotal (95% CI)**		**2645**		**1876**	**3.7%**	**1.21 [0.82, 1.80]**
Total events	144		84			
Heterogeneity: Tau² = 0.04; Chi² = 2.24, df = 1 (P = 0.13); I² = 55%						
Test for overall effect: Z = 0.95 (P = 0.34)						
**2.8.13 Irritability/Crying（systemic adverse reactions）**						
Anderson 2022	1115	1739	307	525	2.2%	1.10 [1.01, 1.19]
**Subtotal (95% CI)**		**1739**		**525**	**2.2%**	**1.10 [1.01, 1.19]**
Total events	1115		307			
Heterogeneity: Not applicable						
Test for overall effect: Z = 2.25 (P = 0.02)						
**2.8.14 Sleepiness (systemic adverse reactions)**						
Anderson 2022	600	1739	175	525	2.1%	1.04 [0.90, 1.19]
**Subtotal (95% CI)**		**1739**		**525**	**2.1%**	**1.04 [0.90, 1.19]**
Total events	600		175			
Heterogeneity: Not applicable						
Test for overall effect: Z = 0.49 (P = 0.62)						
**2.8.15 Loss of Appetite（systemic adverse reactions）**						
Anderson 2022	548	1739	132	525	2.1%	1.25 [1.06, 1.48]
**Subtotal (95% CI)**		**1739**		**525**	**2.1%**	**1.25 [1.06, 1.48]**
Total events	548		132			
Heterogeneity: Not applicable						
Test for overall effect: Z = 2.71 (P = 0.007)						
**Total (95% CI)**		**119315**		**52755**	**100.0%**	**3.13 [2.73, 3.59]**
Total events	37911		6359			
Heterogeneity: Tau² = 0.22; Chi² = 1739.92, df = 51 (P < 0.00001); I² = 97%						
Test for overall effect: Z = 16.43 (P < 0.00001)						
Test for subgroup differences: Chi² = 318.16, df = 14 (P < 0.00001), I² = 95.6%						

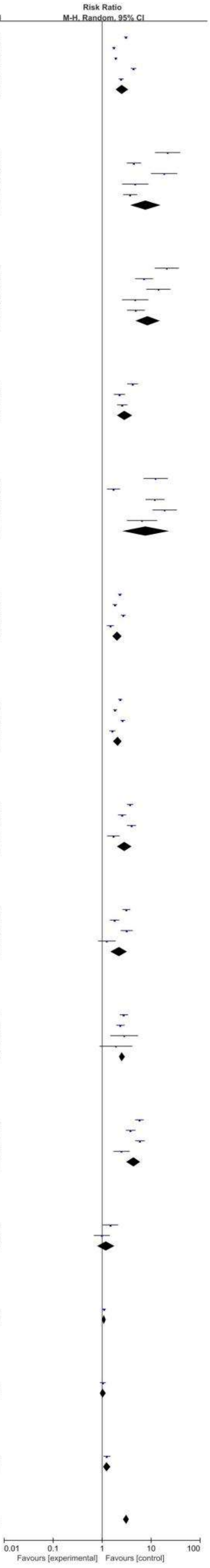

Figure S6. Specific adverse reactions in the inactivated vaccine group versus the control group:

A) After dose 1

Study or Subgroup	Experimental Events	Experimental Total	Control Events	Control Total	Weight	Risk Ratio M-H, Fixed, 95% CI
**2.9.1 Local pain（local adverse reactions）**						
Han 2021	40	436	0	114	1.7%	21.32 [1.32, 344.03]
Xia 2021	32	755	0	252	1.6%	21.75 [1.34, 353.95]
**Subtotal (95% CI)**		**1191**		**366**	**3.3%**	**21.53 [3.00, 154.35]**
Total events	72		0			
Heterogeneity: Chi² = 0.00, df = 1 (P = 0.99); I² = 0%						
Test for overall effect: Z = 3.05 (P = 0.002)						
**2.9.2 Erythema / Redness（local adverse reactions）**						
Xia 2021	4	755	1	252	3.2%	1.34 [0.15, 11.89]
**Subtotal (95% CI)**		**755**		**252**	**3.2%**	**1.34 [0.15, 11.89]**
Total events	4		1			
Heterogeneity: Not applicable						
Test for overall effect: Z = 0.26 (P = 0.80)						
**2.9.3 Swelling / Hardness (local adverse reavtions)**						
Han 2021	4	436	1	114	3.4%	1.05 [0.12, 9.27]
Xia 2021	0	755	1	252	4.8%	0.11 [0.00, 2.73]
**Subtotal (95% CI)**		**1191**		**366**	**8.2%**	**0.50 [0.10, 2.42]**
Total events	4		2			
Heterogeneity: Chi² = 1.29, df = 1 (P = 0.26); I² = 22%						
Test for overall effect: Z = 0.86 (P = 0.39)						
**2.9.5 Fever (systemic adverse reactions)**						
Han 2021	16	436	4	114	13.5%	1.05 [0.36, 3.07]
Xia 2021	43	755	8	252	25.5%	1.79 [0.86, 3.76]
**Subtotal (95% CI)**		**1191**		**366**	**39.0%**	**1.54 [0.84, 2.82]**
Total events	59		12			
Heterogeneity: Chi² = 0.66, df = 1 (P = 0.42); I² = 0%						
Test for overall effect: Z = 1.38 (P = 0.17)						
**2.9.6 Headache (systemic adverse reactions)**						
Han 2021	6	436	3	114	10.1%	0.52 [0.13, 2.06]
Xia 2021	6	755	0	252	1.6%	4.35 [0.25, 76.95]
**Subtotal (95% CI)**		**1191**		**366**	**11.7%**	**1.04 [0.33, 3.32]**
Total events	12		3			
Heterogeneity: Chi² = 1.93, df = 1 (P = 0.17); I² = 48%						
Test for overall effect: Z = 0.07 (P = 0.94)						
**2.9.7 Fatigue (systemic adverse reactions)**						
Han 2021	2	436	1	114	3.4%	0.52 [0.05, 5.72]
Xia 2021	3	755	0	252	1.6%	2.34 [0.12, 45.20]
**Subtotal (95% CI)**		**1191**		**366**	**5.0%**	**1.11 [0.19, 6.54]**
Total events	5		1			
Heterogeneity: Chi² = 0.62, df = 1 (P = 0.43); I² = 0%						
Test for overall effect: Z = 0.11 (P = 0.91)						
**2.9.8 Myalgia (systemic adverse reaction)**						
Han 2021	3	436	0	114	1.7%	1.84 [0.10, 35.41]
Xia 2021	3	755	0	252	1.6%	2.34 [0.12, 45.20]
**Subtotal (95% CI)**		**1191**		**366**	**3.3%**	**2.09 [0.26, 16.84]**
Total events	6		0			
Heterogeneity: Chi² = 0.01, df = 1 (P = 0.91); I² = 0%						
Test for overall effect: Z = 0.69 (P = 0.49)						
**2.9.10 Nausea / Vomiting (systemic adverse reactions)**						
Han 2021	4	436	2	114	6.7%	0.52 [0.10, 2.82]
**Subtotal (95% CI)**		**436**		**114**	**6.7%**	**0.52 [0.10, 2.82]**
Total events	4		2			
Heterogeneity: Not applicable						
Test for overall effect: Z = 0.75 (P = 0.45)						
**2.9.12 Diarrhea (systemic adverse reactions)**						
Han 2021	3	436	3	114	10.1%	0.26 [0.05, 1.28]
Xia 2021	6	755	3	252	9.6%	0.67 [0.17, 2.65]
**Subtotal (95% CI)**		**1191**		**366**	**19.7%**	**0.46 [0.17, 1.27]**
Total events	9		6			
Heterogeneity: Chi² = 0.77, df = 1 (P = 0.38); I² = 0%						
Test for overall effect: Z = 1.50 (P = 0.13)						
**Total (95% CI)**		**9528**		**2928**	**100.0%**	**1.76 [1.20, 2.57]**
Total events	175		27			
Heterogeneity: Chi² = 24.13, df = 15 (P = 0.06); I² = 38%						
Test for overall effect: Z = 2.92 (P = 0.004)						
Test for subgroup differences: Chi² = 14.77, df = 8 (P = 0.06), I² = 45.9%						

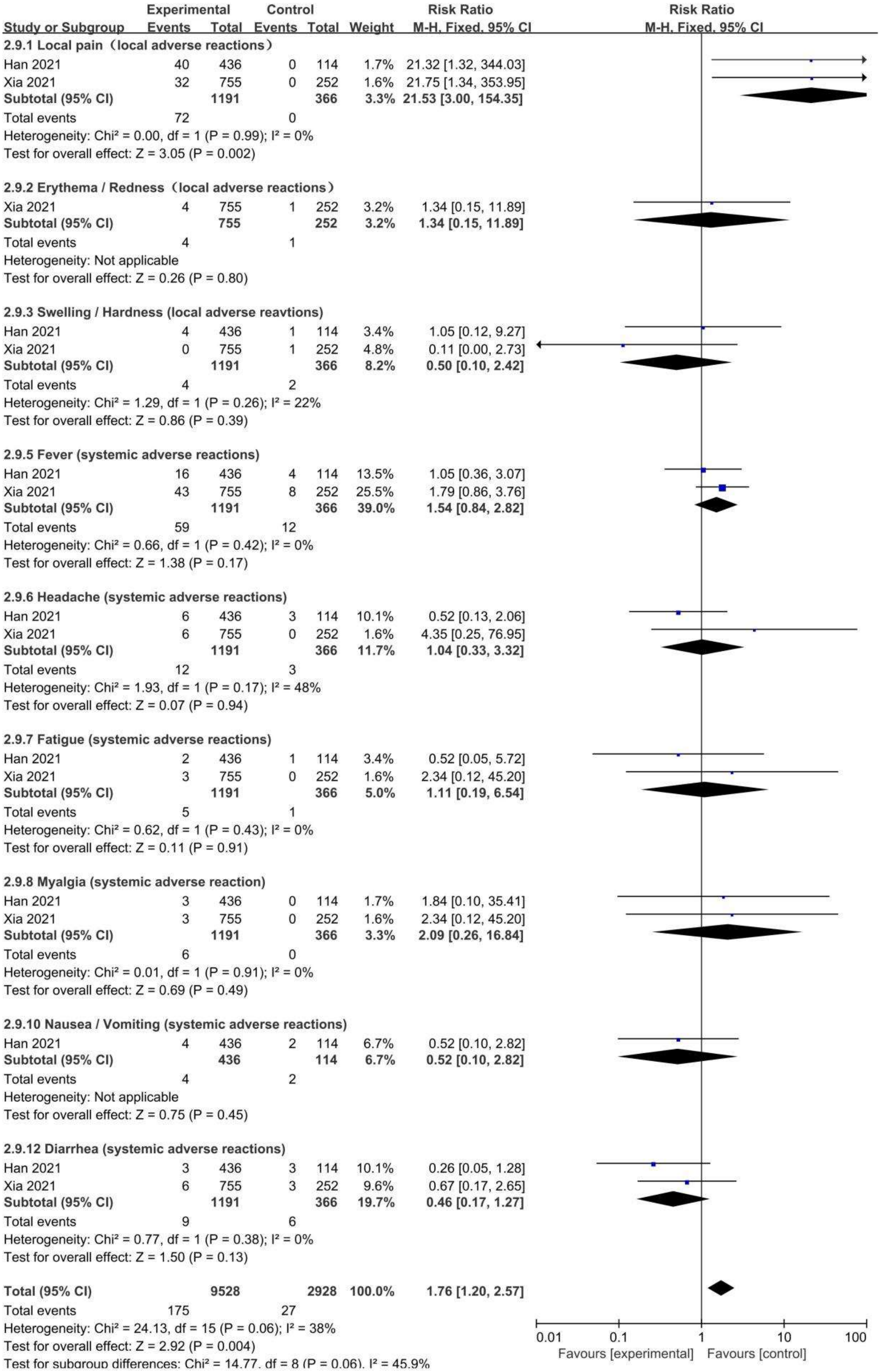

B) After dose 2

Study or Subgroup	Experimental Events	Experimental Total	Control Events	Control Total	Weight	Risk Ratio M-H, Fixed, 95% CI
**2.10.1 Local pain (local adverse reactions)**						
Han 2021	45	425	2	111	12.9%	5.88 [1.45, 23.85]
Xia 2021	16	751	0	248	3.0%	10.93 [0.66, 181.47]
**Subtotal (95% CI)**		**1176**		**359**	**15.9%**	**6.84 [1.96, 23.90]**
Total events	61		2			
Heterogeneity: Chi² = 0.15, df = 1 (P = 0.70); I² = 0%						
Test for overall effect: Z = 3.01 (P = 0.003)						
**2.10.2 Erythema / Redness (local adverse reactions)**						
Han 2021	1	425	0	111	3.2%	0.79 [0.03, 19.23]
Xia 2021	8	751	2	248	12.2%	1.32 [0.28, 6.18]
**Subtotal (95% CI)**		**1176**		**359**	**15.4%**	**1.21 [0.30, 4.83]**
Total events	9		2			
Heterogeneity: Chi² = 0.08, df = 1 (P = 0.78); I² = 0%						
Test for overall effect: Z = 0.27 (P = 0.79)						
**2.10.3 Swelling / Hardness (local adverse reactions)**						
Han 2021	6	425	0	111	3.2%	3.42 [0.19, 60.21]
Xia 2021	1	751	0	248	3.0%	0.99 [0.04, 24.31]
**Subtotal (95% CI)**		**1176**		**359**	**6.3%**	**2.24 [0.28, 18.17]**
Total events	7		0			
Heterogeneity: Chi² = 0.33, df = 1 (P = 0.56); I² = 0%						
Test for overall effect: Z = 0.75 (P = 0.45)						
**2.10.4 Fever (systemic adverse reactions)**						
Han 2021	5	425	1	111	6.4%	1.31 [0.15, 11.06]
Xia 2021	21	751	4	248	24.4%	1.73 [0.60, 5.00]
**Subtotal (95% CI)**		**1176**		**359**	**30.8%**	**1.64 [0.64, 4.24]**
Total events	26		5			
Heterogeneity: Chi² = 0.05, df = 1 (P = 0.82); I² = 0%						
Test for overall effect: Z = 1.03 (P = 0.30)						
**2.10.5 Headache (systemic adverse reactions)**						
Han 2021	5	425	0	111	3.2%	2.89 [0.16, 51.91]
Xia 2021	1	751	1	248	6.1%	0.33 [0.02, 5.26]
**Subtotal (95% CI)**		**1176**		**359**	**9.3%**	**1.21 [0.20, 7.38]**
Total events	6		1			
Heterogeneity: Chi² = 1.20, df = 1 (P = 0.27); I² = 16%						
Test for overall effect: Z = 0.21 (P = 0.83)						
**2.10.6 Fatigue (systemic adverse reactions)**						
Han 2021	1	425	0	111	3.2%	0.79 [0.03, 19.23]
Xia 2021	0	751	0	248		Not estimable
**Subtotal (95% CI)**		**1176**		**359**	**3.2%**	**0.79 [0.03, 19.23]**
Total events	1		0			
Heterogeneity: Not applicable						
Test for overall effect: Z = 0.15 (P = 0.88)						
**2.10.7 Myalgia (systemic adverse reactions)**						
Han 2021	1	425	0	111	3.2%	0.79 [0.03, 19.23]
Xia 2021	1	751	0	248	3.0%	0.99 [0.04, 24.31]
**Subtotal (95% CI)**		**1176**		**359**	**6.3%**	**0.89 [0.09, 8.49]**
Total events	2		0			
Heterogeneity: Chi² = 0.01, df = 1 (P = 0.92); I² = 0%						
Test for overall effect: Z = 0.10 (P = 0.92)						
**2.10.8 Nausea / Vomiting (systemic adverse reactions)**						
Han 2021	3	425	1	111	6.4%	0.78 [0.08, 7.46]
**Subtotal (95% CI)**		**425**		**111**	**6.4%**	**0.78 [0.08, 7.46]**
Total events	3		1			
Heterogeneity: Not applicable						
Test for overall effect: Z = 0.21 (P = 0.83)						
**2.10.9 Diarrhea (systemic adverse reactions)**						
Han 2021	1	425	1	111	6.4%	0.26 [0.02, 4.14]
**Subtotal (95% CI)**		**425**		**111**	**6.4%**	**0.26 [0.02, 4.14]**
Total events	1		1			
Heterogeneity: Not applicable						
Test for overall effect: Z = 0.95 (P = 0.34)						
**Total (95% CI)**		**9082**		**2735**	**100.0%**	**2.18 [1.30, 3.67]**
Total events	116		12			
Heterogeneity: Chi² = 10.61, df = 14 (P = 0.72); I² = 0%						
Test for overall effect: Z = 2.94 (P = 0.003)						
Test for subgroup differences: Chi² = 7.96, df = 8 (P = 0.44), I² = 0%						

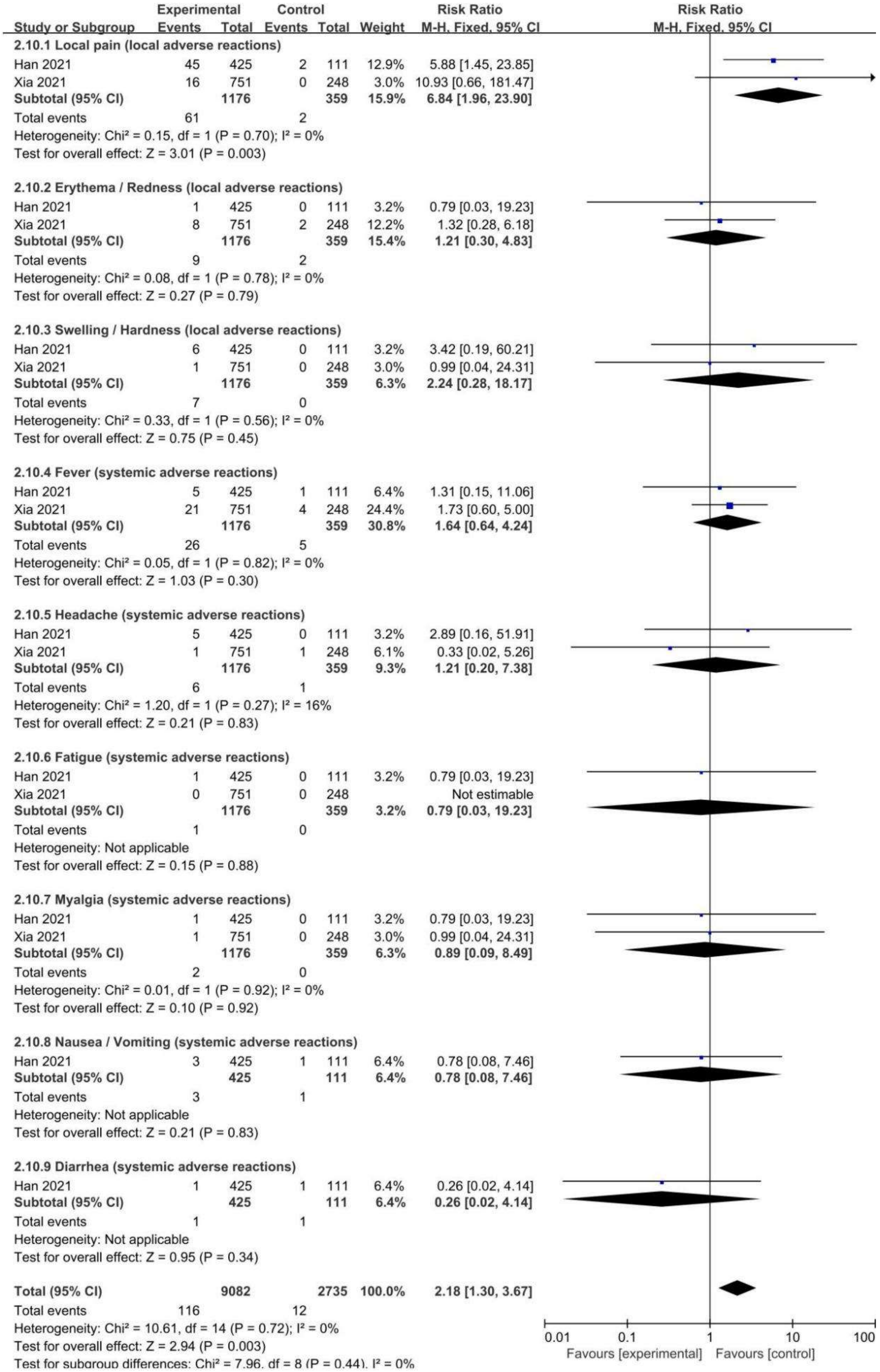

Figure S7. Specific adverse reactions in the subunit vaccine group versus the control group:

A) After dose 1

	Experimental		Control			Risk Ratio
Study or Subgroup	Events	Total	Events	Total	Weight	M-H, Fixed, 95% CI
**2.11.1 Local pain (local adverse reactions)**						
Liu 2022	205	341	12	58	25.3%	2.91 [1.74, 4.84]
**Subtotal (95% CI)**		**341**		**58**	**25.3%**	**2.91 [1.74, 4.84]**
Total events	205		12			
Heterogeneity: Not applicable						
Test for overall effect: Z = 4.09 (P < 0.0001)						
**2.11.2 Erythema / Redness (local adverse reactions)**						
Liu 2022	2	341	0	58	1.1%	0.86 [0.04, 17.74]
**Subtotal (95% CI)**		**341**		**58**	**1.1%**	**0.86 [0.04, 17.74]**
Total events	2		0			
Heterogeneity: Not applicable						
Test for overall effect: Z = 0.10 (P = 0.92)						
**2.11.3 Swelling / Hardness (loclal adverse reactions)**						
Liu 2022	7	341	0	58	1.1%	2.59 [0.15, 44.71]
**Subtotal (95% CI)**		**341**		**58**	**1.1%**	**2.59 [0.15, 44.71]**
Total events	7		0			
Heterogeneity: Not applicable						
Test for overall effect: Z = 0.65 (P = 0.51)						
**2.11.4 Fever (systemic adverse reactions)**						
Liu 2022	3	341	0	58	1.1%	1.21 [0.06, 23.08]
**Subtotal (95% CI)**		**341**		**58**	**1.1%**	**1.21 [0.06, 23.08]**
Total events	3		0			
Heterogeneity: Not applicable						
Test for overall effect: Z = 0.13 (P = 0.90)						
**2.11.5 Headache (systemic adverse reactions)**						
Liu 2022	49	341	9	58	18.9%	0.93 [0.48, 1.78]
**Subtotal (95% CI)**		**341**		**58**	**18.9%**	**0.93 [0.48, 1.78]**
Total events	49		9			
Heterogeneity: Not applicable						
Test for overall effect: Z = 0.23 (P = 0.82)						
**2.11.6 Fatigue (systemic adverse reactions)**						
Liu 2022	78	341	9	58	18.9%	1.47 [0.78, 2.77]
**Subtotal (95% CI)**		**341**		**58**	**18.9%**	**1.47 [0.78, 2.77]**
Total events	78		9			
Heterogeneity: Not applicable						
Test for overall effect: Z = 1.20 (P = 0.23)						
**2.11.7 Myalgia (systemic adverse reactions)**						
Liu 2022	66	341	10	58	21.1%	1.12 [0.61, 2.05]
**Subtotal (95% CI)**		**341**		**58**	**21.1%**	**1.12 [0.61, 2.05]**
Total events	66		10			
Heterogeneity: Not applicable						
Test for overall effect: Z = 0.38 (P = 0.71)						
**2.11.8 Nausea / Vomiting (systemic adverse reactions)**						
Liu 2022	17	341	2	58	4.2%	1.45 [0.34, 6.09]
**Subtotal (95% CI)**		**341**		**58**	**4.2%**	**1.45 [0.34, 6.09]**
Total events	17		2			
Heterogeneity: Not applicable						
Test for overall effect: Z = 0.50 (P = 0.62)						
**2.11.9 Diarrhea (systemic adverse reactions)**						
Liu 2022	34	341	4	58	8.4%	1.45 [0.53, 3.92]
**Subtotal (95% CI)**		**341**		**58**	**8.4%**	**1.45 [0.53, 3.92]**
Total events	34		4			
Heterogeneity: Not applicable						
Test for overall effect: Z = 0.72 (P = 0.47)						
**Total (95% CI)**		**3069**		**522**	**100.0%**	**1.66 [1.26, 2.17]**
Total events	461		46			
Heterogeneity: $Chi^2 = 9.83$, df = 8 (P = 0.28); $I^2 = 19\%$						
Test for overall effect: Z = 3.65 (P = 0.0003)						
Test for subgroup differences: $Chi^2 = 9.63$, df = 8 (P = 0.29), $I^2 = 16.9\%$						

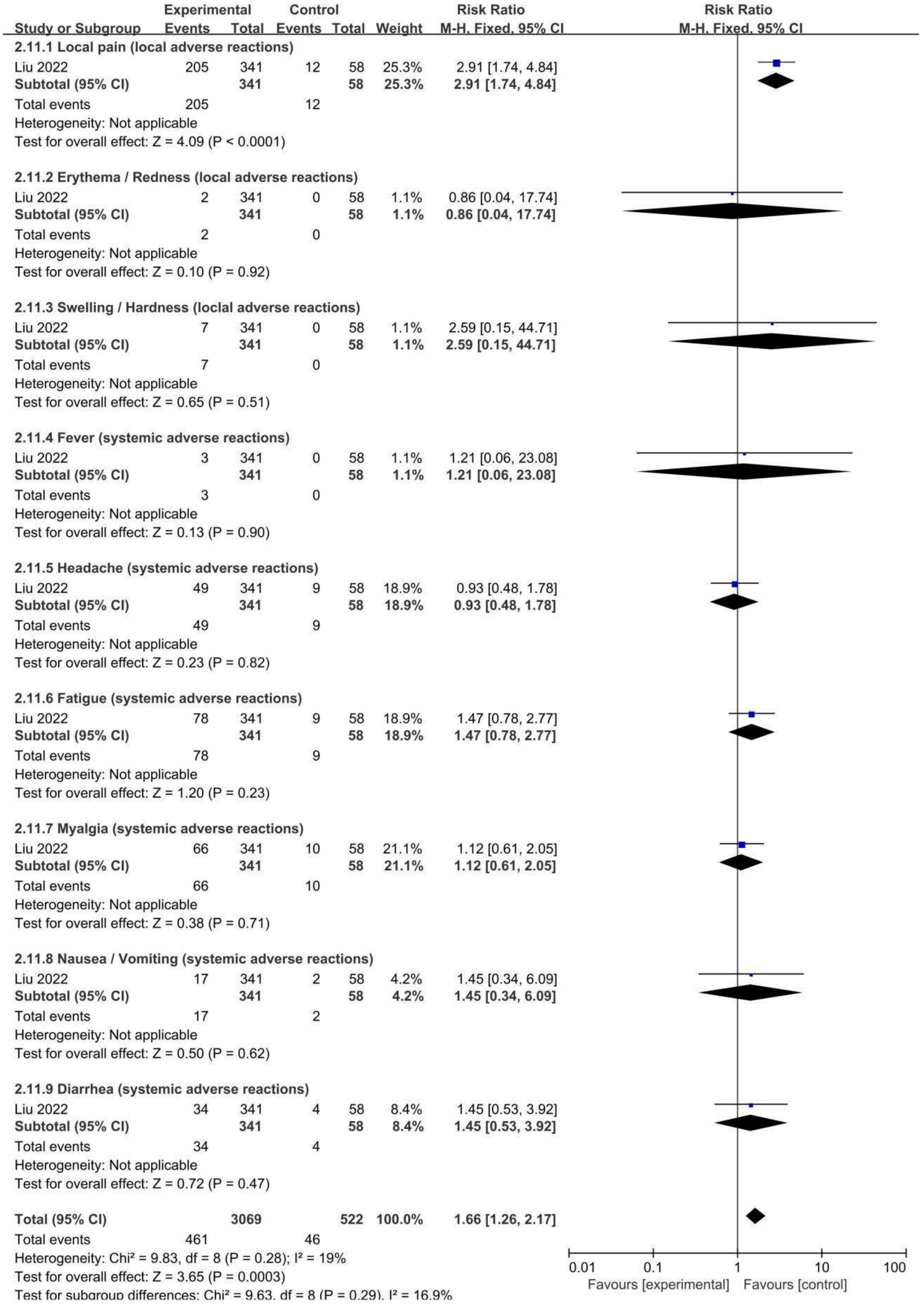

B) After dose 2

Study or Subgroup	Experimental Events	Experimental Total	Control Events	Control Total	Weight	Risk Ratio M-H, Fixed, 95% CI
**2.12.1 Local pain（local adverse reactions）**						
Liu 2022	162	340	14	58	40.0%	1.97 [1.23, 3.16]
**Subtotal (95% CI)**		**340**		**58**	**40.0%**	**1.97 [1.23, 3.16]**
Total events	162		14			
Heterogeneity: Not applicable						
Test for overall effect: Z = 2.84 (P = 0.005)						
**2.12.2 Erythema/Redness（local adverse reactions）**						
Liu 2022	2	340	0	58	1.4%	0.87 [0.04, 17.79]
**Subtotal (95% CI)**		**340**		**58**	**1.4%**	**0.87 [0.04, 17.79]**
Total events	2		0			
Heterogeneity: Not applicable						
Test for overall effect: Z = 0.09 (P = 0.93)						
**2.12.3 Swelling / Hardness（local adverse reactions）**						
Liu 2022	5	340	0	58	1.4%	1.90 [0.11, 33.97]
**Subtotal (95% CI)**		**340**		**58**	**1.4%**	**1.90 [0.11, 33.97]**
Total events	5		0			
Heterogeneity: Not applicable						
Test for overall effect: Z = 0.44 (P = 0.66)						
**2.12.4 Fever (systemic adverse reactions)**						
Liu 2022	1	340	0	58	1.4%	0.52 [0.02, 12.59]
**Subtotal (95% CI)**		**340**		**58**	**1.4%**	**0.52 [0.02, 12.59]**
Total events	1		0			
Heterogeneity: Not applicable						
Test for overall effect: Z = 0.40 (P = 0.69)						
**2.12.5 Headache (systemic adverse reactions)**						
Liu 2022	1	340	0	58	1.4%	0.52 [0.02, 12.59]
**Subtotal (95% CI)**		**340**		**58**	**1.4%**	**0.52 [0.02, 12.59]**
Total events	1		0			
Heterogeneity: Not applicable						
Test for overall effect: Z = 0.40 (P = 0.69)						
**2.12.6 Fatigue (systemic adverse reactions)**						
Liu 2022	63	340	8	58	22.9%	1.34 [0.68, 2.65]
**Subtotal (95% CI)**		**340**		**58**	**22.9%**	**1.34 [0.68, 2.65]**
Total events	63		8			
Heterogeneity: Not applicable						
Test for overall effect: Z = 0.85 (P = 0.40)						
**2.12.7 Myalgia (systemic adverse reactions)**						
Liu 2022	27	340	6	58	17.1%	0.77 [0.33, 1.78]
**Subtotal (95% CI)**		**340**		**58**	**17.1%**	**0.77 [0.33, 1.78]**
Total events	27		6			
Heterogeneity: Not applicable						
Test for overall effect: Z = 0.62 (P = 0.54)						
**2.12.8 Nausea / vomiting (systemic adverse reactions)**						
Liu 2022	6	340	3	58	8.6%	0.34 [0.09, 1.33]
**Subtotal (95% CI)**		**340**		**58**	**8.6%**	**0.34 [0.09, 1.33]**
Total events	6		3			
Heterogeneity: Not applicable						
Test for overall effect: Z = 1.55 (P = 0.12)						
**2.12.9 Diarrhea (systemic adverse reactions)**						
Liu 2022	18	340	2	58	5.7%	1.54 [0.37, 6.44]
**Subtotal (95% CI)**		**340**		**58**	**5.7%**	**1.54 [0.37, 6.44]**
Total events	18		2			
Heterogeneity: Not applicable						
Test for overall effect: Z = 0.59 (P = 0.56)						
**Total (95% CI)**		**3060**		**522**	**100.0%**	**1.40 [1.02, 1.92]**
Total events	285		33			
Heterogeneity: $Chi^2 = 9.09$, df = 8 (P = 0.33); $I^2 = 12\%$						
Test for overall effect: Z = 2.08 (P = 0.04)						
Test for subgroup differences: $Chi^2 = 9.05$, df = 8 (P = 0.34), $I^2 = 11.6\%$						

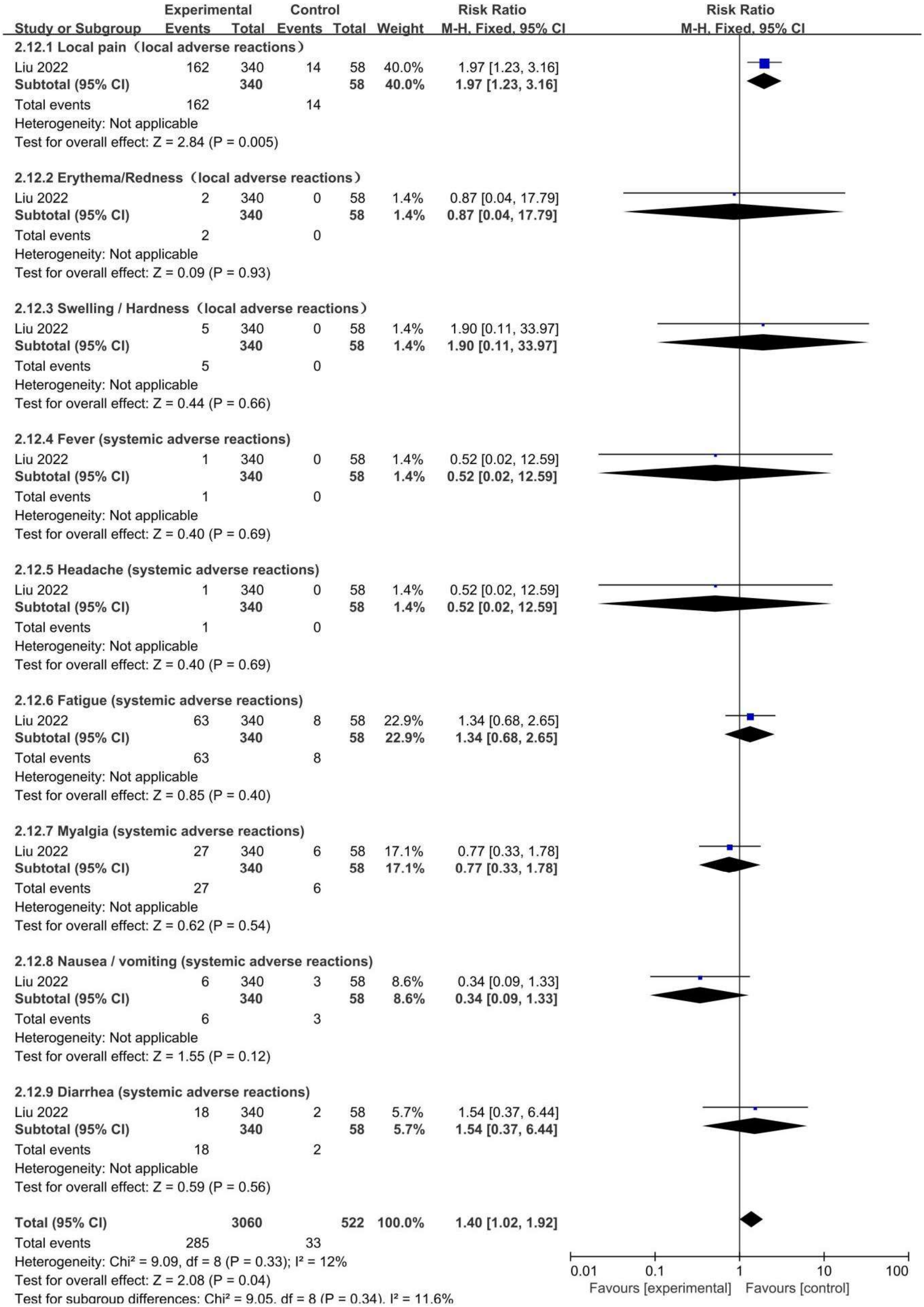

Figure S8. Specific adverse reactions in the adenovirus vector vaccine group versus the control group:

A) After dose 1

Study or Subgroup	Experimental Events	Experimental Total	Control Events	Control Total	Weight	Risk Ratio M-H, Fixed, 95% CI
**2.14.1 Local pain（local adverse reactions）**						
Zhu 2021	34	100	3	50	30.0%	5.67 [1.83, 17.55]
**Subtotal (95% CI)**		**100**		**50**	**30.0%**	**5.67 [1.83, 17.55]**
Total events	34		3			
Heterogeneity: Not applicable						
Test for overall effect: Z = 3.01 (P = 0.003)						
**2.14.2 Erythema/Redness（local adverse reactions）**						
Zhu 2021	2	100	0	50	5.0%	2.52 [0.12, 51.61]
**Subtotal (95% CI)**		**100**		**50**	**5.0%**	**2.52 [0.12, 51.61]**
Total events	2		0			
Heterogeneity: Not applicable						
Test for overall effect: Z = 0.60 (P = 0.55)						
**2.14.3 Swelling / Hardness（local adverse reactions）**						
Zhu 2021	4	100	0	50	5.0%	4.54 [0.25, 82.78]
**Subtotal (95% CI)**		**100**		**50**	**5.0%**	**4.54 [0.25, 82.78]**
Total events	4		0			
Heterogeneity: Not applicable						
Test for overall effect: Z = 1.02 (P = 0.31)						
**2.14.4 Fever (systemic adverse reactions)**						
Zhu 2021	28	100	2	50	20.0%	7.00 [1.74, 28.21]
**Subtotal (95% CI)**		**100**		**50**	**20.0%**	**7.00 [1.74, 28.21]**
Total events	28		2			
Heterogeneity: Not applicable						
Test for overall effect: Z = 2.74 (P = 0.006)						
**2.14.5 Headache (systemic adverse reactions)**						
Zhu 2021	14	100	0	50	5.0%	14.64 [0.89, 240.57]
**Subtotal (95% CI)**		**100**		**50**	**5.0%**	**14.64 [0.89, 240.57]**
Total events	14		0			
Heterogeneity: Not applicable						
Test for overall effect: Z = 1.88 (P = 0.06)						
**2.14.6 Fatigue (systemic adverse reactions)**						
Zhu 2021	9	100	2	50	20.0%	2.25 [0.51, 10.02]
**Subtotal (95% CI)**		**100**		**50**	**20.0%**	**2.25 [0.51, 10.02]**
Total events	9		2			
Heterogeneity: Not applicable						
Test for overall effect: Z = 1.06 (P = 0.29)						
**2.14.7 Myalgia (systemic adverse reactions)**						
Zhu 2021	4	100	0	50	5.0%	4.54 [0.25, 82.78]
**Subtotal (95% CI)**		**100**		**50**	**5.0%**	**4.54 [0.25, 82.78]**
Total events	4		0			
Heterogeneity: Not applicable						
Test for overall effect: Z = 1.02 (P = 0.31)						
**2.14.8 Arthralgia (systemic adverse reactions)**						
Zhu 2021	1	100	0	50	5.0%	1.51 [0.06, 36.53]
**Subtotal (95% CI)**		**100**		**50**	**5.0%**	**1.51 [0.06, 36.53]**
Total events	1		0			
Heterogeneity: Not applicable						
Test for overall effect: Z = 0.26 (P = 0.80)						
**2.14.9 Nausea / vomiting (systemic adverse reactions)**						
Zhu 2021	6	100	0	50	5.0%	6.56 [0.38, 114.24]
**Subtotal (95% CI)**		**100**		**50**	**5.0%**	**6.56 [0.38, 114.24]**
Total events	6		0			
Heterogeneity: Not applicable						
Test for overall effect: Z = 1.29 (P = 0.20)						
**Total (95% CI)**		**900**		**450**	**100.0%**	**5.27 [2.80, 9.91]**
Total events	102		7			
Heterogeneity: Chi² = 2.79, df = 8 (P = 0.95); I² = 0%						
Test for overall effect: Z = 5.15 (P < 0.00001)						
Test for subgroup differences: Chi² = 2.69, df = 8 (P = 0.95), I² = 0%						

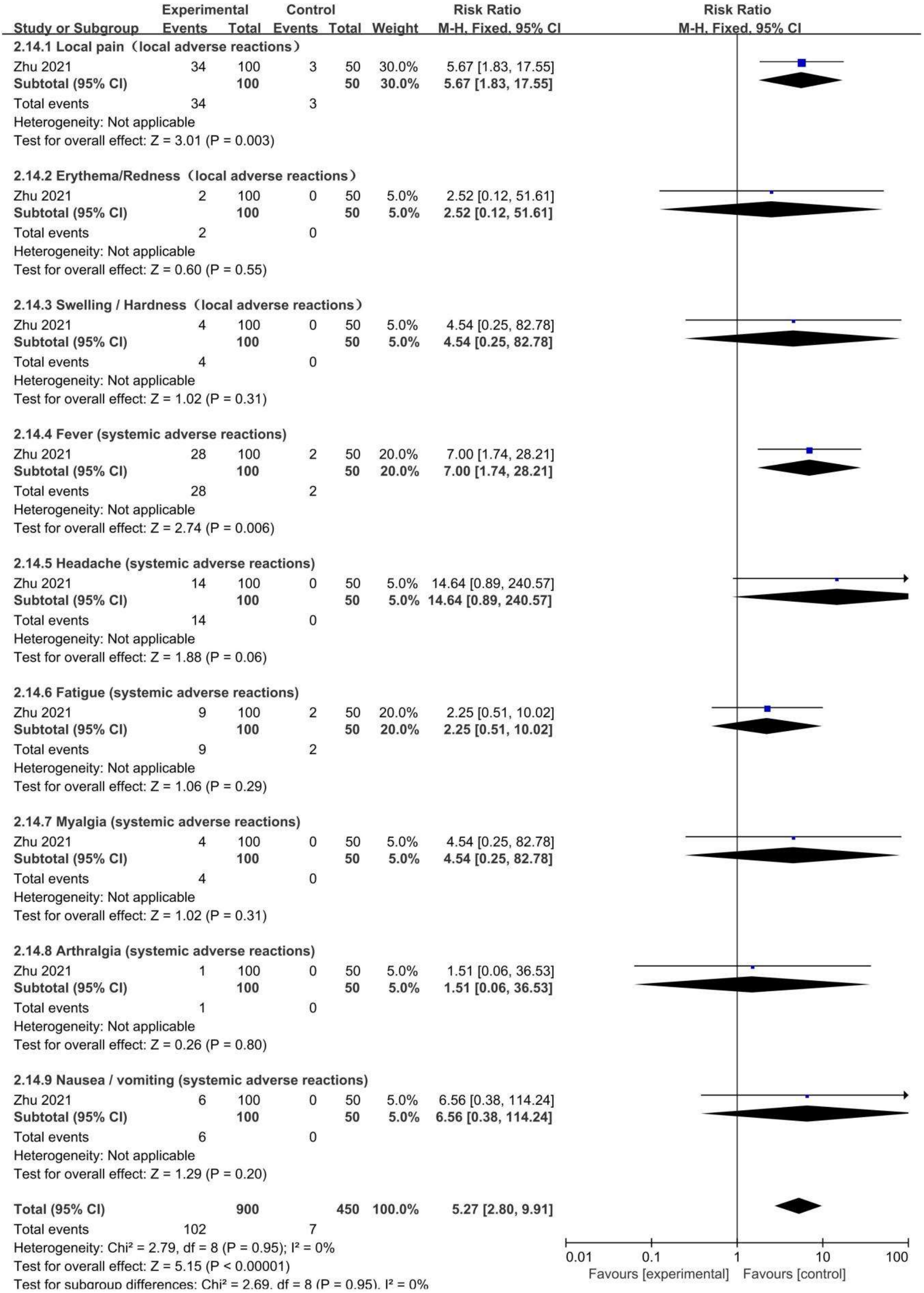

B) Afte dose 2

Study or Subgroup	Experimental Events	Experimental Total	Control Events	Control Total	Weight	Risk Ratio M-H, Fixed, 95% CI
**2.15.1 Local pain（local adverse reactions）**						
Zhu 2021	14	100	0	50	11.1%	14.64 [0.89, 240.57]
**Subtotal (95% CI)**		**100**		**50**	**11.1%**	**14.64 [0.89, 240.57]**
Total events	14		0			
Heterogeneity: Not applicable						
Test for overall effect: Z = 1.88 (P = 0.06)						
**2.15.2 Erythema/Redness（local adverse reactions）**						
Zhu 2021	0	100	0	50		Not estimable
**Subtotal (95% CI)**		**100**		**50**		**Not estimable**
Total events	0		0			
Heterogeneity: Not applicable						
Test for overall effect: Not applicable						
**2.15.3 Swelling / Hardness（local adverse reactions）**						
Zhu 2021	4	100	0	50	11.1%	4.54 [0.25, 82.78]
**Subtotal (95% CI)**		**100**		**50**	**11.1%**	**4.54 [0.25, 82.78]**
Total events	4		0			
Heterogeneity: Not applicable						
Test for overall effect: Z = 1.02 (P = 0.31)						
**2.15.4 Fever (systemic adverse reactions)**						
Zhu 2021	13	100	1	50	22.3%	6.50 [0.87, 48.29]
**Subtotal (95% CI)**		**100**		**50**	**22.3%**	**6.50 [0.87, 48.29]**
Total events	13		1			
Heterogeneity: Not applicable						
Test for overall effect: Z = 1.83 (P = 0.07)						
**2.15.5 Headache (systemic adverse reactions)**						
Zhu 2021	8	100	0	50	11.1%	8.58 [0.51, 145.79]
**Subtotal (95% CI)**		**100**		**50**	**11.1%**	**8.58 [0.51, 145.79]**
Total events	8		0			
Heterogeneity: Not applicable						
Test for overall effect: Z = 1.49 (P = 0.14)						
**2.15.6 Fatigue (systemic adverse reactions)**						
Zhu 2021	6	100	0	50	11.1%	6.56 [0.38, 114.24]
**Subtotal (95% CI)**		**100**		**50**	**11.1%**	**6.56 [0.38, 114.24]**
Total events	6		0			
Heterogeneity: Not applicable						
Test for overall effect: Z = 1.29 (P = 0.20)						
**2.15.7 Myalgia (systemic adverse reactions)**						
Zhu 2021	1	100	0	50	11.1%	1.51 [0.06, 36.53]
**Subtotal (95% CI)**		**100**		**50**	**11.1%**	**1.51 [0.06, 36.53]**
Total events	1		0			
Heterogeneity: Not applicable						
Test for overall effect: Z = 0.26 (P = 0.80)						
**2.15.8 Arthralgia (systemic adverse reactions)**						
Zhu 2021	1	100	0	50	11.1%	1.51 [0.06, 36.53]
**Subtotal (95% CI)**		**100**		**50**	**11.1%**	**1.51 [0.06, 36.53]**
Total events	1		0			
Heterogeneity: Not applicable						
Test for overall effect: Z = 0.26 (P = 0.80)						
**2.15.9 Nausea / vomiting (systemic adverse reactions)**						
Zhu 2021	5	100	0	50	11.1%	5.55 [0.31, 98.50]
**Subtotal (95% CI)**		**100**		**50**	**11.1%**	**5.55 [0.31, 98.50]**
Total events	5		0			
Heterogeneity: Not applicable						
Test for overall effect: Z = 1.17 (P = 0.24)						
**Total (95% CI)**		**900**		**450**	**100.0%**	**6.21 [2.40, 16.11]**
Total events	52		1			
Heterogeneity: Chi² = 1.97, df = 7 (P = 0.96); I² = 0%						
Test for overall effect: Z = 3.76 (P = 0.0002)						
Test for subgroup differences: Chi² = 1.88, df = 7 (P = 0.97), I² = 0%						

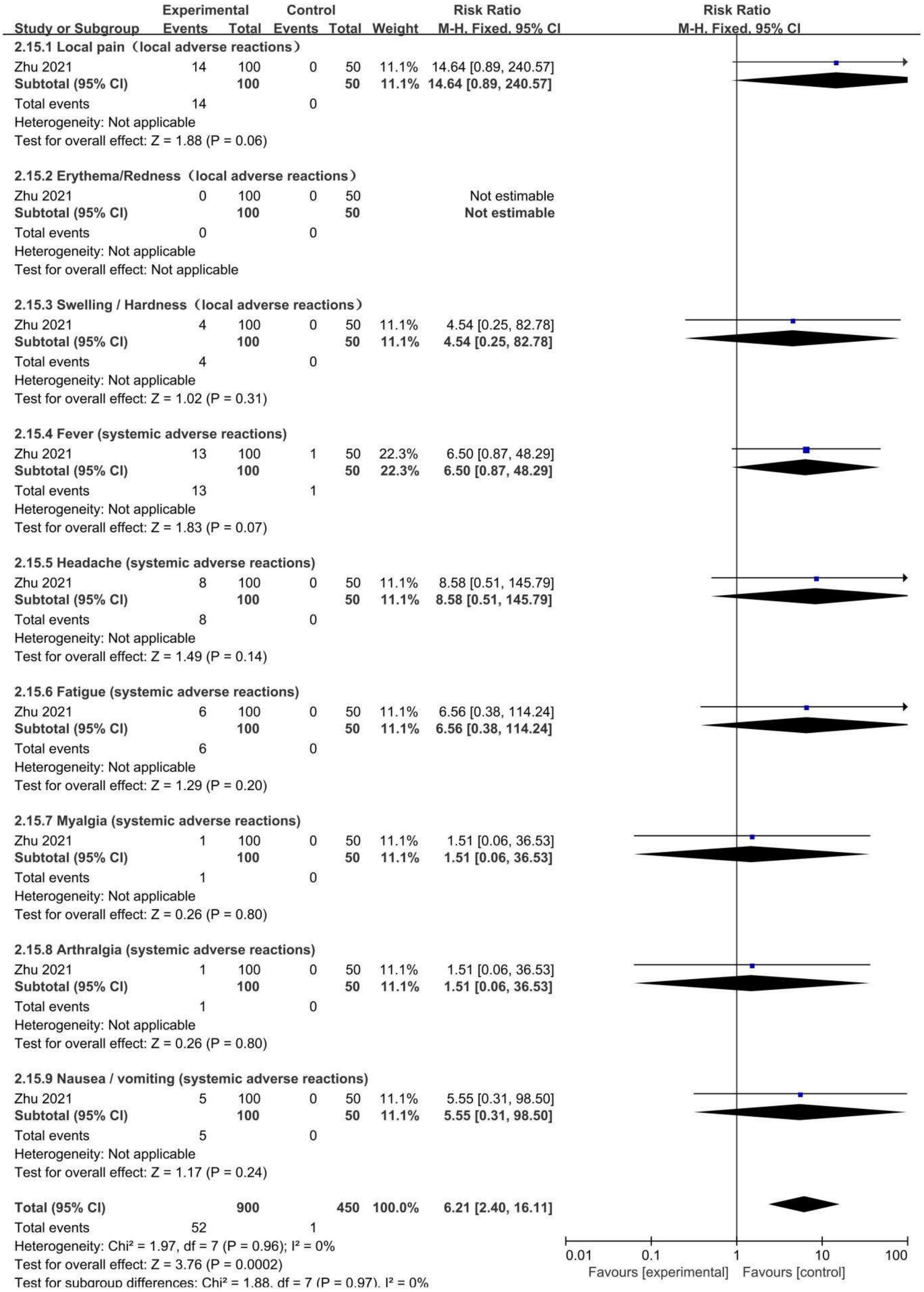

Figure S9. Adverse reactions in the mRNA vaccine group of different ages versus the control group:

A) Local pain after dose 1

Study or Subgroup	Experimental Events	Experimental Total	Control Events	Control Total	Weight	Risk Ratio M-H, Random, 95% CI
**3.1.1 ⩾12 years old**						
Ali 2021	2310	2482	431	1238	20.1%	2.67 [2.48, 2.89]
Frenck 2021	972	1131	260	1129	19.8%	3.73 [3.35, 4.16]
**Subtotal (95% CI)**		**3613**		**2367**	**39.9%**	**3.15 [2.27, 4.37]**
Total events	3282		691			
Heterogeneity: Tau² = 0.05; Chi² = 23.97, df = 1 (P < 0.00001); I² = 96%						
Test for overall effect: Z = 6.88 (P < 0.00001)						
**3.1.2 ＜12 years old**						
Anderson 2022	2552	4916	557	1552	20.1%	1.45 [1.35, 1.55]
Buddy Creech 2022	3132	3382	465	993	20.2%	1.98 [1.85, 2.11]
Walter 2021	1124	1518	233	750	19.8%	2.38 [2.13, 2.66]
**Subtotal (95% CI)**		**9816**		**3295**	**60.1%**	**1.89 [1.44, 2.48]**
Total events	6808		1255			
Heterogeneity: Tau² = 0.06; Chi² = 67.99, df = 2 (P < 0.00001); I² = 97%						
Test for overall effect: Z = 4.61 (P < 0.00001)						
**Total (95% CI)**		**13429**		**5662**	**100.0%**	**2.32 [1.72, 3.13]**
Total events	10090		1946			
Heterogeneity: Tau² = 0.11; Chi² = 256.03, df = 4 (P < 0.00001); I² = 98%						
Test for overall effect: Z = 5.55 (P < 0.00001)						
Test for subgroup differences: Chi² = 5.55, df = 1 (P = 0.02), I² = 82.0%						

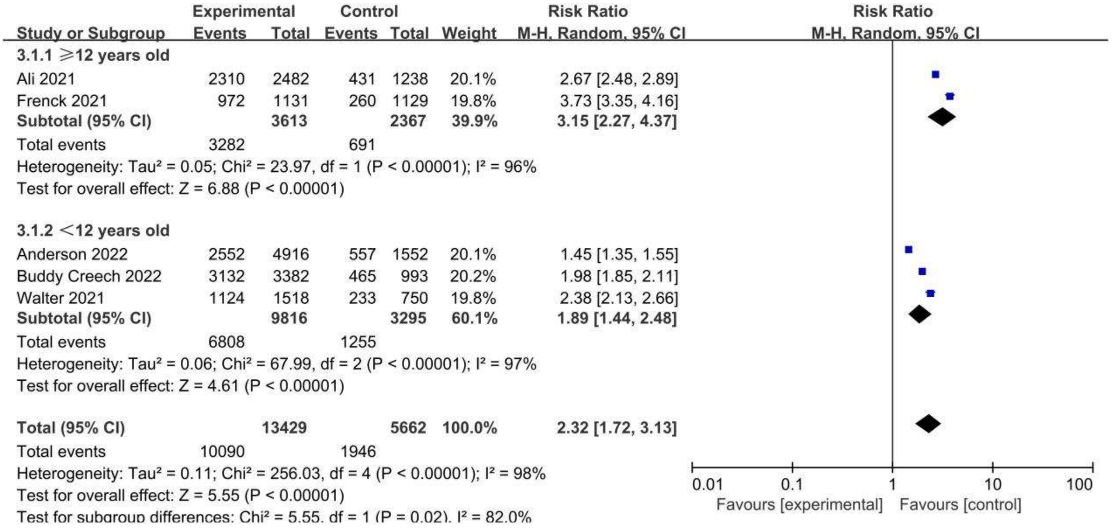

B) Local pain after dose 2

Study or Subgroup	Experimental Events	Experimental Total	Control Events	Control Total	Weight	Risk Ratio M-H, Random, 95% CI
**3.1.1 ⩾12 years old**						
Ali 2021	2290	2478	370	1220	20.1%	3.05 [2.80, 3.32]
Frenck 2021	894	1131	204	1129	19.7%	4.37 [3.85, 4.97]
**Subtotal (95% CI)**		**3609**		**2349**	**39.8%**	**3.64 [2.55, 5.19]**
Total events	3184		574			
Heterogeneity: Tau² = 0.06; Chi² = 21.24, df = 1 (P < 0.00001); I² = 95%						
Test for overall effect: Z = 7.14 (P < 0.00001)						
**3.1.2 ＜12 years old**						
Anderson 2022	2949	4753	530	1485	20.2%	1.74 [1.62, 1.87]
Buddy Creech 2022	3182	3367	480	969	20.2%	1.91 [1.79, 2.03]
Walter 2021	1075	1514	217	747	19.8%	2.44 [2.18, 2.75]
**Subtotal (95% CI)**		**9634**		**3201**	**60.2%**	**1.99 [1.70, 2.34]**
Total events	7206		1227			
Heterogeneity: Tau² = 0.02; Chi² = 23.84, df = 2 (P < 0.00001); I² = 92%						
Test for overall effect: Z = 8.41 (P < 0.00001)						
**Total (95% CI)**		**13243**		**5550**	**100.0%**	**2.54 [1.89, 3.42]**
Total events	10390		1801			
Heterogeneity: Tau² = 0.11; Chi² = 233.39, df = 4 (P < 0.00001); I² = 98%						
Test for overall effect: Z = 6.17 (P < 0.00001)						
Test for subgroup differences: Chi² = 9.16, df = 1 (P = 0.002), I² = 89.1%						

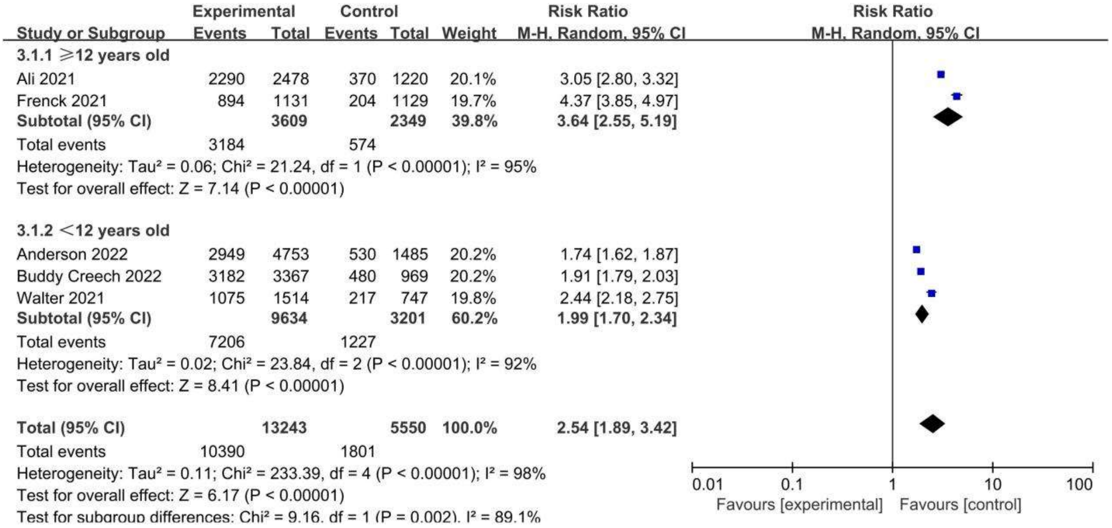

C) Erythema or Redness after dose 1

Study or Subgroup	Experimental Events	Experimental Total	Control Events	Control Total	Weight	Risk Ratio M-H, Random, 95% CI
**3.2.1 ≥12 years old**						
Ali 2021	334	2482	8	1238	18.4%	20.82 [10.36, 41.85]
Frenck 2021	68	1131	12	1129	19.2%	5.66 [3.08, 10.39]
**Subtotal (95% CI)**		**3613**		**2367**	**37.6%**	**10.74 [2.72, 42.37]**
Total events	402		20			
Heterogeneity: Tau² = 0.87; Chi² = 8.79, df = 1 (P = 0.003); I² = 89%						
Test for overall effect: Z = 3.39 (P = 0.0007)						
**3.2.2 <12 years old**						
Anderson 2022	329	4917	38	1552	21.3%	2.73 [1.96, 3.80]
Buddy Creech 2022	393	3382	13	993	19.7%	8.88 [5.13, 15.35]
Walter 2021	228	1518	45	750	21.4%	2.50 [1.84, 3.40]
**Subtotal (95% CI)**		**9817**		**3295**	**62.4%**	**3.78 [1.96, 7.29]**
Total events	950		96			
Heterogeneity: Tau² = 0.29; Chi² = 17.95, df = 2 (P = 0.0001); I² = 89%						
Test for overall effect: Z = 3.97 (P < 0.0001)						
**Total (95% CI)**		**13430**		**5662**	**100.0%**	**5.66 [2.75, 11.65]**
Total events	1352		116			
Heterogeneity: Tau² = 0.61; Chi² = 50.81, df = 4 (P < 0.00001); I² = 92%						
Test for overall effect: Z = 4.70 (P < 0.00001)						
Test for subgroup differences: Chi² = 1.81, df = 1 (P = 0.18), I² = 44.8%						

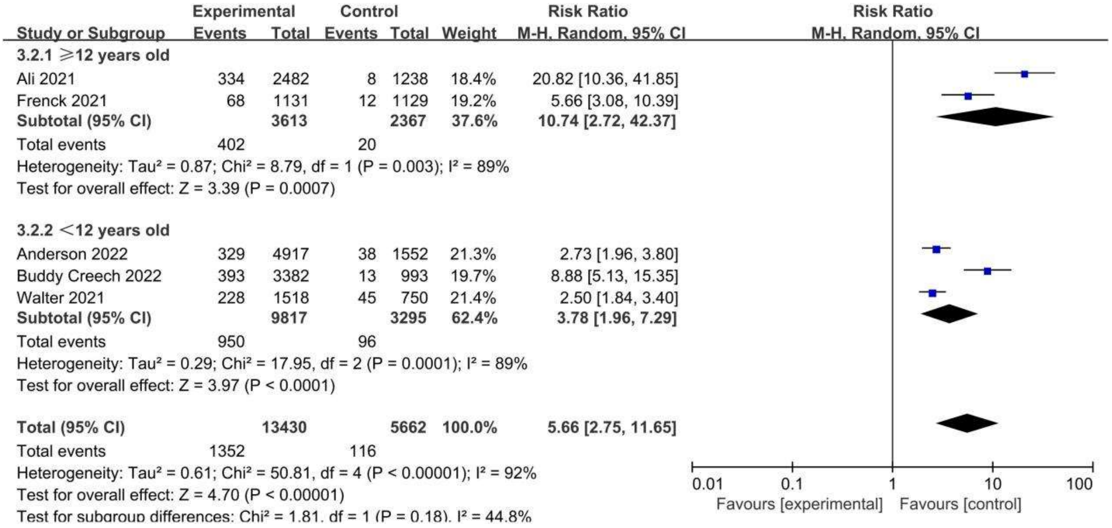

D) Erythema or Redness after dose 2

Study or Subgroup	Experimental Events	Experimental Total	Control Events	Control Total	Weight	Risk Ratio M-H, Random, 95% CI
**3.2.1 ≥12 years old**						
Ali 2021	484	2478	11	1220	19.3%	21.66 [11.96, 39.22]
Frenck 2021	57	1131	12	1129	19.1%	4.74 [2.56, 8.79]
**Subtotal (95% CI)**		**3609**		**2349**	**38.4%**	**10.16 [2.05, 50.29]**
Total events	541		23			
Heterogeneity: Tau² = 1.24; Chi² = 13.96, df = 1 (P = 0.0002); I² = 93%						
Test for overall effect: Z = 2.84 (P = 0.005)						
**3.2.2 <12 years old**						
Anderson 2022	500	4753	35	1485	21.2%	4.46 [3.18, 6.26]
Buddy Creech 2022	640	3367	10	969	19.1%	18.42 [9.90, 34.26]
Walter 2021	288	1514	38	747	21.3%	3.74 [2.70, 5.18]
**Subtotal (95% CI)**		**9634**		**3201**	**61.6%**	**6.45 [2.90, 14.31]**
Total events	1428		83			
Heterogeneity: Tau² = 0.45; Chi² = 23.37, df = 2 (P < 0.00001); I² = 91%						
Test for overall effect: Z = 4.58 (P < 0.00001)						
**Total (95% CI)**		**13243**		**5550**	**100.0%**	**7.73 [3.76, 15.90]**
Total events	1969		106			
Heterogeneity: Tau² = 0.61; Chi² = 48.55, df = 4 (P < 0.00001); I² = 92%						
Test for overall effect: Z = 5.56 (P < 0.00001)						
Test for subgroup differences: Chi² = 0.25, df = 1 (P = 0.62), I² = 0%						

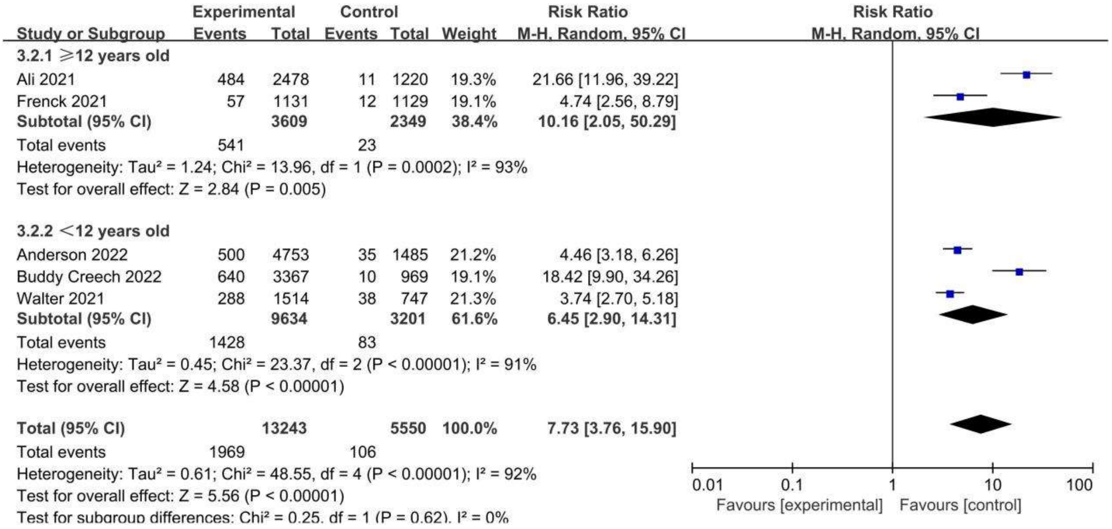

E) Swelling or Hardness after dose 1

Study or Subgroup	Experimental Events	Experimental Total	Control Events	Control Total	Weight	Risk Ratio M-H, Random, 95% CI
**3.3.1 ≥12 years old**						
Ali 2021	403	2482	12	1238	19.5%	16.75 [9.47, 29.62]
Frenck 2021	80	1131	12	1129	19.2%	6.65 [3.65, 12.14]
**Subtotal (95% CI)**		**3613**		**2367**	**38.6%**	**10.61 [4.13, 27.28]**
Total events	483		24			
Heterogeneity: Tau² = 0.38; Chi² = 5.20, df = 1 (P = 0.02); I² = 81%						
Test for overall effect: Z = 4.90 (P < 0.00001)						
**3.3.2 <12 years old**						
Anderson 2022	298	4917	32	1552	21.2%	2.94 [2.05, 4.21]
Buddy Creech 2022	394	3382	12	993	19.5%	9.64 [5.45, 17.05]
Walter 2021	152	1518	23	750	20.7%	3.27 [2.12, 5.02]
**Subtotal (95% CI)**		**9817**		**3295**	**61.4%**	**4.39 [2.24, 8.58]**
Total events	844		67			
Heterogeneity: Tau² = 0.30; Chi² = 13.61, df = 2 (P = 0.001); I² = 85%						
Test for overall effect: Z = 4.31 (P < 0.0001)						
**Total (95% CI)**		**13430**		**5662**	**100.0%**	**6.21 [3.14, 12.28]**
Total events	1327		91			
Heterogeneity: Tau² = 0.54; Chi² = 38.61, df = 4 (P < 0.00001); I² = 90%						
Test for overall effect: Z = 5.26 (P < 0.00001)						
Test for subgroup differences: Chi² = 2.23, df = 1 (P = 0.14), I² = 55.2%						

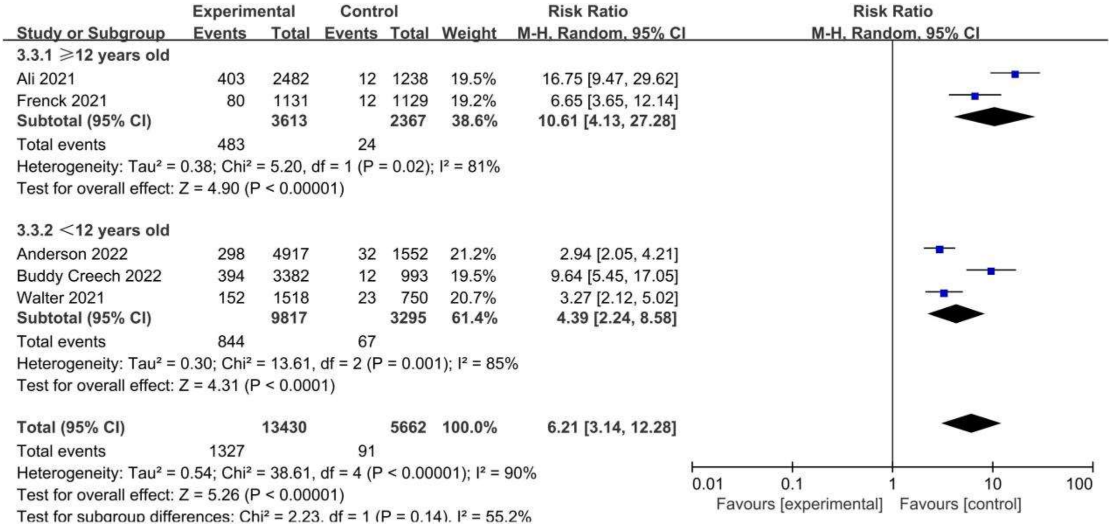

F) Swelling or Hardness after dose 2

Study or Subgroup	Experimental Events	Experimental Total	Control Events	Control Total	Weight	Risk Ratio M-H, Random, 95% CI
**3.3.1 ≥12 years old**						
Ali 2021	509	2478	12	1220	19.4%	20.88 [11.83, 36.86]
Frenck 2021	57	1131	12	1129	18.7%	4.74 [2.56, 8.79]
**Subtotal (95% CI)**		**3609**		**2349**	**38.1%**	**10.00 [2.11, 47.24]**
Total events	566		24			
Heterogeneity: Tau² = 1.16; Chi² = 13.71, df = 1 (P = 0.0002); I² = 93%						
Test for overall effect: Z = 2.90 (P = 0.004)						
**3.3.2 <12 years old**						
Anderson 2022	507	4753	22	1485	21.2%	7.20 [4.72, 10.99]
Buddy Creech 2022	588	3367	12	969	19.4%	14.10 [8.00, 24.86]
Walter 2021	228	1514	23	747	21.2%	4.89 [3.21, 7.44]
**Subtotal (95% CI)**		**9634**		**3201**	**61.9%**	**7.71 [4.33, 13.72]**
Total events	1323		57			
Heterogeneity: Tau² = 0.20; Chi² = 9.20, df = 2 (P = 0.01); I² = 78%						
Test for overall effect: Z = 6.95 (P < 0.00001)						
**Total (95% CI)**		**13243**		**5550**	**100.0%**	**8.59 [4.86, 15.19]**
Total events	1889		81			
Heterogeneity: Tau² = 0.35; Chi² = 25.19, df = 4 (P < 0.0001); I² = 84%						
Test for overall effect: Z = 7.39 (P < 0.00001)						
Test for subgroup differences: Chi² = 0.09, df = 1 (P = 0.76), I² = 0%						

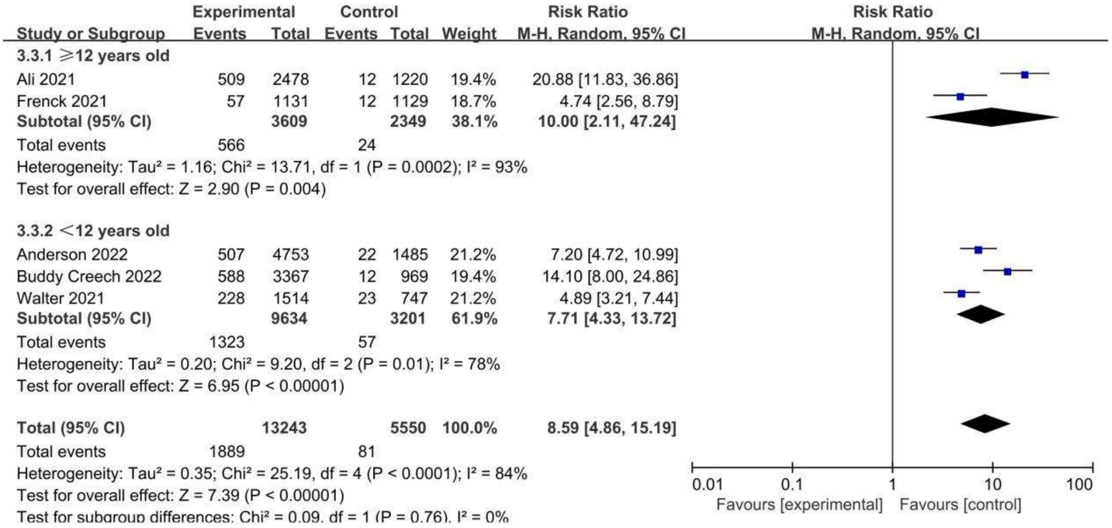

G) Fever after dose 1

Study or Subgroup	Experimental Events	Experimental Total	Control Events	Control Total	Weight	Risk Ratio M-H, Random, 95% CI
**3.4.1 ⩾12 years old**						
Ali 2021	63	2480	12	1238	19.6%	2.62 [1.42, 4.84]
Frenck 2021	114	1131	12	1129	19.8%	9.48 [5.26, 17.09]
**Subtotal (95% CI)**		**3611**		**2367**	**39.4%**	**5.00 [1.40, 17.82]**
Total events	177		24			
Heterogeneity: Tau² = 0.75; Chi² = 8.93, df = 1 (P = 0.003); I² = 89%						
Test for overall effect: Z = 2.48 (P = 0.01)						
**3.4.2 <12 years old**						
Anderson 2022	202	1892	49	582	21.5%	1.27 [0.94, 1.71]
Buddy Creech 2022	110	3381	15	993	20.2%	2.15 [1.26, 3.68]
Walter 2021	107	1518	8	750	18.9%	6.61 [3.24, 13.48]
**Subtotal (95% CI)**		**6791**		**2325**	**60.6%**	**2.50 [1.02, 6.17]**
Total events	419		72			
Heterogeneity: Tau² = 0.56; Chi² = 19.68, df = 2 (P < 0.0001); I² = 90%						
Test for overall effect: Z = 1.99 (P = 0.05)						
**Total (95% CI)**		**10402**		**4692**	**100.0%**	**3.31 [1.47, 7.45]**
Total events	596		96			
Heterogeneity: Tau² = 0.77; Chi² = 49.10, df = 4 (P < 0.00001); I² = 92%						
Test for overall effect: Z = 2.89 (P = 0.004)						
Test for subgroup differences: Chi² = 0.76, df = 1 (P = 0.38), I² = 0%						

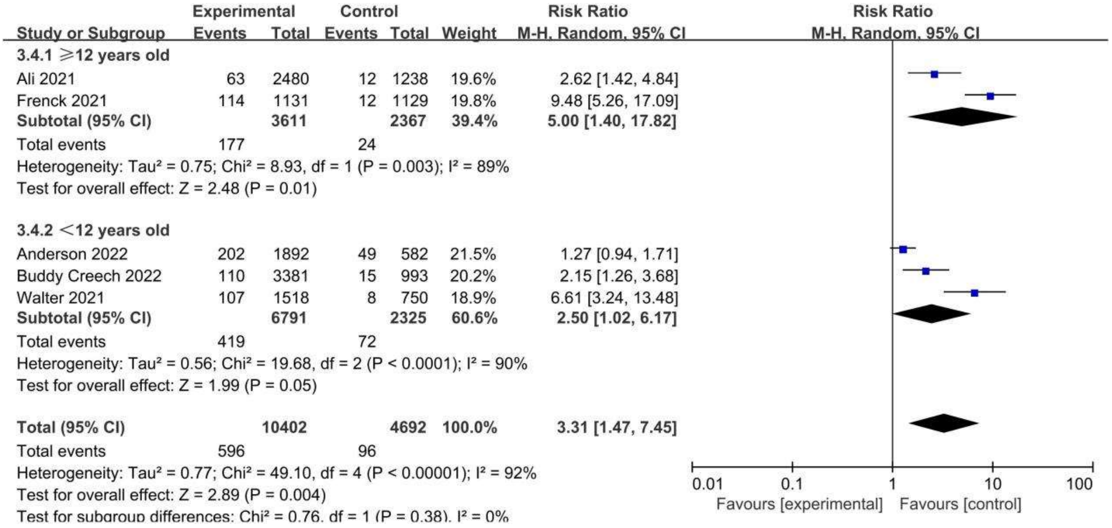

H) Fever after dose 2

Study or Subgroup	Experimental Events	Experimental Total	Control Events	Control Total	Weight	Risk Ratio M-H, Random, 95% CI
**3.4.1 ⩾12 years old**						
Ali 2021	302	2477	12	1219	19.9%	12.39 [6.98, 21.96]
Frenck 2021	227	1131	12	1129	19.9%	18.88 [10.63, 33.55]
**Subtotal (95% CI)**		**3608**		**2348**	**39.7%**	**15.28 [10.11, 23.11]**
Total events	529		24			
Heterogeneity: Tau² = 0.00; Chi² = 1.04, df = 1 (P = 0.31); I² = 4%						
Test for overall effect: Z = 12.92 (P < 0.00001)						
**3.4.2 <12 years old**						
Anderson 2022	249	1744	44	526	20.7%	1.71 [1.26, 2.32]
Buddy Creech 2022	792	3367	19	969	20.3%	12.00 [7.65, 18.80]
Walter 2021	106	1514	8	747	19.3%	6.54 [3.20, 13.34]
**Subtotal (95% CI)**		**6625**		**2242**	**60.3%**	**5.07 [1.14, 22.44]**
Total events	1147		71			
Heterogeneity: Tau² = 1.66; Chi² = 62.74, df = 2 (P < 0.00001); I² = 97%						
Test for overall effect: Z = 2.14 (P = 0.03)						
**Total (95% CI)**		**10233**		**4590**	**100.0%**	**7.85 [2.58, 23.91]**
Total events	1676		95			
Heterogeneity: Tau² = 1.54; Chi² = 103.79, df = 4 (P < 0.00001); I² = 96%						
Test for overall effect: Z = 3.63 (P = 0.0003)						
Test for subgroup differences: Chi² = 1.96, df = 1 (P = 0.16), I² = 49.1%						

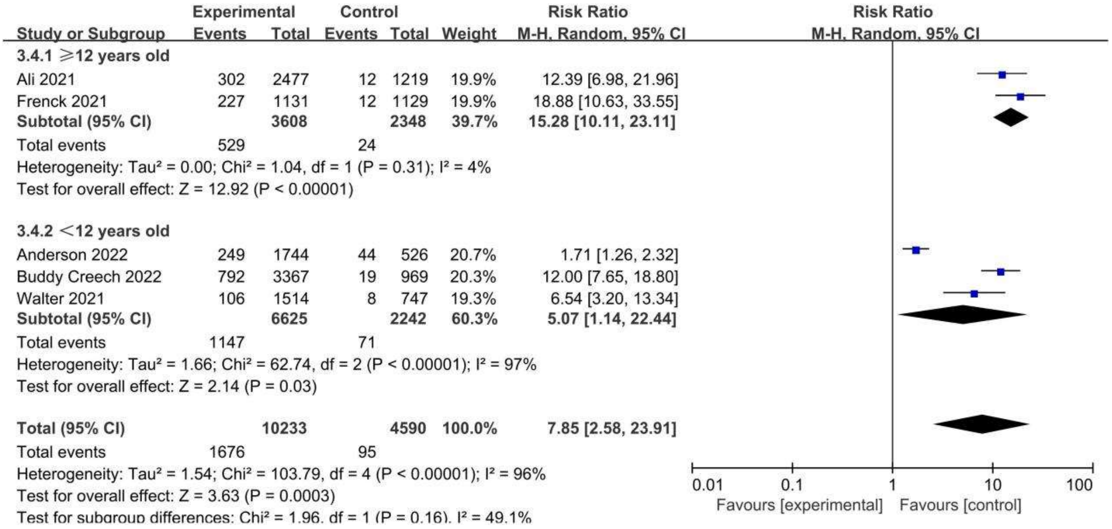

I) Headache after dose 1

Study or Subgroup	Experimental Events	Experimental Total	Control Events	Control Total	Weight	Risk Ratio M-H, Random, 95% CI
**3.5.1 ⩾12 years old**						
Ali 2021	1106	2480	477	1238	25.8%	1.16 [1.07, 1.26]
Frenck 2021	623	1131	396	1129	25.5%	1.57 [1.43, 1.73]
**Subtotal (95% CI)**		**3611**		**2367**	**51.3%**	**1.35 [1.00, 1.82]**
Total events	1729		873			
Heterogeneity: Tau² = 0.04; Chi² = 22.45, df = 1 (P < 0.00001); I² = 96%						
Test for overall effect: Z = 1.95 (P = 0.05)						
**3.5.2 <12 years old**						
Buddy Creech 2022	1047	3380	306	993	25.2%	1.01 [0.90, 1.12]
Walter 2021	334	1518	180	750	23.5%	0.92 [0.78, 1.07]
**Subtotal (95% CI)**		**4898**		**1743**	**48.7%**	**0.98 [0.89, 1.07]**
Total events	1381		486			
Heterogeneity: Tau² = 0.00; Chi² = 0.90, df = 1 (P = 0.34); I² = 0%						
Test for overall effect: Z = 0.52 (P = 0.61)						
**Total (95% CI)**		**8509**		**4110**	**100.0%**	**1.14 [0.92, 1.43]**
Total events	3110		1359			
Heterogeneity: Tau² = 0.05; Chi² = 53.44, df = 3 (P < 0.00001); I² = 94%						
Test for overall effect: Z = 1.18 (P = 0.24)						
Test for subgroup differences: Chi² = 4.07, df = 1 (P = 0.04), I² = 75.4%						

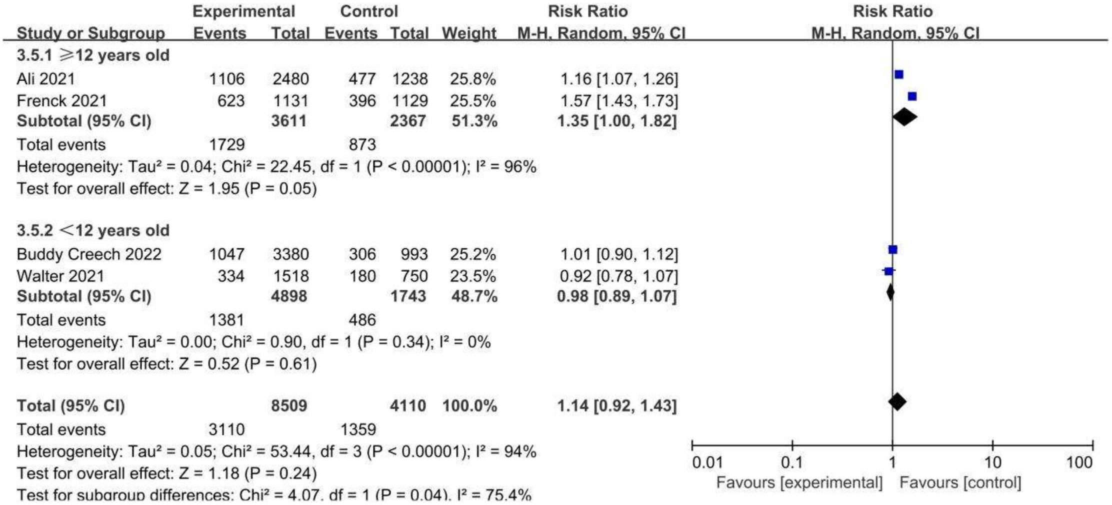

J) Headache after dose 2

Study or Subgroup	Experimental Events	Experimental Total	Control Events	Control Total	Weight	Risk Ratio M-H, Random, 95% CI
**3.5.1 ⩾12 years old**						
Ali 2021	1739	2478	370	1220	25.9%	2.31 [2.12, 2.53]
Frenck 2021	736	1131	271	1129	25.3%	2.71 [2.42, 3.03]
**Subtotal (95% CI)**		**3609**		**2349**	**51.1%**	**2.50 [2.14, 2.91]**
Total events	2475		641			
Heterogeneity: Tau² = 0.01; Chi² = 4.71, df = 1 (P = 0.03); I² = 79%						
Test for overall effect: Z = 11.55 (P < 0.00001)						
**3.5.2 <12 years old**						
Buddy Creech 2022	1740	3365	275	969	25.5%	1.82 [1.64, 2.02]
Walter 2021	424	1514	142	747	23.4%	1.47 [1.24, 1.74]
**Subtotal (95% CI)**		**4879**		**1716**	**48.9%**	**1.66 [1.35, 2.04]**
Total events	2164		417			
Heterogeneity: Tau² = 0.02; Chi² = 4.39, df = 1 (P = 0.04); I² = 77%						
Test for overall effect: Z = 4.77 (P < 0.00001)						
**Total (95% CI)**		**8488**		**4065**	**100.0%**	**2.04 [1.63, 2.56]**
Total events	4639		1058			
Heterogeneity: Tau² = 0.05; Chi² = 47.97, df = 3 (P < 0.00001); I² = 94%						
Test for overall effect: Z = 6.16 (P < 0.00001)						
Test for subgroup differences: Chi² = 9.64, df = 1 (P = 0.002), I² = 89.6%						

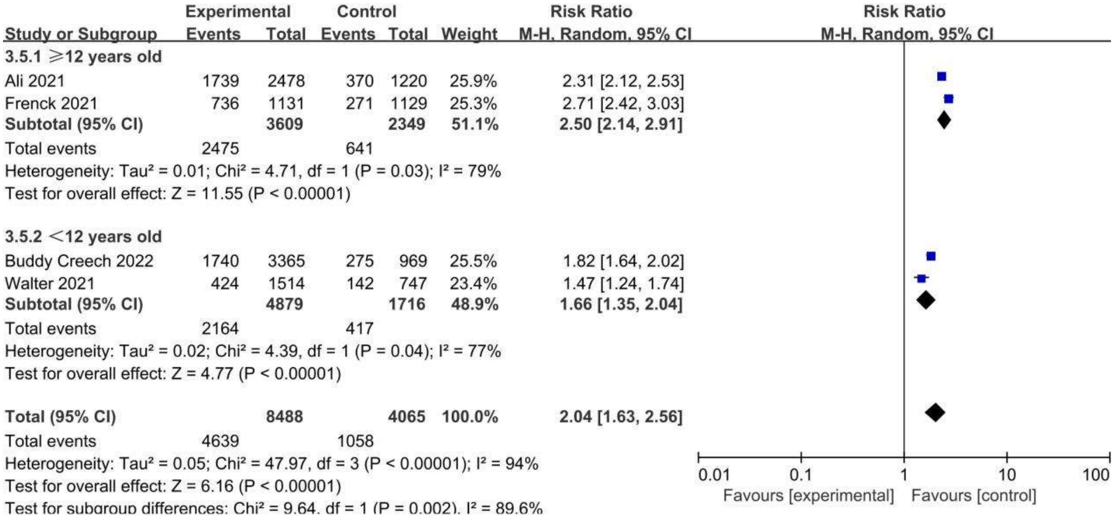

K) Fatigue after dose 1

Study or Subgroup	Experimental Events	Experimental Total	Control Events	Control Total	Weight	Risk Ratio M-H, Random, 95% CI
**3.6.1 ≥12 years old**						
Ali 2021	1188	2481	453	1238	26.5%	1.31 [1.20, 1.42]
Frenck 2021	679	1131	463	1129	26.5%	1.46 [1.35, 1.59]
**Subtotal (95% CI)**		**3612**		**2367**	**53.0%**	**1.38 [1.24, 1.55]**
Total events	1867		916			
Heterogeneity: Tau² = 0.00; Chi² = 3.43, df = 1 (P = 0.06); I² = 71%						
Test for overall effect: Z = 5.77 (P < 0.00001)						
**3.6.2 <12 years old**						
Buddy Creech 2022	1452	3380	334	993	25.3%	1.28 [1.16, 1.41]
Walter 2021	517	1518	233	750	21.7%	1.10 [0.97, 1.25]
**Subtotal (95% CI)**		**4898**		**1743**	**47.0%**	**1.19 [1.03, 1.38]**
Total events	1969		567			
Heterogeneity: Tau² = 0.01; Chi² = 3.53, df = 1 (P = 0.06); I² = 72%						
Test for overall effect: Z = 2.29 (P = 0.02)						
**Total (95% CI)**		**8510**		**4110**	**100.0%**	**1.29 [1.16, 1.43]**
Total events	3836		1483			
Heterogeneity: Tau² = 0.01; Chi² = 14.50, df = 3 (P = 0.002); I² = 79%						
Test for overall effect: Z = 4.72 (P < 0.00001)						
Test for subgroup differences: Chi² = 2.53, df = 1 (P = 0.11), I² = 60.4%						

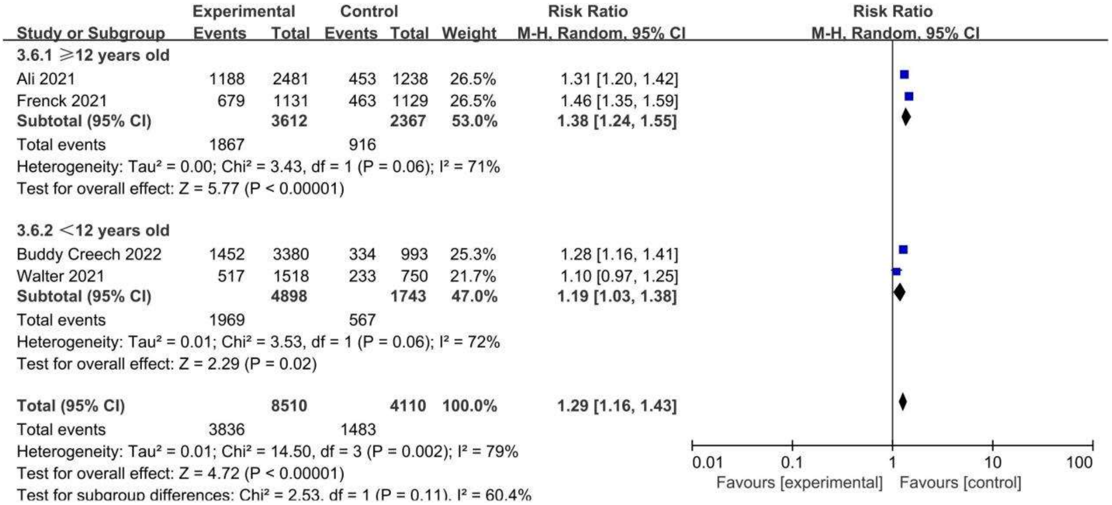

L) Fatigue after dose 2

Study or Subgroup	Experimental Events	Experimental Total	Control Events	Control Total	Weight	Risk Ratio M-H, Random, 95% CI
**3.6.1 ≥12 years old**						
Ali 2021	1679	2478	353	1220	25.6%	2.34 [2.14, 2.57]
Frenck 2021	747	1131	283	1129	25.0%	2.63 [2.36, 2.94]
**Subtotal (95% CI)**		**3609**		**2349**	**50.6%**	**2.47 [2.20, 2.78]**
Total events	2426		636			
Heterogeneity: Tau² = 0.00; Chi² = 2.63, df = 1 (P = 0.10); I² = 62%						
Test for overall effect: Z = 15.37 (P < 0.00001)						
**3.6.2 <12 years old**						
Buddy Creech 2022	2141	3365	335	969	25.6%	1.84 [1.68, 2.01]
Walter 2021	591	1514	180	747	23.8%	1.62 [1.41, 1.87]
**Subtotal (95% CI)**		**4879**		**1716**	**49.4%**	**1.75 [1.55, 1.98]**
Total events	2732		515			
Heterogeneity: Tau² = 0.00; Chi² = 2.21, df = 1 (P = 0.14); I² = 55%						
Test for overall effect: Z = 8.92 (P < 0.00001)						
**Total (95% CI)**		**8488**		**4065**	**100.0%**	**2.08 [1.70, 2.54]**
Total events	5158		1151			
Heterogeneity: Tau² = 0.04; Chi² = 42.94, df = 3 (P < 0.00001); I² = 93%						
Test for overall effect: Z = 7.18 (P < 0.00001)						
Test for subgroup differences: Chi² = 16.34, df = 1 (P < 0.0001), I² = 93.9%						

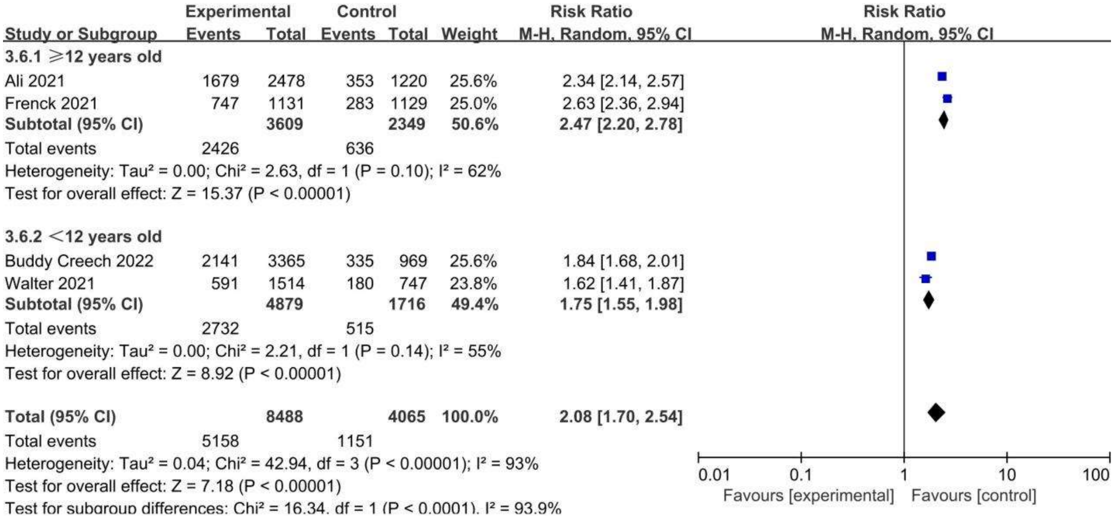

M) Myalgia after dose 1

Study or Subgroup	Experimental Events	Experimental Total	Control Events	Control Total	Weight	Risk Ratio M-H, Fixed, 95% CI
**3.7.1 ⩾12 years old**						
Ali 2021	668	2480	205	1238	42.7%	1.63 [1.41, 1.87]
Frenck 2021	272	1131	147	1129	23.0%	1.85 [1.54, 2.22]
**Subtotal (95% CI)**		**3611**		**2367**	**65.7%**	**1.70 [1.52, 1.90]**
Total events	940		352			
Heterogeneity: Chi² = 1.16, df = 1 (P = 0.28); I² = 14%						
Test for overall effect: Z = 9.36 (P < 0.00001)						
**3.7.2 <12 years old**						
Buddy Creech 2022	478	3380	96	993	23.2%	1.46 [1.19, 1.80]
Walter 2021	137	1518	53	750	11.1%	1.28 [0.94, 1.73]
**Subtotal (95% CI)**		**4898**		**1743**	**34.3%**	**1.40 [1.18, 1.67]**
Total events	615		149			
Heterogeneity: Chi² = 0.52, df = 1 (P = 0.47); I² = 0%						
Test for overall effect: Z = 3.87 (P = 0.0001)						
**Total (95% CI)**		**8509**		**4110**	**100.0%**	**1.60 [1.46, 1.76]**
Total events	1555		501			
Heterogeneity: Chi² = 5.24, df = 3 (P = 0.16); I² = 43%						
Test for overall effect: Z = 9.86 (P < 0.00001)						
Test for subgroup differences: Chi² = 3.47, df = 1 (P = 0.06), I² = 71.2%						

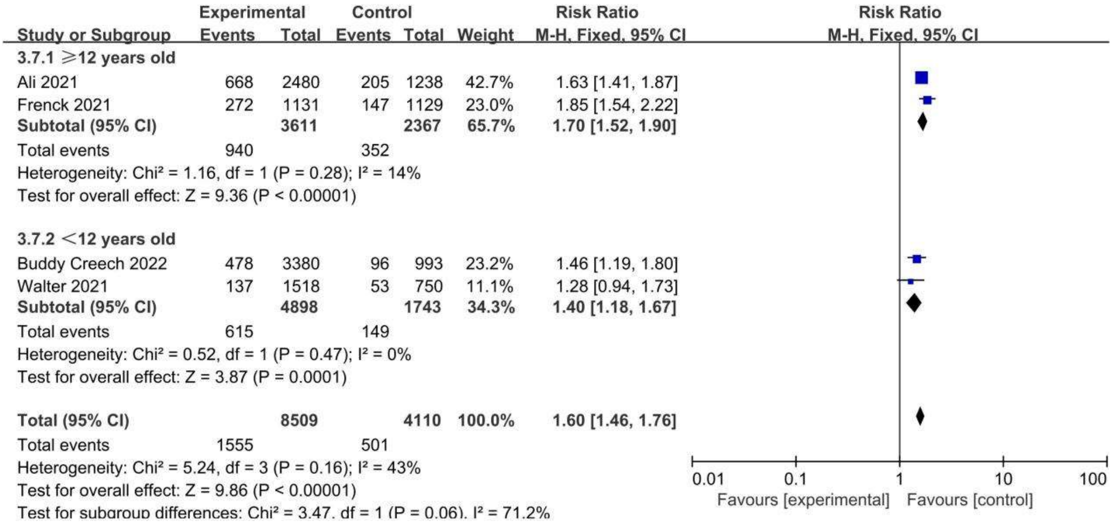

N) Myalgia after dose 2

Study or Subgroup	Experimental Events	Experimental Total	Control Events	Control Total	Weight	Risk Ratio M-H, Random, 95% CI
**3.7.1 ⩾12 years old**						
Ali 2021	1154	2477	153	1220	26.4%	3.71 [3.18, 4.33]
Frenck 2021	362	1131	91	1129	25.0%	3.97 [3.20, 4.92]
**Subtotal (95% CI)**		**3608**		**2349**	**51.4%**	**3.80 [3.35, 4.31]**
Total events	1516		244			
Heterogeneity: Tau² = 0.00; Chi² = 0.25, df = 1 (P = 0.62); I² = 0%						
Test for overall effect: Z = 20.91 (P < 0.00001)						
**3.7.2 <12 years old**						
Buddy Creech 2022	932	3365	105	969	25.7%	2.56 [2.12, 3.09]
Walter 2021	182	1514	53	747	22.9%	1.69 [1.26, 2.27]
**Subtotal (95% CI)**		**4879**		**1716**	**48.6%**	**2.11 [1.41, 3.16]**
Total events	1114		158			
Heterogeneity: Tau² = 0.07; Chi² = 5.37, df = 1 (P = 0.02); I² = 81%						
Test for overall effect: Z = 3.64 (P = 0.0003)						
**Total (95% CI)**		**8487**		**4065**	**100.0%**	**2.87 [2.07, 3.98]**
Total events	2630		402			
Heterogeneity: Tau² = 0.10; Chi² = 30.75, df = 3 (P < 0.00001); I² = 90%						
Test for overall effect: Z = 6.32 (P < 0.00001)						
Test for subgroup differences: Chi² = 7.43, df = 1 (P = 0.006), I² = 86.5%						

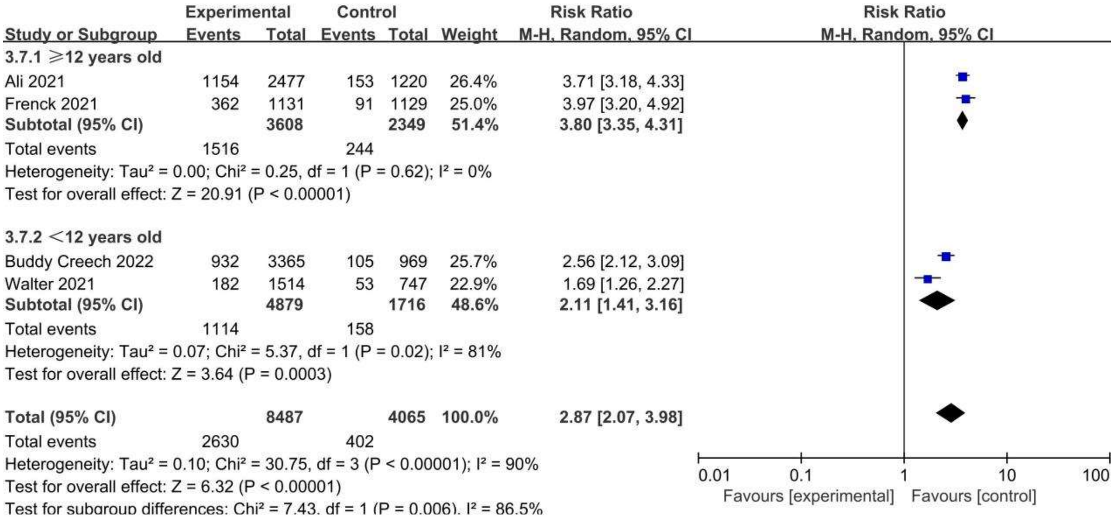

O) Arthralgia after dose 1

Study or Subgroup	Experimental Events	Experimental Total	Control Events	Control Total	Weight	Risk Ratio M-H, Random, 95% CI
**3.8.1 ≥12 years old**						
Ali 2021	371	2480	143	1238	29.4%	1.30 [1.08, 1.55]
Frenck 2021	114	1131	80	1129	25.2%	1.42 [1.08, 1.87]
**Subtotal (95% CI)**		**3611**		**2367**	**54.6%**	**1.33 [1.15, 1.55]**
Total events	485		223			
Heterogeneity: Tau² = 0.00; Chi² = 0.31, df = 1 (P = 0.57); I² = 0%						
Test for overall effect: Z = 3.73 (P = 0.0002)						
**3.8.2 <12 years old**						
Buddy Creech 2022	287	3380	75	993	26.6%	1.12 [0.88, 1.44]
Walter 2021	46	1518	38	750	18.8%	0.60 [0.39, 0.91]
**Subtotal (95% CI)**		**4898**		**1743**	**45.4%**	**0.84 [0.45, 1.56]**
Total events	333		113			
Heterogeneity: Tau² = 0.17; Chi² = 6.47, df = 1 (P = 0.01); I² = 85%						
Test for overall effect: Z = 0.55 (P = 0.58)						
**Total (95% CI)**		**8509**		**4110**	**100.0%**	**1.10 [0.84, 1.45]**
Total events	818		336			
Heterogeneity: Tau² = 0.06; Chi² = 12.96, df = 3 (P = 0.005); I² = 77%						
Test for overall effect: Z = 0.71 (P = 0.48)						
Test for subgroup differences: Chi² = 2.03, df = 1 (P = 0.15), I² = 50.7%						

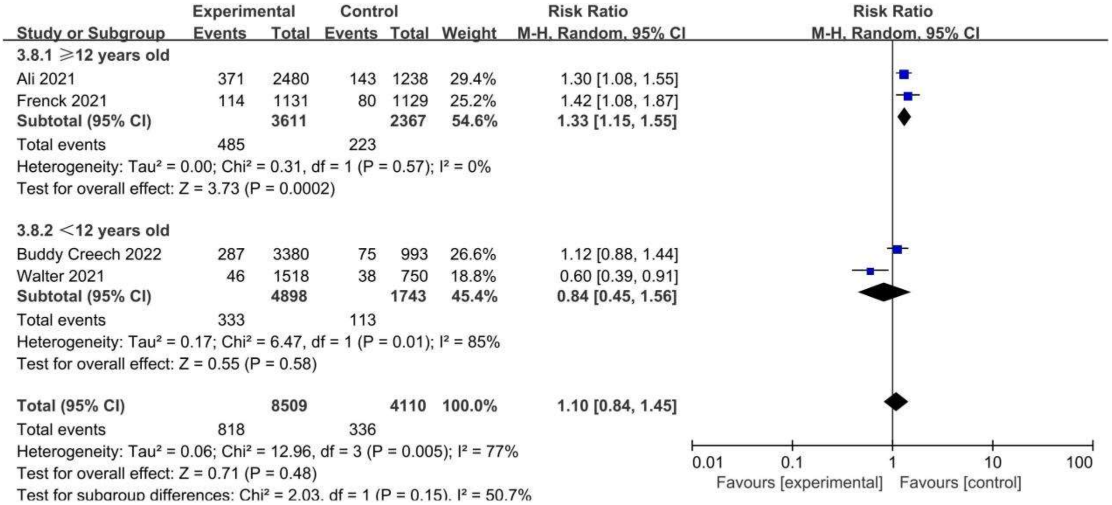

P) Arthralgia after dose 2

Study or Subgroup	Experimental Events	Experimental Total	Control Events	Control Total	Weight	Risk Ratio M-H, Random, 95% CI
**3.8.1 ≥12 years old**						
Ali 2021	716	2477	113	1220	27.0%	3.12 [2.59, 3.76]
Frenck 2021	181	1131	57	1129	24.9%	3.17 [2.38, 4.22]
**Subtotal (95% CI)**		**3608**		**2349**	**51.9%**	**3.14 [2.68, 3.66]**
Total events	897		170			
Heterogeneity: Tau² = 0.00; Chi² = 0.01, df = 1 (P = 0.93); I² = 0%						
Test for overall effect: Z = 14.35 (P < 0.00001)						
**3.8.2 <12 years old**						
Buddy Creech 2022	525	3365	84	969	26.4%	1.80 [1.45, 2.24]
Walter 2021	76	1514	30	747	21.7%	1.25 [0.83, 1.89]
**Subtotal (95% CI)**		**4879**		**1716**	**48.1%**	**1.57 [1.11, 2.22]**
Total events	601		114			
Heterogeneity: Tau² = 0.04; Chi² = 2.34, df = 1 (P = 0.13); I² = 57%						
Test for overall effect: Z = 2.54 (P = 0.01)						
**Total (95% CI)**		**8487**		**4065**	**100.0%**	**2.22 [1.50, 3.28]**
Total events	1498		284			
Heterogeneity: Tau² = 0.14; Chi² = 27.45, df = 3 (P < 0.00001); I² = 89%						
Test for overall effect: Z = 4.02 (P < 0.0001)						
Test for subgroup differences: Chi² = 12.76, df = 1 (P = 0.0004), I² = 92.2%						

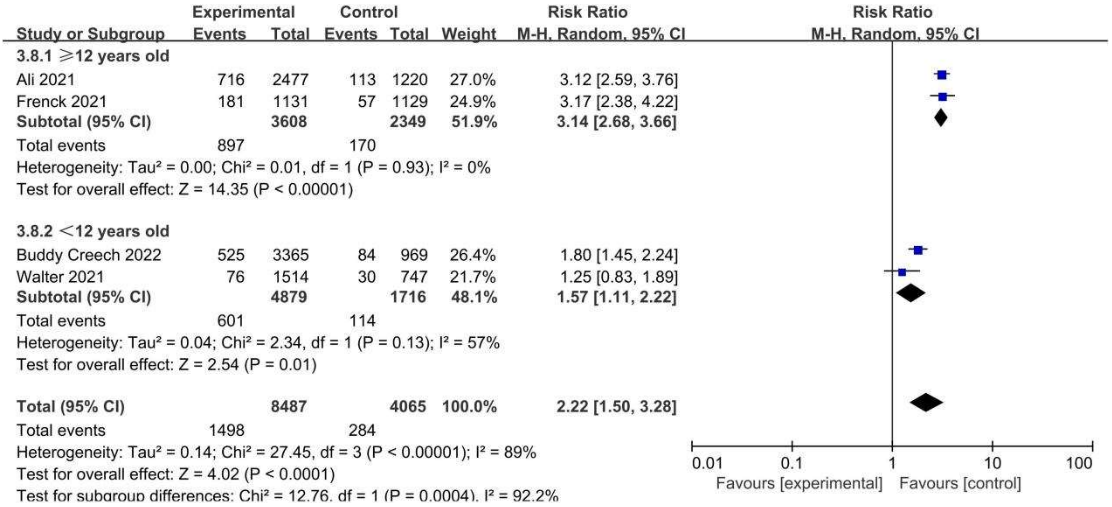

Q) Chills after dose 1

Study or Subgroup	Experimental Events	Experimental Total	Control Events	Control Total	Weight	Risk Ratio M-H, Random, 95% CI
**3.9.1 ≥12 years old**						
Ali 2021	456	2480	138	1238	26.8%	1.65 [1.38, 1.97]
Frenck 2021	317	1131	120	1129	26.5%	2.64 [2.17, 3.20]
**Subtotal (95% CI)**		**3611**		**2367**	**53.3%**	**2.08 [1.31, 3.30]**
Total events	773		258			
Heterogeneity: Tau² = 0.10; Chi² = 12.27, df = 1 (P = 0.0005); I² = 92%						
Test for overall effect: Z = 3.13 (P = 0.002)						
**3.9.2 <12 years old**						
Buddy Creech 2022	342	3380	67	993	25.1%	1.50 [1.17, 1.93]
Walter 2021	76	1518	38	750	21.6%	0.99 [0.68, 1.44]
**Subtotal (95% CI)**		**4898**		**1743**	**46.7%**	**1.25 [0.83, 1.87]**
Total events	418		105			
Heterogeneity: Tau² = 0.06; Chi² = 3.23, df = 1 (P = 0.07); I² = 69%						
Test for overall effect: Z = 1.07 (P = 0.28)						
**Total (95% CI)**		**8509**		**4110**	**100.0%**	**1.63 [1.15, 2.33]**
Total events	1191		363			
Heterogeneity: Tau² = 0.11; Chi² = 27.54, df = 3 (P < 0.00001); I² = 89%						
Test for overall effect: Z = 2.71 (P = 0.007)						
Test for subgroup differences: Chi² = 2.67, df = 1 (P = 0.10), I² = 62.6%						

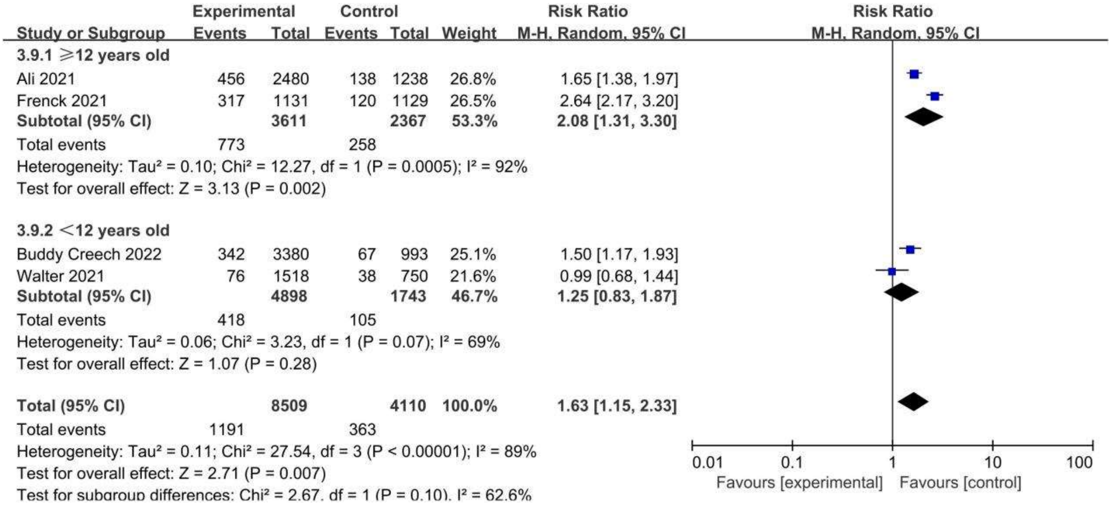

R) Chills after dose 2

Study or Subgroup	Experimental Events	Experimental Total	Control Events	Control Total	Weight	Risk Ratio M-H, Random, 95% CI
**3.9.1 ≥12 years old**						
Ali 2021	1066	2477	91	1220	26.6%	5.77 [4.71, 7.07]
Frenck 2021	476	1131	80	1129	26.1%	5.94 [4.76, 7.42]
**Subtotal (95% CI)**		**3608**		**2349**	**52.7%**	**5.85 [5.03, 6.79]**
Total events	1542		171			
Heterogeneity: Tau² = 0.00; Chi² = 0.04, df = 1 (P = 0.85); I² = 0%						
Test for overall effect: Z = 23.12 (P < 0.00001)						
**3.9.2 <12 years old**						
Buddy Creech 2022	981	3365	74	969	26.0%	3.82 [3.05, 4.78]
Walter 2021	152	1514	30	747	21.2%	2.50 [1.71, 3.66]
**Subtotal (95% CI)**		**4879**		**1716**	**47.3%**	**3.18 [2.11, 4.80]**
Total events	1133		104			
Heterogeneity: Tau² = 0.06; Chi² = 3.53, df = 1 (P = 0.06); I² = 72%						
Test for overall effect: Z = 5.50 (P < 0.00001)						
**Total (95% CI)**		**8487**		**4065**	**100.0%**	**4.37 [3.14, 6.09]**
Total events	2675		275			
Heterogeneity: Tau² = 0.10; Chi² = 21.90, df = 3 (P < 0.0001); I² = 86%						
Test for overall effect: Z = 8.73 (P < 0.00001)						
Test for subgroup differences: Chi² = 7.40, df = 1 (P = 0.007), I² = 86.5%						

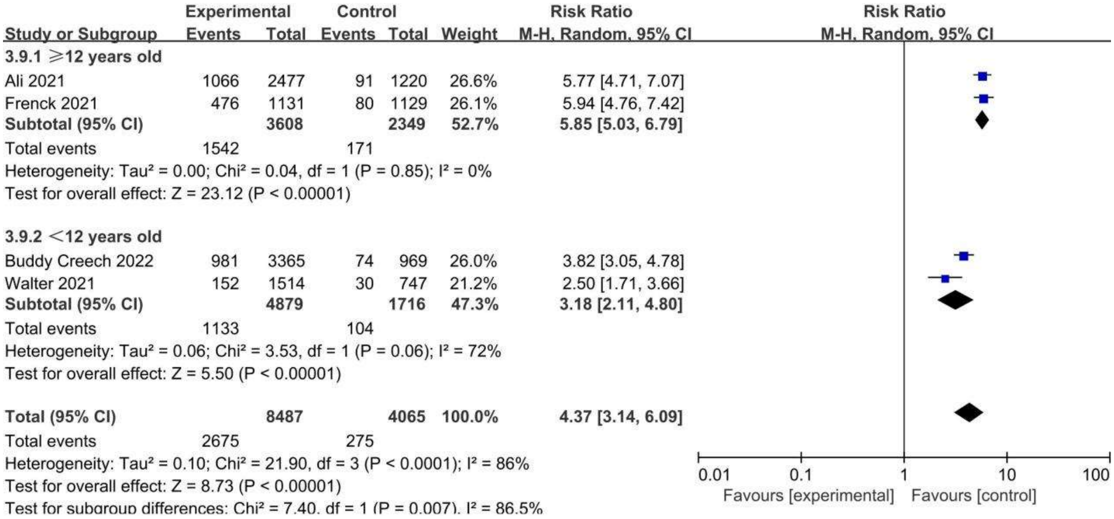

S) Nausea or Vomiting after dose 1

Study or Subgroup	Experimental Events	Experimental Total	Control Events	Control Total	Weight	Risk Ratio M-H, Random, 95% CI
**3.10.1 ≥12 years old**						
Ali 2021	281	2480	110	1238	34.9%	1.28 [1.03, 1.57]
Frenck 2021	34	1131	12	1129	16.6%	2.83 [1.47, 5.43]
**Subtotal (95% CI)**		**3611**		**2367**	**51.4%**	**1.78 [0.82, 3.86]**
Total events	315		122			
Heterogeneity: Tau² = 0.26; Chi² = 5.20, df = 1 (P = 0.02); I² = 81%						
Test for overall effect: Z = 1.47 (P = 0.14)						
**3.10.2 <12 years old**						
Buddy Creech 2022	361	3380	107	993	35.1%	0.99 [0.81, 1.22]
Walter 2021	31	1518	8	750	13.4%	1.91 [0.88, 4.14]
**Subtotal (95% CI)**		**4898**		**1743**	**48.6%**	**1.24 [0.67, 2.27]**
Total events	392		115			
Heterogeneity: Tau² = 0.13; Chi² = 2.62, df = 1 (P = 0.11); I² = 62%						
Test for overall effect: Z = 0.68 (P = 0.50)						
**Total (95% CI)**		**8509**		**4110**	**100.0%**	**1.41 [0.99, 1.99]**
Total events	707		237			
Heterogeneity: Tau² = 0.08; Chi² = 11.78, df = 3 (P = 0.008); I² = 75%						
Test for overall effect: Z = 1.93 (P = 0.05)						
Test for subgroup differences: Chi² = 0.54, df = 1 (P = 0.46), I² = 0%						

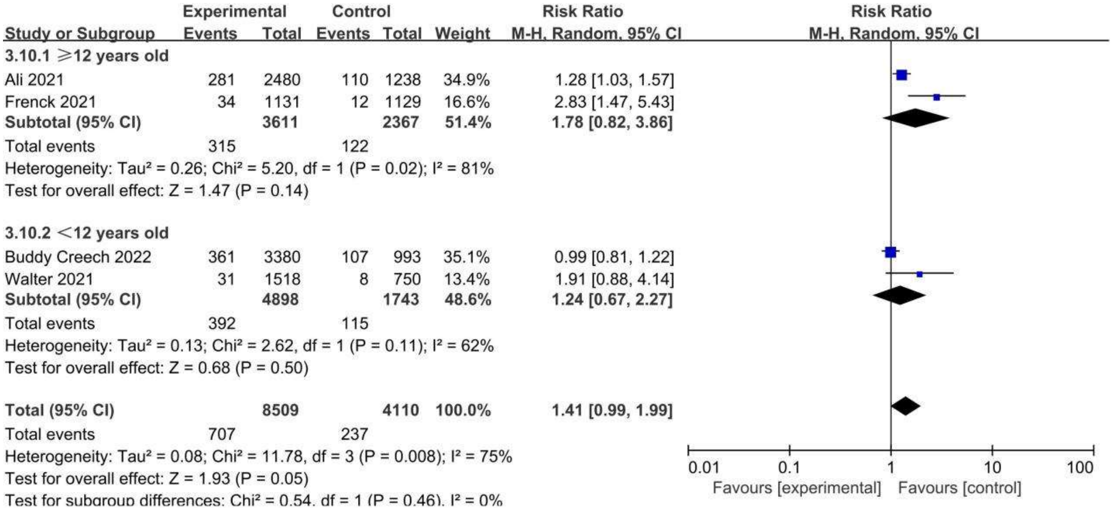

T) Nausea or Vomiting after dose 2

Study or Subgroup	Experimental Events	Experimental Total	Control Events	Control Total	Weight	Risk Ratio M-H, Fixed, 95% CI
**3.10.1 ≥12 years old**						
Ali 2021	591	2447	106	1220	44.9%	2.78 [2.29, 3.38]
Frenck 2021	34	1131	12	1129	3.8%	2.83 [1.47, 5.43]
**Subtotal (95% CI)**		**3578**		**2349**	**48.8%**	**2.78 [2.31, 3.36]**
Total events	625		118			
Heterogeneity: Chi² = 0.00, df = 1 (P = 0.96); I² = 0%						
Test for overall effect: Z = 10.74 (P < 0.00001)						
**3.10.2 <12 years old**						
Buddy Creech 2022	795	3365	97	969	47.8%	2.36 [1.94, 2.88]
Walter 2021	31	1514	8	747	3.4%	1.91 [0.88, 4.14]
**Subtotal (95% CI)**		**4879**		**1716**	**51.2%**	**2.33 [1.92, 2.82]**
Total events	826		105			
Heterogeneity: Chi² = 0.27, df = 1 (P = 0.60); I² = 0%						
Test for overall effect: Z = 8.64 (P < 0.00001)						
**Total (95% CI)**		**8457**		**4065**	**100.0%**	**2.55 [2.23, 2.92]**
Total events	1451		223			
Heterogeneity: Chi² = 1.97, df = 3 (P = 0.58); I² = 0%						
Test for overall effect: Z = 13.72 (P < 0.00001)						
Test for subgroup differences: Chi² = 1.69, df = 1 (P = 0.19), I² = 40.8%						

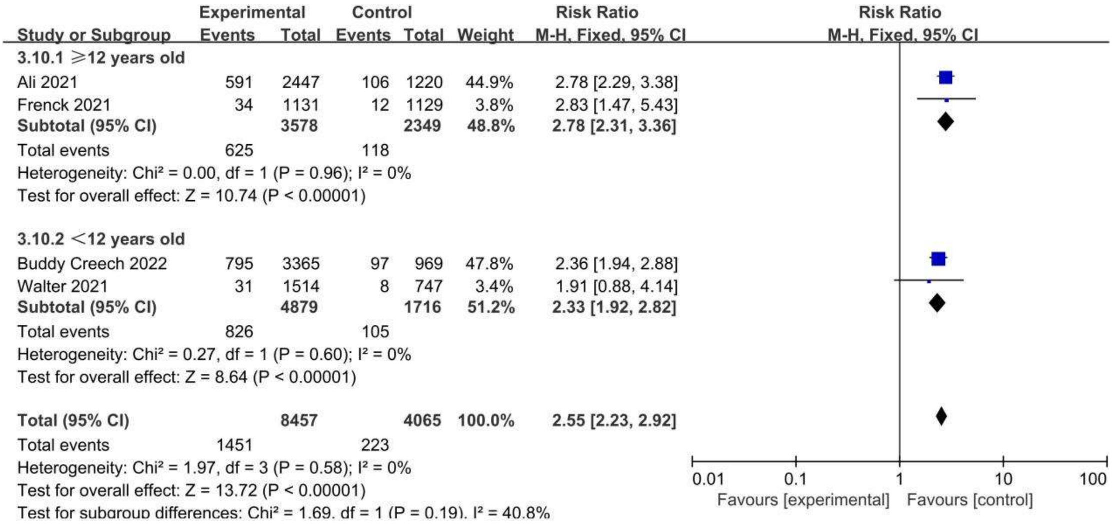

Figure S10. Specific adverse reactions in the mRNA vaccine recipients aged 12 -15 years versus 5 - 11 years:

A) After dose 1

	12 - 15 years old		5 - 11 years old			Risk Ratio
Study or Subgroup	Events	Total	Events	Total	Weight	M-H, Random, 95% CI
**3.11.1 Local pain**						
Frenck 2021	86	1131	23	1129	7.2%	3.73 [2.37, 5.87]
Walter 2021	74	1511	31	749	7.4%	1.18 [0.79, 1.78]
**Subtotal (95% CI)**		**2642**		**1878**	**14.5%**	**2.09 [0.68, 6.48]**
Total events	160		54			
Heterogeneity: Tau² = 0.62; Chi² = 13.71, df = 1 (P = 0.0002); I² = 93%						
Test for overall effect: Z = 1.28 (P = 0.20)						
**3.11.2 Erythema or Redness**						
Frenck 2021	6	1131	1	1129	1.9%	5.99 [0.72, 49.67]
Walter 2021	15	1511	6	749	5.0%	1.24 [0.48, 3.18]
**Subtotal (95% CI)**		**2642**		**1878**	**6.9%**	**2.04 [0.48, 8.74]**
Total events	21		7			
Heterogeneity: Tau² = 0.57; Chi² = 1.82, df = 1 (P = 0.18); I² = 45%						
Test for overall effect: Z = 0.96 (P = 0.34)						
**3.11.3 Swelling or Hardness**						
Frenck 2021	7	1131	1	1129	1.9%	6.99 [0.86, 56.70]
Walter 2021	10	1511	3	749	3.7%	1.65 [0.46, 5.99]
**Subtotal (95% CI)**		**2642**		**1878**	**5.6%**	**2.67 [0.69, 10.30]**
Total events	17		4			
Heterogeneity: Tau² = 0.28; Chi² = 1.36, df = 1 (P = 0.24); I² = 26%						
Test for overall effect: Z = 1.43 (P = 0.15)						
**3.11.4 Fever**						
Frenck 2021	10	1131	1	1129	2.0%	9.98 [1.28, 77.85]
Walter 2021	3	1511	1	749	1.7%	1.49 [0.15, 14.27]
**Subtotal (95% CI)**		**2642**		**1878**	**3.7%**	**4.09 [0.61, 27.34]**
Total events	13		2			
Heterogeneity: Tau² = 0.67; Chi² = 1.55, df = 1 (P = 0.21); I² = 36%						
Test for overall effect: Z = 1.45 (P = 0.15)						
**3.11.5 Headache**						
Frenck 2021	55	1131	35	1129	7.3%	1.57 [1.04, 2.38]
Walter 2021	22	1511	24	749	6.7%	0.45 [0.26, 0.80]
**Subtotal (95% CI)**		**2642**		**1878**	**14.0%**	**0.86 [0.25, 2.89]**
Total events	77		59			
Heterogeneity: Tau² = 0.70; Chi² = 11.81, df = 1 (P = 0.0006); I² = 92%						
Test for overall effect: Z = 0.25 (P = 0.80)						
**3.11.6 Fatigue**						
Frenck 2021	60	1131	41	1129	7.4%	1.46 [0.99, 2.15]
Walter 2021	34	1511	31	749	7.1%	0.54 [0.34, 0.88]
**Subtotal (95% CI)**		**2642**		**1878**	**14.5%**	**0.90 [0.34, 2.37]**
Total events	94		72			
Heterogeneity: Tau² = 0.44; Chi² = 9.88, df = 1 (P = 0.002); I² = 90%						
Test for overall effect: Z = 0.21 (P = 0.83)						
**3.11.7 Myalgia**						
Frenck 2021	24	1131	13	1129	6.2%	1.84 [0.94, 3.60]
Walter 2021	9	1511	7	749	4.8%	0.64 [0.24, 1.70]
**Subtotal (95% CI)**		**2642**		**1878**	**11.0%**	**1.15 [0.41, 3.25]**
Total events	33		20			
Heterogeneity: Tau² = 0.38; Chi² = 3.06, df = 1 (P = 0.08); I² = 67%						
Test for overall effect: Z = 0.27 (P = 0.78)						
**3.11.8 Arthralgia**						
Frenck 2021	10	1131	7	1129	4.9%	1.43 [0.54, 3.73]
Walter 2021	3	1511	5	749	3.3%	0.30 [0.07, 1.24]
**Subtotal (95% CI)**		**2642**		**1878**	**8.2%**	**0.71 [0.16, 3.28]**
Total events	13		12			
Heterogeneity: Tau² = 0.84; Chi² = 3.18, df = 1 (P = 0.07); I² = 69%						
Test for overall effect: Z = 0.43 (P = 0.67)						
**3.11.9 Nausea or Vomiting**						
Frenck 2021	3	1131	1	1129	1.7%	2.99 [0.31, 28.75]
Walter 2021	2	1511	1	749	1.6%	0.99 [0.09, 10.92]
**Subtotal (95% CI)**		**2642**		**1878**	**3.3%**	**1.78 [0.34, 9.23]**
Total events	5		2			
Heterogeneity: Tau² = 0.00; Chi² = 0.43, df = 1 (P = 0.51); I² = 0%						
Test for overall effect: Z = 0.69 (P = 0.49)						
**3.11.10 Chills**						
Frenck 2021	28	1131	10	1129	6.0%	2.80 [1.36, 5.73]
Walter 2021	5	1511	5	749	3.9%	0.50 [0.14, 1.71]
**Subtotal (95% CI)**		**2642**		**1878**	**9.8%**	**1.27 [0.23, 6.89]**
Total events	33		15			
Heterogeneity: Tau² = 1.23; Chi² = 5.64, df = 1 (P = 0.02); I² = 82%						
Test for overall effect: Z = 0.28 (P = 0.78)						
**3.11.11 Diarrhea**						
Frenck 2021	8	1131	7	1129	4.7%	1.14 [0.42, 3.14]
Walter 2021	6	1511	4	749	3.8%	0.74 [0.21, 2.63]
**Subtotal (95% CI)**		**2642**		**1878**	**8.5%**	**0.97 [0.44, 2.12]**
Total events	14		11			
Heterogeneity: Tau² = 0.00; Chi² = 0.27, df = 1 (P = 0.60); I² = 0%						
Test for overall effect: Z = 0.09 (P = 0.93)						
**Total (95% CI)**		**29062**		**20658**	**100.0%**	**1.31 [0.94, 1.82]**
Total events	480		258			
Heterogeneity: Tau² = 0.35; Chi² = 72.18, df = 21 (P < 0.00001); I² = 71%						
Test for overall effect: Z = 1.58 (P = 0.11)						
Test for subgroup differences: Chi² = 5.87, df = 10 (P = 0.83), I² = 0%						

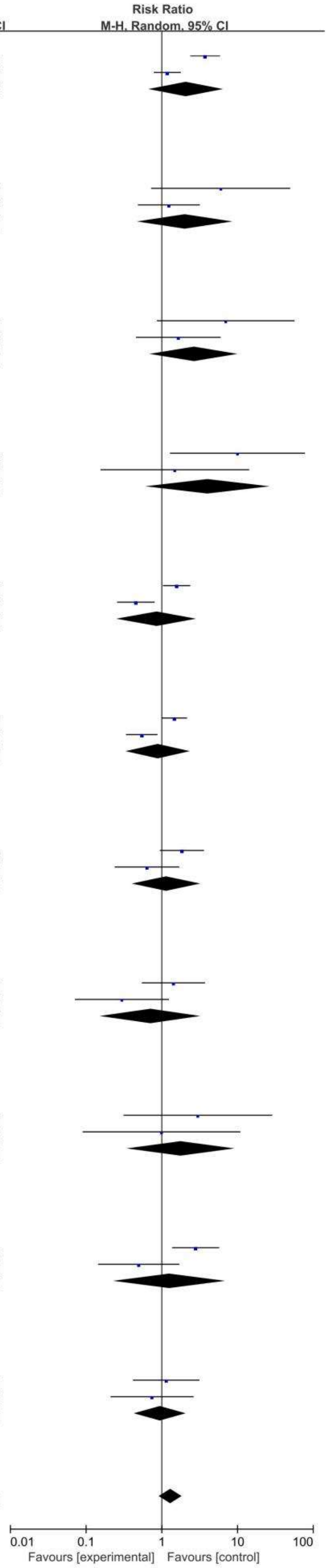

B) After dose 2

Study or Subgroup	12 - 15 years old Events	12 - 15 years old Total	5 - 11 years old Events	5 - 11 years old Total	Weight	Risk Ratio M-H, Random, 95% CI
**3.12.1 Local pain**						
Frenck 2021	79	1131	18	1129	6.5%	4.38 [2.64, 7.26]
Walter 2021	71	1501	29	741	6.7%	1.21 [0.79, 1.84]
**Subtotal (95% CI)**		**2632**		**1870**	**13.2%**	**2.28 [0.64, 8.14]**
Total events	150		47			
Heterogeneity: Tau² = 0.78; Chi² = 14.88, df = 1 (P = 0.0001); I² = 93%						
Test for overall effect: Z = 1.27 (P = 0.20)						
**3.12.2 Erythema or Redness**						
Frenck 2021	5	1131	1	1129	2.3%	4.99 [0.58, 42.65]
Walter 2021	19	1501	5	741	5.0%	1.88 [0.70, 5.00]
**Subtotal (95% CI)**		**2632**		**1870**	**7.3%**	**2.22 [0.91, 5.42]**
Total events	24		6			
Heterogeneity: Tau² = 0.00; Chi² = 0.66, df = 1 (P = 0.42); I² = 0%						
Test for overall effect: Z = 1.75 (P = 0.08)						
**3.12.3 Swelling or Hardness**						
Frenck 2021	5	1131	1	1129	2.3%	4.99 [0.58, 42.65]
Walter 2021	15	1501	3	741	4.2%	2.47 [0.72, 8.50]
**Subtotal (95% CI)**		**2632**		**1870**	**6.5%**	**2.94 [1.01, 8.59]**
Total events	20		4			
Heterogeneity: Tau² = 0.00; Chi² = 0.31, df = 1 (P = 0.58); I² = 0%						
Test for overall effect: Z = 1.97 (P = 0.05)						
**3.12.4 Fever**						
Frenck 2021	20	1131	1	1129	2.5%	19.96 [2.68, 148.51]
Walter 2021	7	1501	1	741	2.3%	3.46 [0.43, 28.04]
**Subtotal (95% CI)**		**2632**		**1870**	**4.8%**	**8.52 [1.46, 49.60]**
Total events	27		2			
Heterogeneity: Tau² = 0.52; Chi² = 1.48, df = 1 (P = 0.22); I² = 32%						
Test for overall effect: Z = 2.38 (P = 0.02)						
**3.12.5 Headache**						
Frenck 2021	65	1131	24	1129	6.6%	2.70 [1.71, 4.29]
Walter 2021	28	1501	19	741	6.3%	0.73 [0.41, 1.29]
**Subtotal (95% CI)**		**2632**		**1870**	**12.9%**	**1.42 [0.39, 5.15]**
Total events	93		43			
Heterogeneity: Tau² = 0.79; Chi² = 12.22, df = 1 (P = 0.0005); I² = 92%						
Test for overall effect: Z = 0.53 (P = 0.59)						
**3.12.6 Fatigue**						
Frenck 2021	66	1131	25	1129	6.6%	2.64 [1.68, 4.14]
Walter 2021	39	1501	24	741	6.5%	0.80 [0.49, 1.32]
**Subtotal (95% CI)**		**2632**		**1870**	**13.1%**	**1.46 [0.45, 4.70]**
Total events	105		49			
Heterogeneity: Tau² = 0.65; Chi² = 11.97, df = 1 (P = 0.0005); I² = 92%						
Test for overall effect: Z = 0.64 (P = 0.52)						
**3.12.7 Myalgia**						
Frenck 2021	32	1131	8	1129	5.7%	3.99 [1.85, 8.63]
Walter 2021	12	1501	7	741	5.2%	0.85 [0.33, 2.14]
**Subtotal (95% CI)**		**2632**		**1870**	**10.8%**	**1.88 [0.41, 8.66]**
Total events	44		15			
Heterogeneity: Tau² = 1.03; Chi² = 6.43, df = 1 (P = 0.01); I² = 84%						
Test for overall effect: Z = 0.81 (P = 0.42)						
**3.12.8 Arthralgia**						
Frenck 2021	16	1131	5	1129	4.9%	3.19 [1.17, 8.69]
Walter 2021	5	1501	4	741	4.0%	0.62 [0.17, 2.29]
**Subtotal (95% CI)**		**2632**		**1870**	**8.9%**	**1.49 [0.30, 7.45]**
Total events	21		9			
Heterogeneity: Tau² = 1.01; Chi² = 3.84, df = 1 (P = 0.05); I² = 74%						
Test for overall effect: Z = 0.48 (P = 0.63)						
**3.12.9 Nausea or Vomiting**						
Frenck 2021	3	1131	1	1129	2.1%	2.99 [0.31, 28.75]
Walter 2021	2	1501	1	741	1.9%	0.99 [0.09, 10.87]
**Subtotal (95% CI)**		**2632**		**1870**	**4.0%**	**1.78 [0.34, 9.21]**
Total events	5		2			
Heterogeneity: Tau² = 0.00; Chi² = 0.44, df = 1 (P = 0.51); I² = 0%						
Test for overall effect: Z = 0.68 (P = 0.49)						
**3.12.10 Chills**						
Frenck 2021	42	1131	7	1129	5.6%	5.99 [2.70, 13.27]
Walter 2021	10	1501	4	741	4.4%	1.23 [0.39, 3.92]
**Subtotal (95% CI)**		**2632**		**1870**	**10.0%**	**2.88 [0.61, 13.62]**
Total events	52		11			
Heterogeneity: Tau² = 1.01; Chi² = 4.93, df = 1 (P = 0.03); I² = 80%						
Test for overall effect: Z = 1.33 (P = 0.18)						
**3.12.11 Diarrhea**						
Frenck 2021	6	1131	4	1129	4.1%	1.50 [0.42, 5.29]
Walter 2021	5	1501	5	156	4.2%	0.10 [0.03, 0.36]
**Subtotal (95% CI)**		**2632**		**1285**	**8.4%**	**0.39 [0.03, 5.82]**
Total events	11		9			
Heterogeneity: Tau² = 3.38; Chi² = 9.36, df = 1 (P = 0.002); I² = 89%						
Test for overall effect: Z = 0.68 (P = 0.50)						
**Total (95% CI)**		**28952**		**19985**	**100.0%**	**1.84 [1.25, 2.72]**
Total events	552		197			
Heterogeneity: Tau² = 0.54; Chi² = 90.99, df = 21 (P < 0.00001); I² = 77%						
Test for overall effect: Z = 3.08 (P = 0.002)						
Test for subgroup differences: Chi² = 5.44, df = 10 (P = 0.86), I² = 0%						

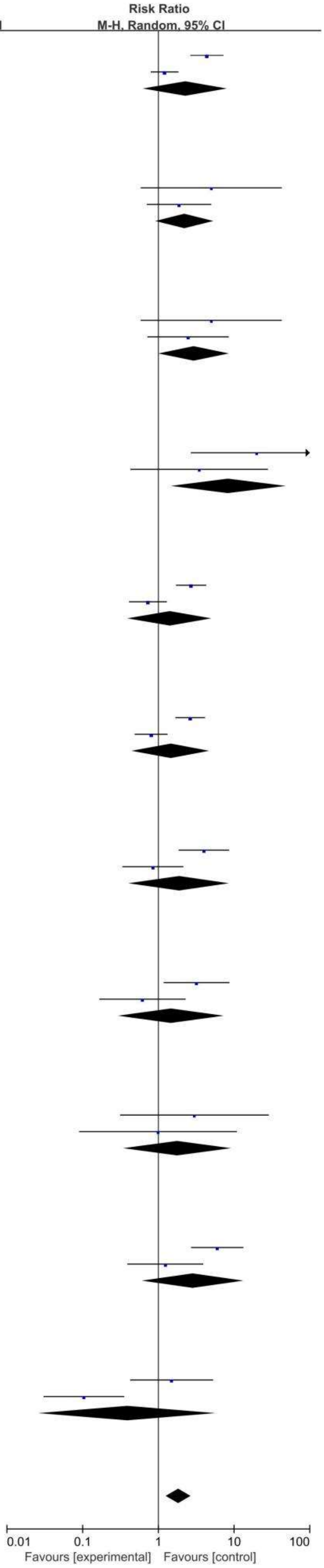

Figure S11. Specific adverse reactions in the mRNA vaccine recipients aged 6 - 23 months versus 2 - 5 years:

A) After dose 1

	6-23 months		2-5 years old			Risk Ratio
Study or Subgroup	Events	Total	Events	Total	Weight	M-H, Fixed, 95% CI
**3.4.1 Any local adverse reactions**						
Anderson 2022	835	1894	1914	3025	44.7%	0.70 [0.66, 0.74]
**Subtotal (95% CI)**		**1894**		**3025**	**44.7%**	**0.70 [0.66, 0.74]**
Total events	835		1914			
Heterogeneity: Not applicable						
Test for overall effect: Z = 12.31 (P < 0.00001)						
**3.4.2 Local pain**						
Anderson 2022	700	1893	1852	3023	43.3%	0.60 [0.57, 0.64]
**Subtotal (95% CI)**		**1893**		**3023**	**43.3%**	**0.60 [0.57, 0.64]**
Total events	700		1852			
Heterogeneity: Not applicable						
Test for overall effect: Z = 15.16 (P < 0.00001)						
**3.4.3 Erythema / Redness**						
Anderson 2022	161	1893	168	3024	3.9%	1.53 [1.24, 1.89]
**Subtotal (95% CI)**		**1893**		**3024**	**3.9%**	**1.53 [1.24, 1.89]**
Total events	161		168			
Heterogeneity: Not applicable						
Test for overall effect: Z = 4.01 (P < 0.0001)						
**3.4.4 Swelling / Hardness**						
Anderson 2022	160	1893	138	3024	3.2%	1.85 [1.49, 2.31]
**Subtotal (95% CI)**		**1893**		**3024**	**3.2%**	**1.85 [1.49, 2.31]**
Total events	160		138			
Heterogeneity: Not applicable						
Test for overall effect: Z = 5.48 (P < 0.00001)						
**3.4.5 Axillary Swelling**						
Anderson 2022	117	1892	206	3023	4.8%	0.91 [0.73, 1.13]
**Subtotal (95% CI)**		**1892**		**3023**	**4.8%**	**0.91 [0.73, 1.13]**
Total events	117		206			
Heterogeneity: Not applicable						
Test for overall effect: Z = 0.87 (P = 0.39)						
**Total (95% CI)**		**9465**		**15119**	**100.0%**	**0.74 [0.71, 0.77]**
Total events	1973		4278			
Heterogeneity: Chi² = 157.42, df = 4 (P < 0.00001); I² = 97%						
Test for overall effect: Z = 14.60 (P < 0.00001)						
Test for subgroup differences: Chi² = 153.93, df = 4 (P < 0.00001), I² = 97.4%						

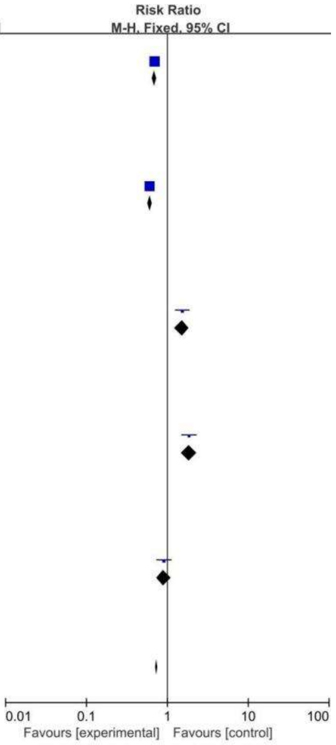

B) After dose 2

	6-23 months		2-5 years old			Risk Ratio
Study or Subgroup	Events	Total	Events	Total	Weight	M-H, Fixed, 95% CI
**3.4.1 Any local adverse reactions**						
Anderson 2022	939	1746	2212	3007	43.0%	0.73 [0.70, 0.77]
**Subtotal (95% CI)**		**1746**		**3007**	**43.0%**	**0.73 [0.70, 0.77]**
Total events	939		2212			
Heterogeneity: Not applicable						
Test for overall effect: Z = 12.66 (P < 0.00001)						
**3.4.2 Local pain**						
Anderson 2022	796	1746	2153	3007	41.9%	0.64 [0.60, 0.67]
**Subtotal (95% CI)**		**1746**		**3007**	**41.9%**	**0.64 [0.60, 0.67]**
Total events	796		2153			
Heterogeneity: Not applicable						
Test for overall effect: Z = 15.81 (P < 0.00001)						
**3.4.3 Erythema / Redness**						
Anderson 2022	235	1746	265	3007	5.2%	1.53 [1.29, 1.80]
**Subtotal (95% CI)**		**1746**		**3007**	**5.2%**	**1.53 [1.29, 1.80]**
Total events	235		265			
Heterogeneity: Not applicable						
Test for overall effect: Z = 5.02 (P < 0.00001)						
**3.4.4 Swelling / Hardness**						
Anderson 2022	261	1746	246	3007	4.8%	1.83 [1.55, 2.15]
**Subtotal (95% CI)**		**1746**		**3007**	**4.8%**	**1.83 [1.55, 2.15]**
Total events	261		246			
Heterogeneity: Not applicable						
Test for overall effect: Z = 7.21 (P < 0.00001)						
**3.4.5 Axillary Swelling**						
Anderson 2022	159	1746	268	3007	5.2%	1.02 [0.85, 1.23]
**Subtotal (95% CI)**		**1746**		**3007**	**5.2%**	**1.02 [0.85, 1.23]**
Total events	159		268			
Heterogeneity: Not applicable						
Test for overall effect: Z = 0.23 (P = 0.82)						
**Total (95% CI)**		**8730**		**15035**	**100.0%**	**0.80 [0.77, 0.83]**
Total events	2390		5144			
Heterogeneity: Chi² = 240.08, df = 4 (P < 0.00001); I² = 98%						
Test for overall effect: Z = 12.44 (P < 0.00001)						
Test for subgroup differences: Chi² = 228.74, df = 4 (P < 0.00001), I² = 98.3%						

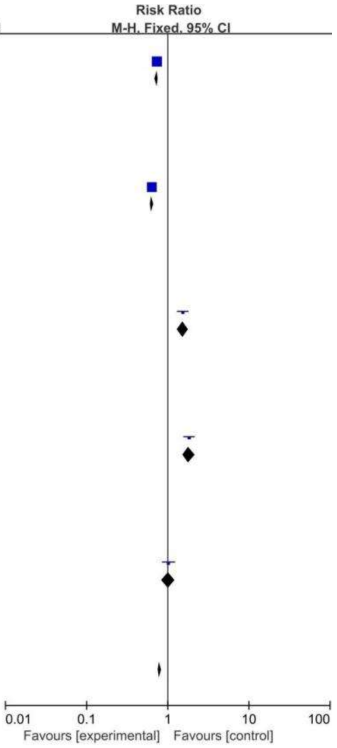

Figure S12. Adverse reactions in the inactivated vaccine group of different ages versus the control group after whole vaccination:

A) Local pain

Study or Subgroup	Experimental Events	Experimental Total	Control Events	Control Total	Weight	Risk Ratio M-H, Fixed, 95% CI
**3.2.1 ⩾12 years old**						
Han 2021	46	161	1	42	3.9%	12.00 [1.70, 84.50]
Xia 2022	23	180	12	60	44.1%	0.64 [0.34, 1.20]
**Subtotal (95% CI)**		**341**		**102**	**48.0%**	**1.56 [0.88, 2.77]**
Total events	69		13			
Heterogeneity: Chi² = 11.81, df = 1 (P = 0.0006); I² = 92%						
Test for overall effect: Z = 1.51 (P = 0.13)						
**3.2.2 <12 years old**						
Han 2021	25	275	1	72	3.9%	6.55 [0.90, 47.50]
Xia 2022	23	432	13	140	48.1%	0.57 [0.30, 1.10]
**Subtotal (95% CI)**		**707**		**212**	**52.0%**	**1.02 [0.57, 1.84]**
Total events	48		14			
Heterogeneity: Chi² = 6.37, df = 1 (P = 0.01); I² = 84%						
Test for overall effect: Z = 0.06 (P = 0.95)						
**Total (95% CI)**		**1048**		**314**	**100.0%**	**1.28 [0.85, 1.93]**
Total events	117		27			
Heterogeneity: Chi² = 18.06, df = 3 (P = 0.0004); I² = 83%						
Test for overall effect: Z = 1.18 (P = 0.24)						
Test for subgroup differences: Chi² = 1.02, df = 1 (P = 0.31), I² = 2.3%						

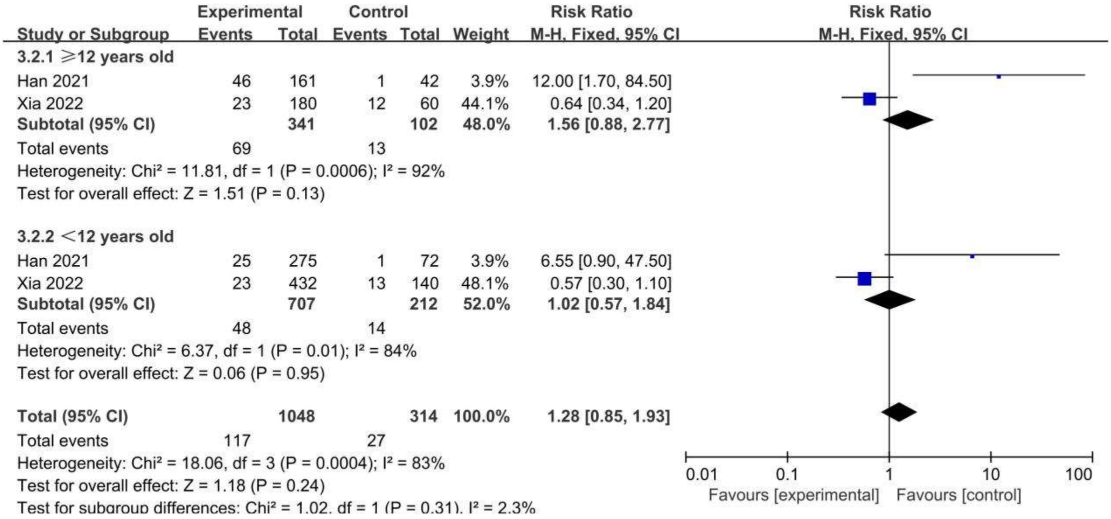

B) Erythema or Redness

Study or Subgroup	Experimental Events	Experimental Total	Control Events	Control Total	Weight	Risk Ratio M-H, Fixed, 95% CI
**3.2.1 ⩾12 years old**						
Han 2021	1	161	0	42	8.7%	0.80 [0.03, 19.20]
Xia 2022	0	54	1	18	24.6%	0.12 [0.00, 2.71]
**Subtotal (95% CI)**		**215**		**60**	**33.3%**	**0.29 [0.04, 2.18]**
Total events	1		1			
Heterogeneity: Chi² = 0.71, df = 1 (P = 0.40); I² = 0%						
Test for overall effect: Z = 1.20 (P = 0.23)						
**3.2.2 <12 years old**						
Han 2021	0	275	0	72		Not estimable
Xia 2022	7	432	4	140	66.7%	0.57 [0.17, 1.91]
**Subtotal (95% CI)**		**707**		**212**	**66.7%**	**0.57 [0.17, 1.91]**
Total events	7		4			
Heterogeneity: Not applicable						
Test for overall effect: Z = 0.92 (P = 0.36)						
**Total (95% CI)**		**922**		**272**	**100.0%**	**0.48 [0.17, 1.33]**
Total events	8		5			
Heterogeneity: Chi² = 0.96, df = 2 (P = 0.62); I² = 0%						
Test for overall effect: Z = 1.42 (P = 0.16)						
Test for subgroup differences: Chi² = 0.30, df = 1 (P = 0.58), I² = 0%						

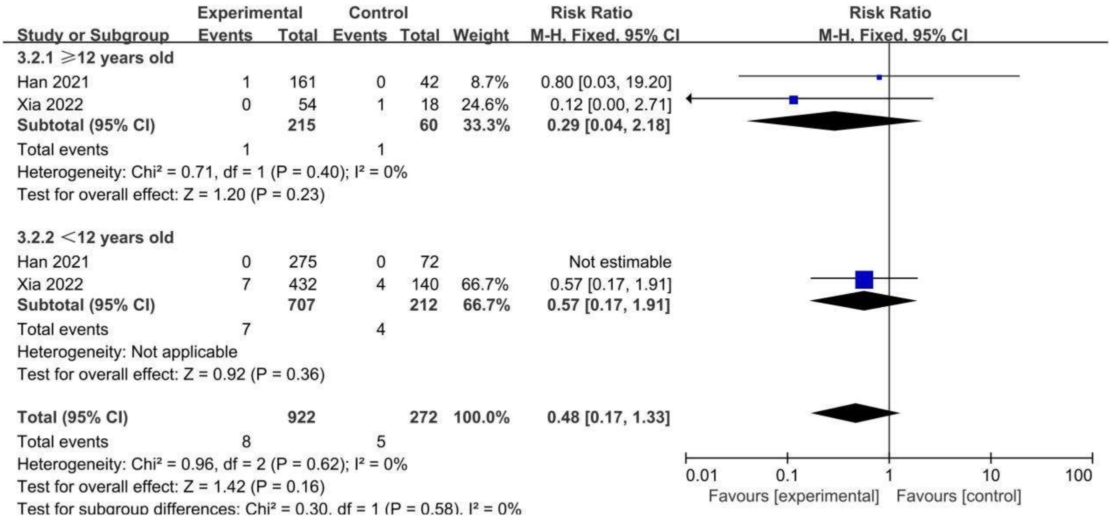

C) Swelling or Hardness

Study or Subgroup	Experimental Events	Experimental Total	Control Events	Control Total	Weight	Risk Ratio M-H, Fixed, 95% CI
**3.2.1 ≥12 years old**						
Han 2021	6	161	1	42	25.9%	1.57 [0.19, 12.65]
Xia 2022	0	54	1	18	36.4%	0.12 [0.00, 2.71]
**Subtotal (95% CI)**		**215**		**60**	**62.4%**	**0.72 [0.16, 3.15]**
Total events	6		2			
Heterogeneity: Chi² = 1.82, df = 1 (P = 0.18); I² = 45%						
Test for overall effect: Z = 0.44 (P = 0.66)						
**3.2.2 <12 years old**						
Han 2021	3	275	0	72	12.9%	1.85 [0.10, 35.44]
Xia 2022	3	432	1	140	24.7%	0.97 [0.10, 9.27]
**Subtotal (95% CI)**		**707**		**212**	**37.6%**	**1.27 [0.22, 7.55]**
Total events	6		1			
Heterogeneity: Chi² = 0.12, df = 1 (P = 0.73); I² = 0%						
Test for overall effect: Z = 0.27 (P = 0.79)						
**Total (95% CI)**		**922**		**272**	**100.0%**	**0.93 [0.30, 2.87]**
Total events	12		3			
Heterogeneity: Chi² = 2.13, df = 3 (P = 0.55); I² = 0%						
Test for overall effect: Z = 0.13 (P = 0.90)						
Test for subgroup differences: Chi² = 0.24, df = 1 (P = 0.63), I² = 0%						

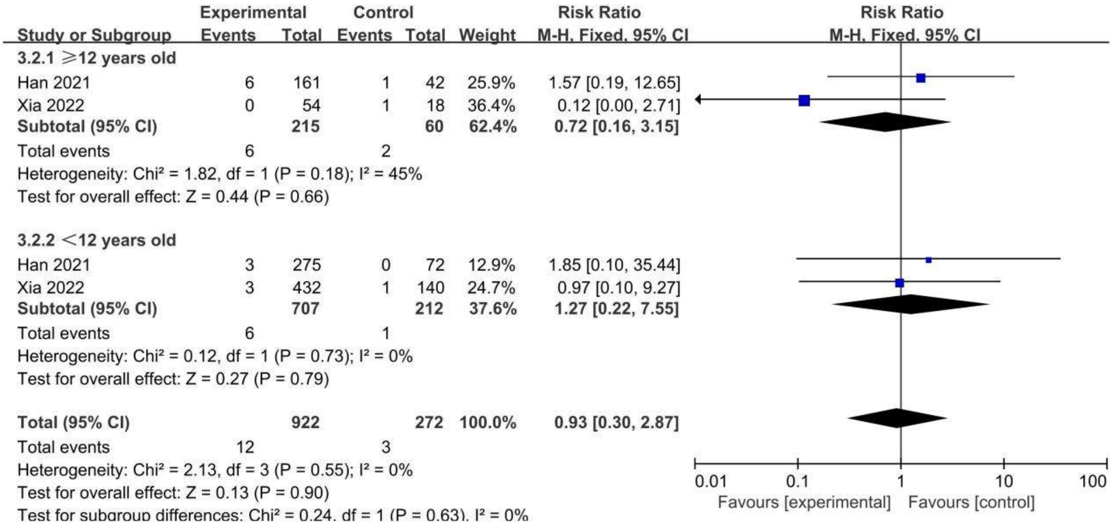

D) Fever

Study or Subgroup	Experimental Events	Experimental Total	Control Events	Control Total	Weight	Risk Ratio M-H, Fixed, 95% CI
**3.2.1 ≥12 years old**						
Han 2021	3	161	0	42	3.3%	1.86 [0.10, 35.29]
Xia 2022	12	180	4	60	25.3%	1.00 [0.34, 2.98]
**Subtotal (95% CI)**		**341**		**102**	**28.6%**	**1.10 [0.40, 3.06]**
Total events	15		4			
Heterogeneity: Chi² = 0.15, df = 1 (P = 0.70); I² = 0%						
Test for overall effect: Z = 0.18 (P = 0.86)						
**3.2.2 <12 years old**						
Han 2021	17	275	5	72	33.4%	0.89 [0.34, 2.33]
Xia 2022	21	432	6	144	38.0%	1.17 [0.48, 2.83]
**Subtotal (95% CI)**		**707**		**216**	**71.4%**	**1.04 [0.54, 1.99]**
Total events	38		11			
Heterogeneity: Chi² = 0.16, df = 1 (P = 0.69); I² = 0%						
Test for overall effect: Z = 0.11 (P = 0.91)						
**Total (95% CI)**		**1048**		**318**	**100.0%**	**1.06 [0.61, 1.83]**
Total events	53		15			
Heterogeneity: Chi² = 0.32, df = 3 (P = 0.96); I² = 0%						
Test for overall effect: Z = 0.19 (P = 0.85)						
Test for subgroup differences: Chi² = 0.01, df = 1 (P = 0.92), I² = 0%						

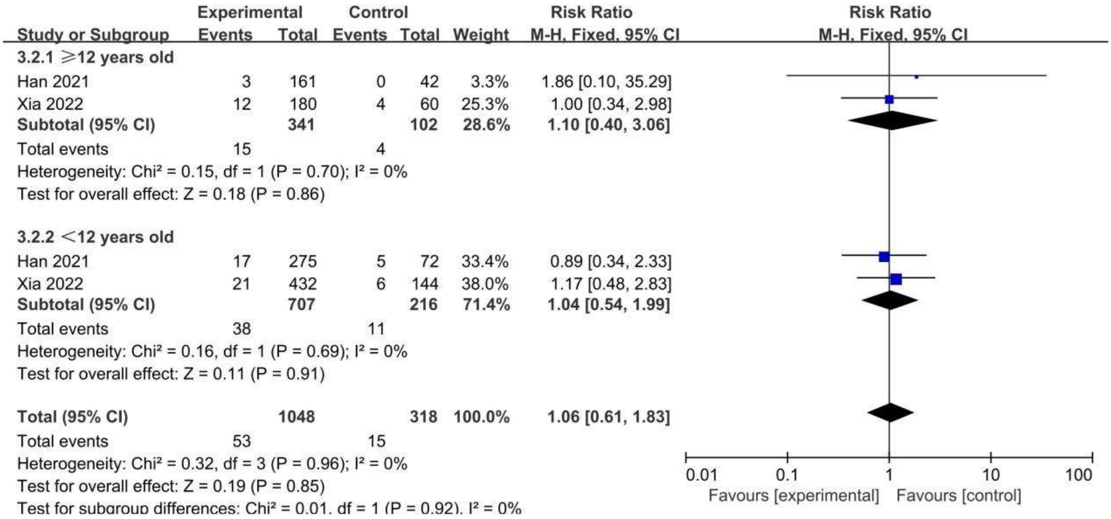

E) Headache

Study or Subgroup	Experimental Events	Experimental Total	Control Events	Control Total	Weight	Risk Ratio M-H, Fixed, 95% CI
**3.2.1 ⩾12 years old**						
Han 2021	8	161	2	42	41.0%	1.04 [0.23, 4.73]
Xia 2022	1	180	0	60	9.7%	1.01 [0.04, 24.49]
**Subtotal (95% CI)**		**341**		**102**	**50.6%**	**1.04 [0.26, 4.07]**
Total events	9		2			
Heterogeneity: Chi² = 0.00, df = 1 (P = 0.99); I² = 0%						
Test for overall effect: Z = 0.05 (P = 0.96)						
**3.2.2 <12 years old**						
Han 2021	2	275	1	72	20.5%	0.52 [0.05, 5.69]
Xia 2022	0	126	1	42	28.9%	0.11 [0.00, 2.72]
**Subtotal (95% CI)**		**401**		**114**	**49.4%**	**0.28 [0.05, 1.69]**
Total events	2		2			
Heterogeneity: Chi² = 0.58, df = 1 (P = 0.45); I² = 0%						
Test for overall effect: Z = 1.38 (P = 0.17)						
**Total (95% CI)**		**742**		**216**	**100.0%**	**0.66 [0.24, 1.88]**
Total events	11		4			
Heterogeneity: Chi² = 1.64, df = 3 (P = 0.65); I² = 0%						
Test for overall effect: Z = 0.77 (P = 0.44)						
Test for subgroup differences: Chi² = 1.28, df = 1 (P = 0.26), I² = 21.9%						

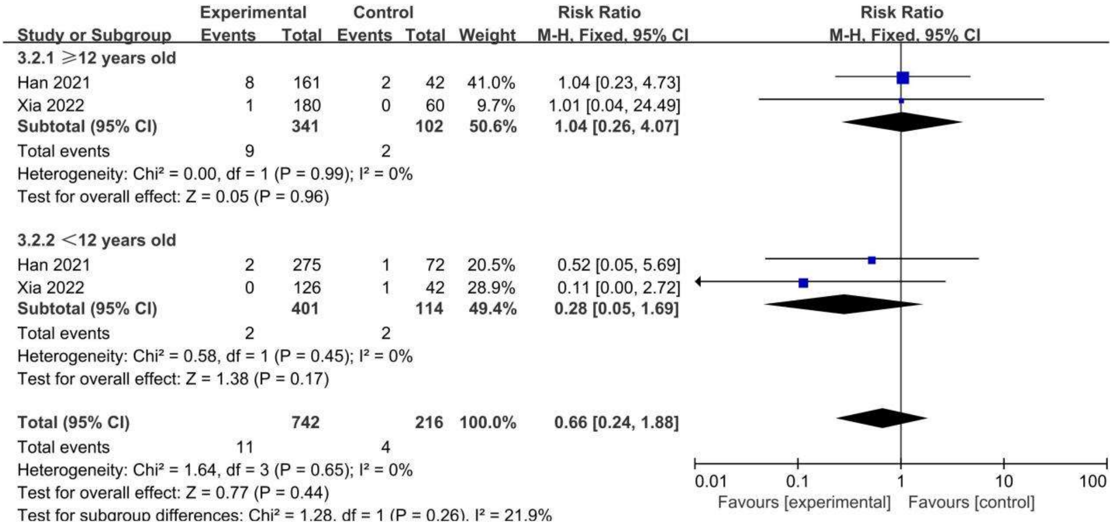

F) Vomiting

Study or Subgroup	Experimental Events	Experimental Total	Control Events	Control Total	Weight	Risk Ratio M-H, Fixed, 95% CI
**3.2.1 ⩾12 years old**						
Han 2021	2	161	0	42	25.3%	1.33 [0.06, 27.13]
Xia 2022	0	54	0	18		Not estimable
**Subtotal (95% CI)**		**215**		**60**	**25.3%**	**1.33 [0.06, 27.13]**
Total events	2		0			
Heterogeneity: Not applicable						
Test for overall effect: Z = 0.18 (P = 0.85)						
**3.2.2 <12 years old**						
Han 2021	2	275	1	72	50.8%	0.52 [0.05, 5.69]
Xia 2022	1	126	0	42	23.9%	1.02 [0.04, 24.47]
**Subtotal (95% CI)**		**401**		**114**	**74.7%**	**0.68 [0.10, 4.51]**
Total events	3		1			
Heterogeneity: Chi² = 0.11, df = 1 (P = 0.74); I² = 0%						
Test for overall effect: Z = 0.40 (P = 0.69)						
**Total (95% CI)**		**616**		**174**	**100.0%**	**0.84 [0.17, 4.14]**
Total events	5		1			
Heterogeneity: Chi² = 0.25, df = 2 (P = 0.88); I² = 0%						
Test for overall effect: Z = 0.21 (P = 0.84)						
Test for subgroup differences: Chi² = 0.13, df = 1 (P = 0.71), I² = 0%						

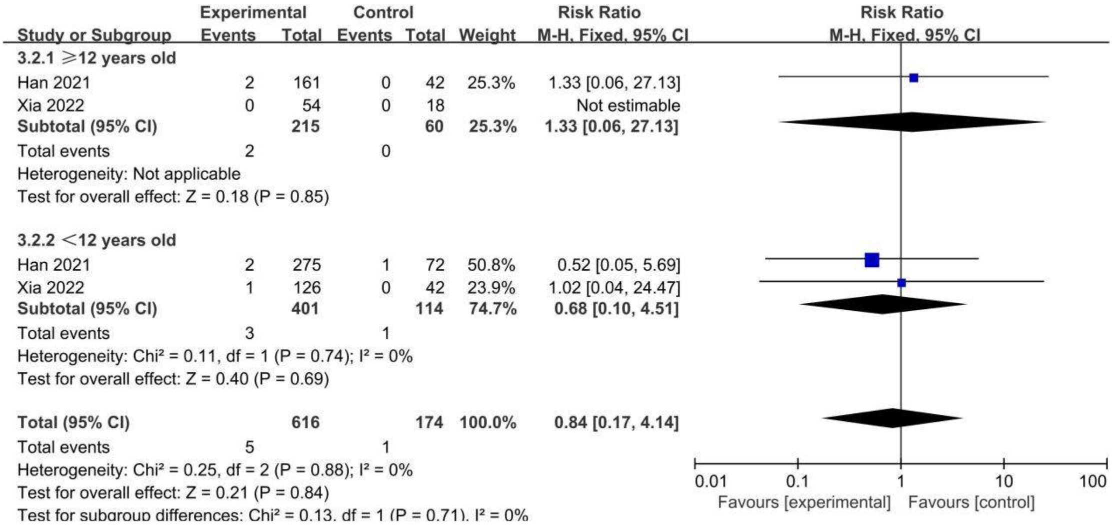

G) Diarrhea

Study or Subgroup	Experimental Events	Experimental Total	Control Events	Control Total	Weight	Risk Ratio M-H, Fixed, 95% CI
**3.2.1 ≥12 years old**						
Han 2021	2	161	1	42	17.0%	0.52 [0.05, 5.62]
Xia 2022	1	180	1	60	16.1%	0.33 [0.02, 5.25]
**Subtotal (95% CI)**		**341**		**102**	**33.0%**	**0.43 [0.07, 2.57]**
Total events	3		2			
Heterogeneity: Chi² = 0.06, df = 1 (P = 0.81); I² = 0%						
Test for overall effect: Z = 0.92 (P = 0.36)						
**3.2.2 <12 years old**						
Han 2021	2	275	3	72	50.9%	0.17 [0.03, 1.03]
Xia 2022	1	306	1	102	16.1%	0.33 [0.02, 5.28]
**Subtotal (95% CI)**		**581**		**174**	**67.0%**	**0.21 [0.05, 0.93]**
Total events	3		4			
Heterogeneity: Chi² = 0.15, df = 1 (P = 0.70); I² = 0%						
Test for overall effect: Z = 2.06 (P = 0.04)						
**Total (95% CI)**		**922**		**276**	**100.0%**	**0.28 [0.09, 0.87]**
Total events	6		6			
Heterogeneity: Chi² = 0.57, df = 3 (P = 0.90); I² = 0%						
Test for overall effect: Z = 2.20 (P = 0.03)						
Test for subgroup differences: Chi² = 0.35, df = 1 (P = 0.55), I² = 0%						

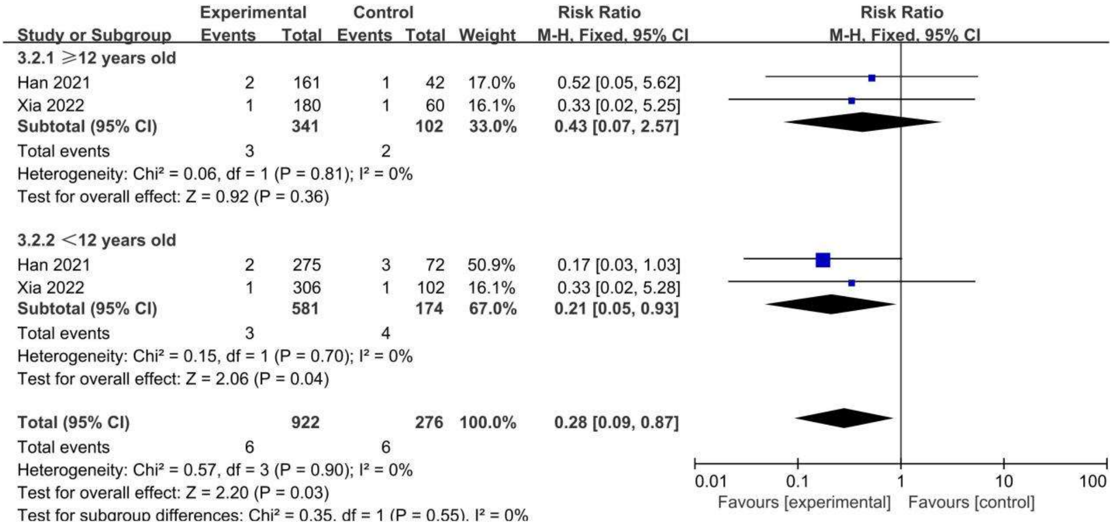

Figure S13. Specific adverse reactions in the inactivated vaccine recipients aged 12 -17 years versus 3 - 12 years:

	12-17 years old		3-12 years old			Risk Ratio
Study or Subgroup	Events	Total	Events	Total	Weight	M-H, Fixed, 95% CI
**3.5.1 Local pain（local adverse reactions）**						
Han 2021	46	161	25	275	26.9%	3.14 [2.01, 4.91]
Xia 2022	23	180	23	432	19.7%	2.40 [1.38, 4.16]
**Subtotal (95% CI)**		**341**		**707**	**46.6%**	**2.83 [2.00, 4.00]**
Total events	69		48			
Heterogeneity: Chi² = 0.56, df = 1 (P = 0.46); I² = 0%						
Test for overall effect: Z = 5.89 (P < 0.00001)						
**3.5.2 Erythema / Redness（local adverse reactions）**						
Han 2021	1	161	0	275	0.5%	5.11 [0.21, 124.73]
Xia 2022	0	54	7	432	2.5%	0.52 [0.03, 9.06]
**Subtotal (95% CI)**		**215**		**707**	**3.0%**	**1.35 [0.21, 8.46]**
Total events	1		7			
Heterogeneity: Chi² = 1.09, df = 1 (P = 0.30); I² = 8%						
Test for overall effect: Z = 0.32 (P = 0.75)						
**3.5.3 Swelling / Hardness（local adverse reactions）**						
Han 2021	6	161	3	275	3.2%	3.42 [0.87, 13.47]
Xia 2022	0	54	3	432	1.1%	1.12 [0.06, 21.49]
**Subtotal (95% CI)**		**215**		**707**	**4.4%**	**2.81 [0.83, 9.51]**
Total events	6		6			
Heterogeneity: Chi² = 0.45, df = 1 (P = 0.50); I² = 0%						
Test for overall effect: Z = 1.67 (P = 0.10)						
**3.5.4 Fever (systemic adverse reactions)**						
Han 2021	3	161	17	275	18.3%	0.30 [0.09, 1.01]
Xia 2022	12	180	21	432	18.0%	1.37 [0.69, 2.73]
**Subtotal (95% CI)**		**341**		**707**	**36.3%**	**0.83 [0.47, 1.47]**
Total events	15		38			
Heterogeneity: Chi² = 4.73, df = 1 (P = 0.03); I² = 79%						
Test for overall effect: Z = 0.63 (P = 0.53)						
**3.5.5 Headache (systemic adverse reactions)**						
Han 2021	8	161	2	275	2.2%	6.83 [1.47, 31.78]
Xia 2022	1	180	0	126	0.9%	2.10 [0.09, 51.26]
**Subtotal (95% CI)**		**341**		**401**	**3.0%**	**5.49 [1.42, 21.17]**
Total events	9		2			
Heterogeneity: Chi² = 0.42, df = 1 (P = 0.51); I² = 0%						
Test for overall effect: Z = 2.47 (P = 0.01)						
**3.5.6 vomiting (systemic adverse reactions)**						
Han 2021	2	161	2	275	2.2%	1.71 [0.24, 12.01]
Xia 2022	0	54	1	126	1.3%	0.77 [0.03, 18.60]
**Subtotal (95% CI)**		**215**		**401**	**3.5%**	**1.35 [0.26, 6.95]**
Total events	2		3			
Heterogeneity: Chi² = 0.18, df = 1 (P = 0.68); I² = 0%						
Test for overall effect: Z = 0.36 (P = 0.72)						
**3.5.7 Diarrhea (systemic adverse reactions)**						
Han 2021	2	161	2	275	2.2%	1.71 [0.24, 12.01]
Xia 2022	1	180	1	306	1.1%	1.70 [0.11, 27.01]
**Subtotal (95% CI)**		**341**		**581**	**3.2%**	**1.71 [0.35, 8.40]**
Total events	3		3			
Heterogeneity: Chi² = 0.00, df = 1 (P = 1.00); I² = 0%						
Test for overall effect: Z = 0.66 (P = 0.51)						
**Total (95% CI)**		**2009**		**4211**	**100.0%**	**2.05 [1.58, 2.66]**
Total events	105		107			
Heterogeneity: Chi² = 19.44, df = 13 (P = 0.11); I² = 33%						
Test for overall effect: Z = 5.40 (P < 0.00001)						
Test for subgroup differences: Chi² = 15.65, df = 6 (P = 0.02), I² = 61.7%						

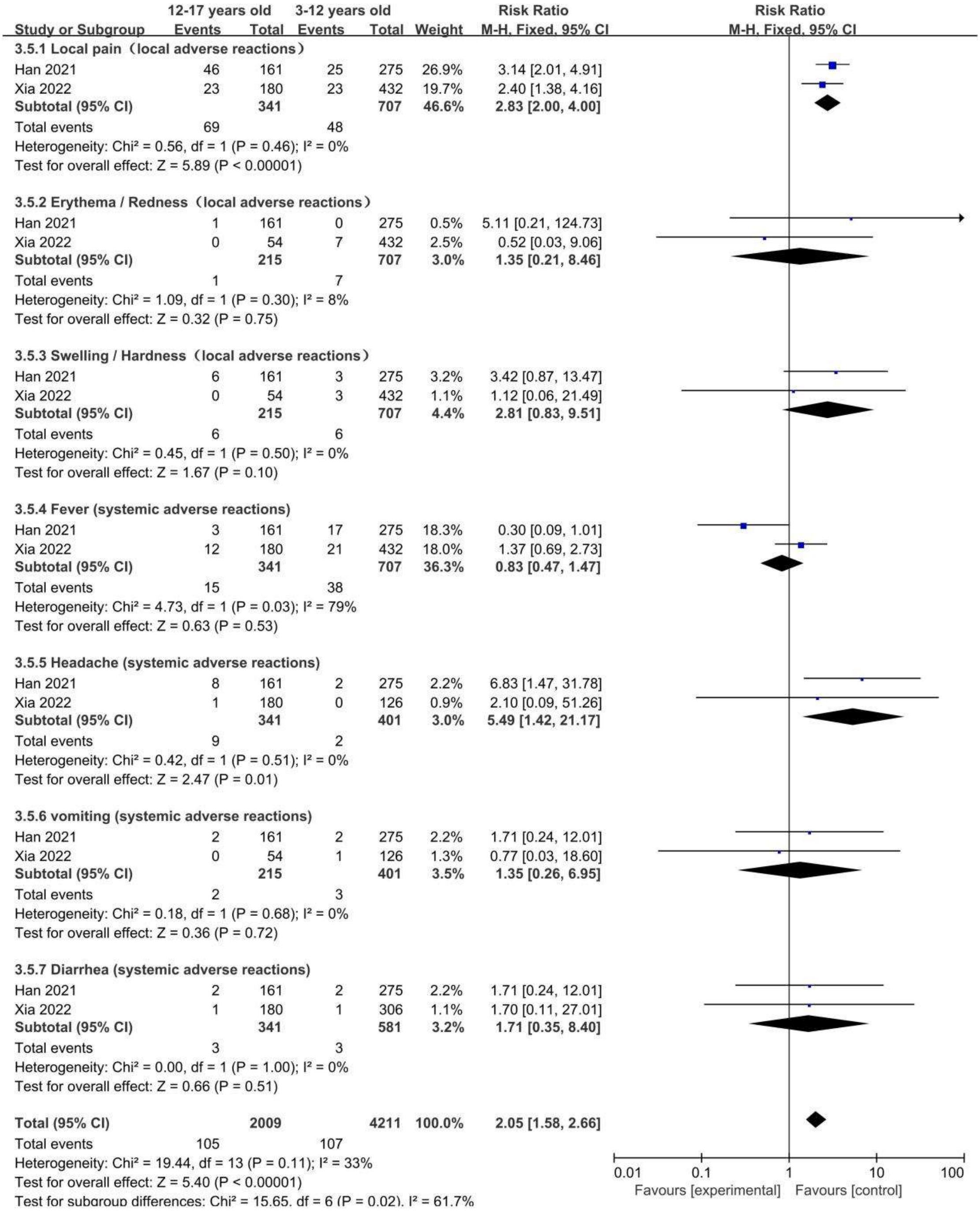

Figure S14. Adverse reactions in the subunit vaccine group within 7 days of different ages versus the control group after whole vaccination:

A) Local pain

Study or Subgroup	Experimental Events	Experimental Total	Control Events	Control Total	Weight	Risk Ratio M-H, Fixed, 95% CI
**3.3.1 ⩾12 years old**						
Áñez 2022	648	1448	126	726	66.8%	2.58 [2.18, 3.05]
Liu 2022	238	341	19	58	12.9%	2.13 [1.46, 3.10]
Thuluva 2022	38	234	17	78	10.1%	0.75 [0.45, 1.24]
**Subtotal (95% CI)**		**2023**		**862**	**89.9%**	**2.31 [1.99, 2.67]**
Total events	924		162			
Heterogeneity: Chi² = 20.62, df = 2 (P < 0.0001); I² = 90%						
Test for overall effect: Z = 11.21 (P < 0.00001)						
**3.3.2 <12 years old**						
Thuluva 2022	30	234	17	78	10.1%	0.59 [0.34, 1.01]
**Subtotal (95% CI)**		**234**		**78**	**10.1%**	**0.59 [0.34, 1.01]**
Total events	30		17			
Heterogeneity: Not applicable						
Test for overall effect: Z = 1.94 (P = 0.05)						
**Total (95% CI)**		**2257**		**940**	**100.0%**	**2.13 [1.85, 2.45]**
Total events	954		179			
Heterogeneity: Chi² = 43.22, df = 3 (P < 0.00001); I² = 93%						
Test for overall effect: Z = 10.64 (P < 0.00001)						
Test for subgroup differences: Chi² = 23.17, df = 1 (P < 0.00001), I² = 95.7%						

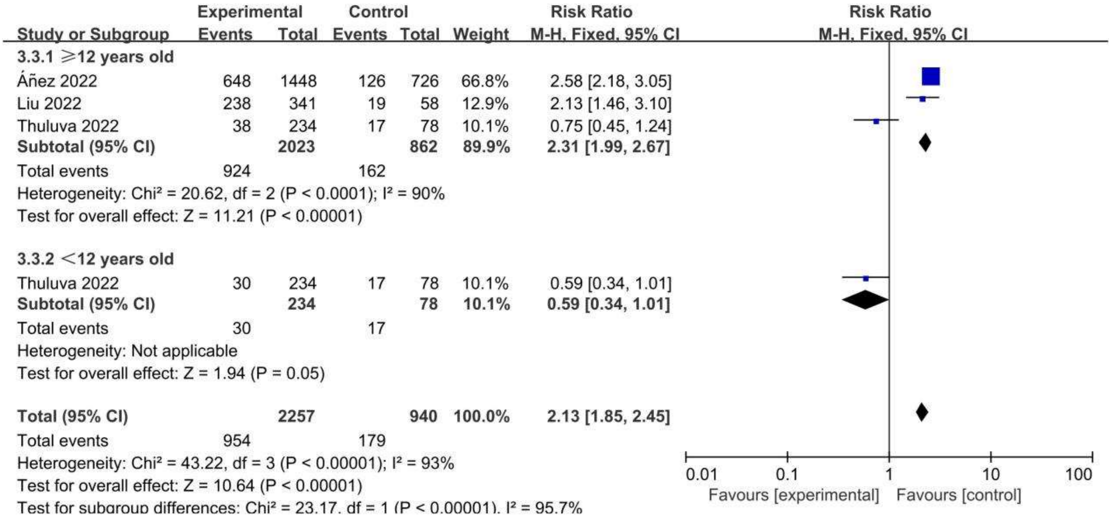

B) Erythema or Redness

Study or Subgroup	Experimental Events	Experimental Total	Control Events	Control Total	Weight	Risk Ratio M-H, Fixed, 95% CI
**3.3.1 ⩾12 years old**						
Áñez 2022	15	1448	5	726	31.7%	1.50 [0.55, 4.12]
Liu 2022	4	341	0	58	4.1%	1.55 [0.08, 28.46]
Thuluva 2022	6	234	3	78	21.4%	0.67 [0.17, 2.60]
**Subtotal (95% CI)**		**2023**		**862**	**57.2%**	**1.19 [0.55, 2.58]**
Total events	25		8			
Heterogeneity: Chi² = 0.94, df = 2 (P = 0.63); I² = 0%						
Test for overall effect: Z = 0.45 (P = 0.65)						
**3.3.2 <12 years old**						
Thuluva 2022	8	234	6	78	42.8%	0.44 [0.16, 1.24]
**Subtotal (95% CI)**		**234**		**78**	**42.8%**	**0.44 [0.16, 1.24]**
Total events	8		6			
Heterogeneity: Not applicable						
Test for overall effect: Z = 1.55 (P = 0.12)						
**Total (95% CI)**		**2257**		**940**	**100.0%**	**0.87 [0.48, 1.60]**
Total events	33		14			
Heterogeneity: Chi² = 3.08, df = 3 (P = 0.38); I² = 3%						
Test for overall effect: Z = 0.44 (P = 0.66)						
Test for subgroup differences: Chi² = 2.27, df = 1 (P = 0.13), I² = 56.0%						

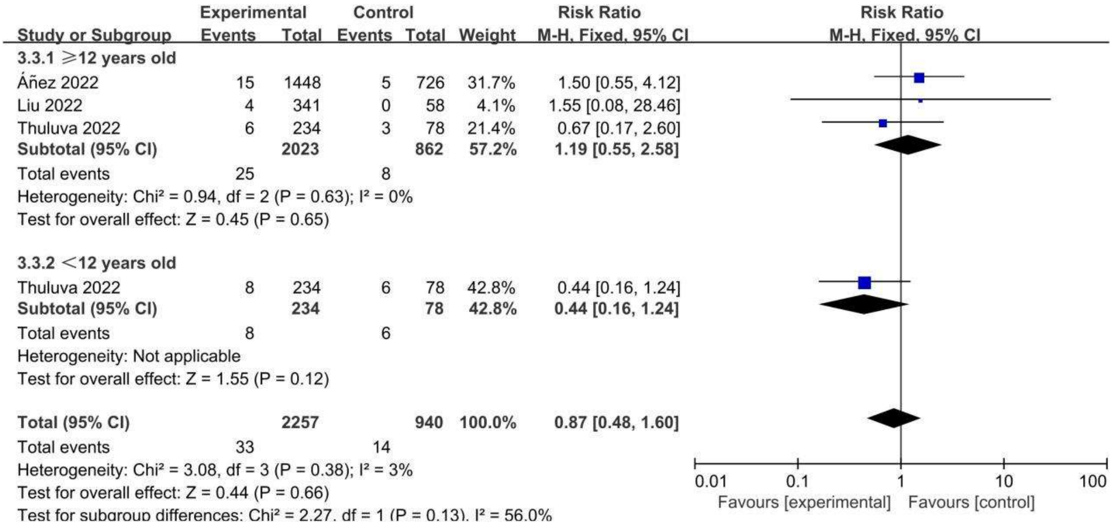

C) Swelling or Hardness

Study or Subgroup	Experimental Events	Experimental Total	Control Events	Control Total	Weight	Risk Ratio M-H, Fixed, 95% CI
**3.3.1 ⩾12 years old**						
Áñez 2022	20	1448	3	726	32.4%	3.34 [1.00, 11.21]
Liu 2022	11	341	0	58	6.9%	3.97 [0.24, 66.43]
Thuluva 2022	6	234	1	78	12.1%	2.00 [0.24, 16.36]
**Subtotal (95% CI)**		**2023**		**862**	**51.4%**	**3.11 [1.16, 8.30]**
Total events	37		4			
Heterogeneity: Chi² = 0.21, df = 2 (P = 0.90); I² = 0%						
Test for overall effect: Z = 2.26 (P = 0.02)						
**3.3.2 <12 years old**						
Thuluva 2022	4	234	4	78	48.6%	0.33 [0.09, 1.30]
**Subtotal (95% CI)**		**234**		**78**	**48.6%**	**0.33 [0.09, 1.30]**
Total events	4		4			
Heterogeneity: Not applicable						
Test for overall effect: Z = 1.58 (P = 0.11)						
**Total (95% CI)**		**2257**		**940**	**100.0%**	**1.76 [0.85, 3.65]**
Total events	41		8			
Heterogeneity: Chi² = 7.15, df = 3 (P = 0.07); I² = 58%						
Test for overall effect: Z = 1.52 (P = 0.13)						
Test for subgroup differences: Chi² = 6.79, df = 1 (P = 0.009), I² = 85.3%						

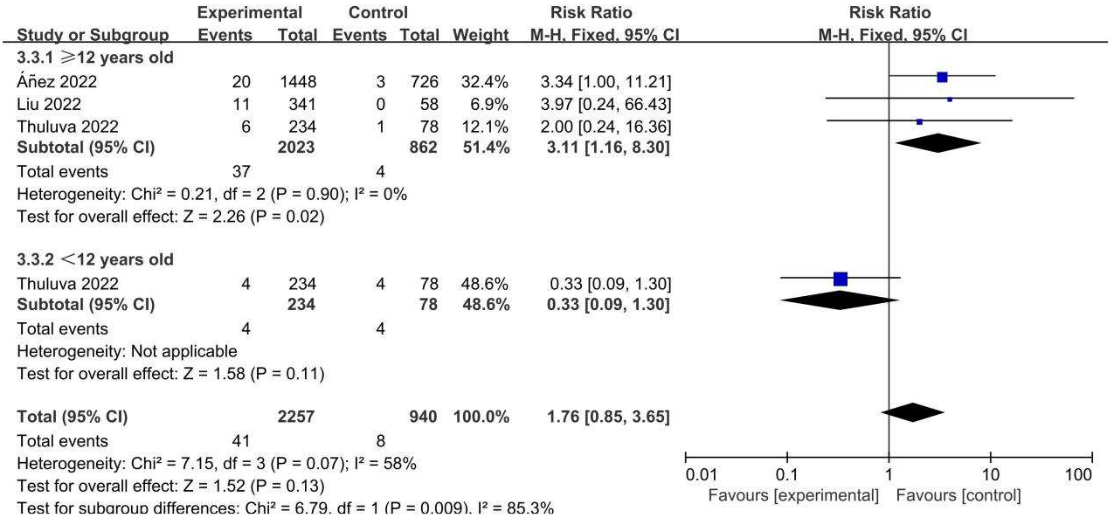

D) Headache

Study or Subgroup	Experimental Events	Experimental Total	Control Events	Control Total	Weight	Risk Ratio M-H, Fixed, 95% CI
**3.3.1 ⩾12 years old**						
Áñez 2022	440	1448	181	726	88.5%	1.22 [1.05, 1.41]
Liu 2022	58	341	13	58	8.2%	0.76 [0.45, 1.29]
Thuluva 2022	8	234	4	78	2.2%	0.67 [0.21, 2.15]
**Subtotal (95% CI)**		**2023**		**862**	**98.9%**	**1.17 [1.01, 1.35]**
Total events	506		198			
Heterogeneity: Chi² = 3.71, df = 2 (P = 0.16); I² = 46%						
Test for overall effect: Z = 2.16 (P = 0.03)						
**3.3.2 <12 years old**						
Thuluva 2022	8	234	2	78	1.1%	1.33 [0.29, 6.15]
**Subtotal (95% CI)**		**234**		**78**	**1.1%**	**1.33 [0.29, 6.15]**
Total events	8		2			
Heterogeneity: Not applicable						
Test for overall effect: Z = 0.37 (P = 0.71)						
**Total (95% CI)**		**2257**		**940**	**100.0%**	**1.17 [1.02, 1.35]**
Total events	514		200			
Heterogeneity: Chi² = 3.74, df = 3 (P = 0.29); I² = 20%						
Test for overall effect: Z = 2.19 (P = 0.03)						
Test for subgroup differences: Chi² = 0.03, df = 1 (P = 0.87), I² = 0%						

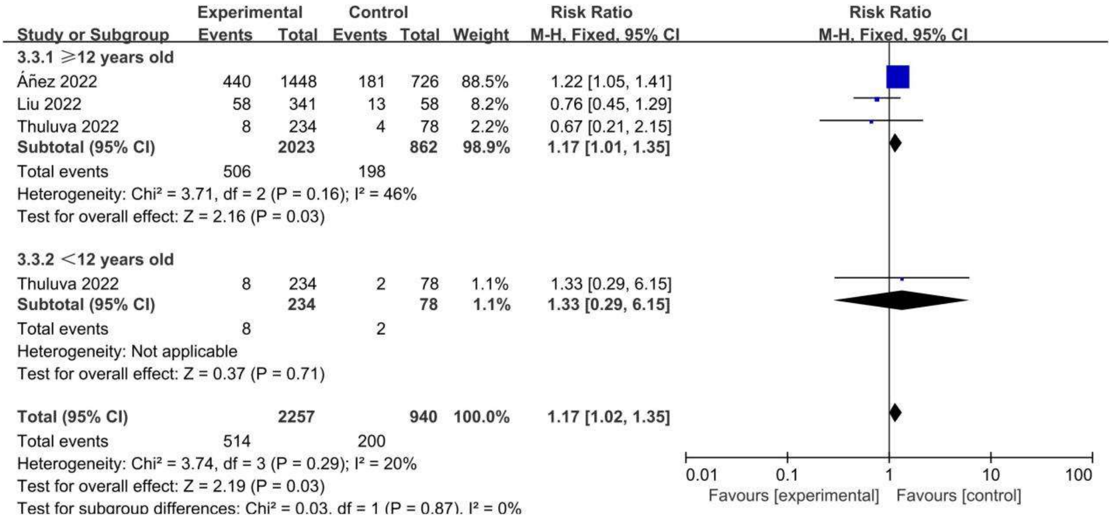

E) Fatigue

Study or Subgroup	Experimental Events	Experimental Total	Control Events	Control Total	Weight	Risk Ratio M-H, Fixed, 95% CI
**3.3.1 ≥12 years old**						
Áñez 2022	350	1448	113	726	85.5%	1.55 [1.28, 1.88]
Liu 2022	105	341	14	58	13.6%	1.28 [0.79, 2.07]
Thuluva 2022	1	234	0	78	0.4%	1.01 [0.04, 24.51]
**Subtotal (95% CI)**		**2023**		**862**	**99.6%**	**1.51 [1.27, 1.81]**
Total events	456		127			
Heterogeneity: Chi² = 0.61, df = 2 (P = 0.74); I² = 0%						
Test for overall effect: Z = 4.55 (P < 0.00001)						
**3.3.2 <12 years old**						
Thuluva 2022	3	234	0	78	0.4%	2.35 [0.12, 45.06]
**Subtotal (95% CI)**		**234**		**78**	**0.4%**	**2.35 [0.12, 45.06]**
Total events	3		0			
Heterogeneity: Not applicable						
Test for overall effect: Z = 0.57 (P = 0.57)						
**Total (95% CI)**		**2257**		**940**	**100.0%**	**1.52 [1.27, 1.81]**
Total events	459		127			
Heterogeneity: Chi² = 0.70, df = 3 (P = 0.87); I² = 0%						
Test for overall effect: Z = 4.58 (P < 0.00001)						
Test for subgroup differences: Chi² = 0.09, df = 1 (P = 0.77), I² = 0%						

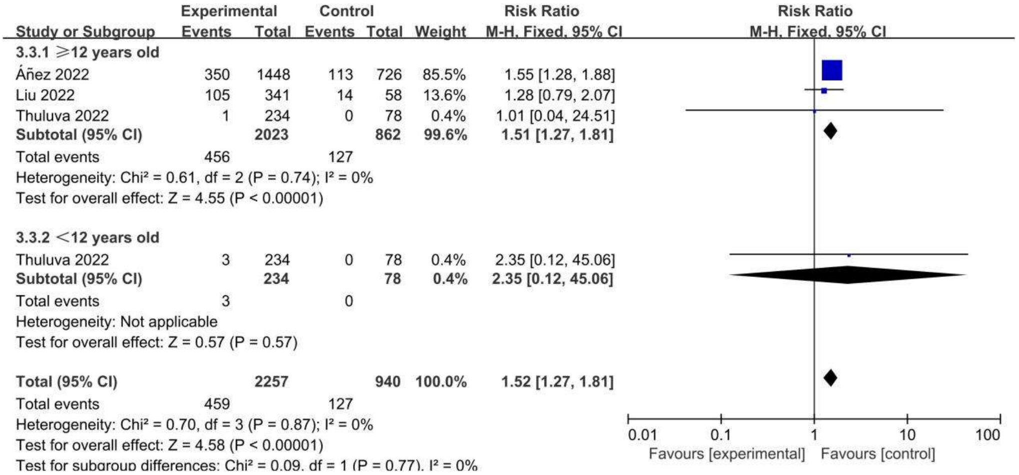

F) Myalgia

Study or Subgroup	Experimental Events	Experimental Total	Control Events	Control Total	Weight	Risk Ratio M-H, Fixed, 95% CI
**3.3.1 ≥12 years old**						
Áñez 2022	492	1448	114	726	86.5%	2.16 [1.80, 2.60]
Liu 2022	81	341	13	58	12.7%	1.06 [0.63, 1.77]
Thuluva 2022	5	234	0	78	0.4%	3.70 [0.21, 66.13]
**Subtotal (95% CI)**		**2023**		**862**	**99.6%**	**2.03 [1.71, 2.41]**
Total events	578		127			
Heterogeneity: Chi² = 6.75, df = 2 (P = 0.03); I² = 70%						
Test for overall effect: Z = 8.08 (P < 0.00001)						
**3.3.2 <12 years old**						
Thuluva 2022	1	234	0	78	0.4%	1.01 [0.04, 24.51]
**Subtotal (95% CI)**		**234**		**78**	**0.4%**	**1.01 [0.04, 24.51]**
Total events	1		0			
Heterogeneity: Not applicable						
Test for overall effect: Z = 0.01 (P = 1.00)						
**Total (95% CI)**		**2257**		**940**	**100.0%**	**2.03 [1.71, 2.40]**
Total events	579		127			
Heterogeneity: Chi² = 6.92, df = 3 (P = 0.07); I² = 57%						
Test for overall effect: Z = 8.07 (P < 0.00001)						
Test for subgroup differences: Chi² = 0.18, df = 1 (P = 0.67), I² = 0%						

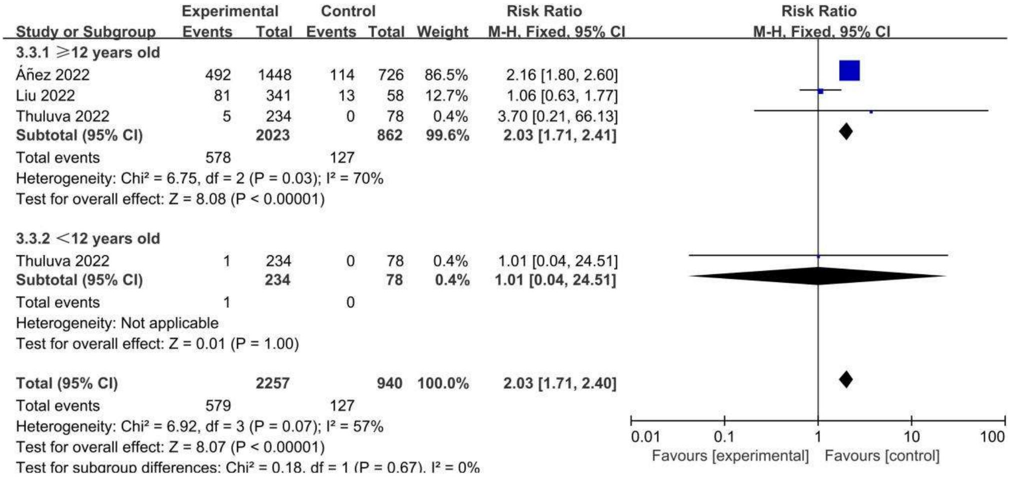

G) Nausea / Vomiting

Study or Subgroup	Experimental Events	Experimental Total	Control Events	Control Total	Weight	Risk Ratio M-H, Fixed, 95% CI
**3.3.1 ≥12 years old**						
Áñez 2022	113	1448	56	726	87.4%	1.01 [0.74, 1.38]
Liu 2022	22	341	5	58	10.0%	0.75 [0.30, 1.90]
Thuluva 2022	3	234	0	78	0.9%	2.35 [0.12, 45.06]
**Subtotal (95% CI)**		**2023**		**862**	**98.2%**	**1.00 [0.75, 1.33]**
Total events	138		61			
Heterogeneity: Chi² = 0.70, df = 2 (P = 0.71); I² = 0%						
Test for overall effect: Z = 0.02 (P = 0.98)						
**3.3.2 <12 years old**						
Thuluva 2022	1	234	1	78	1.8%	0.33 [0.02, 5.27]
**Subtotal (95% CI)**		**234**		**78**	**1.8%**	**0.33 [0.02, 5.27]**
Total events	1		1			
Heterogeneity: Not applicable						
Test for overall effect: Z = 0.78 (P = 0.44)						
**Total (95% CI)**		**2257**		**940**	**100.0%**	**0.99 [0.74, 1.31]**
Total events	139		62			
Heterogeneity: Chi² = 1.29, df = 3 (P = 0.73); I² = 0%						
Test for overall effect: Z = 0.10 (P = 0.92)						
Test for subgroup differences: Chi² = 0.60, df = 1 (P = 0.44), I² = 0%						

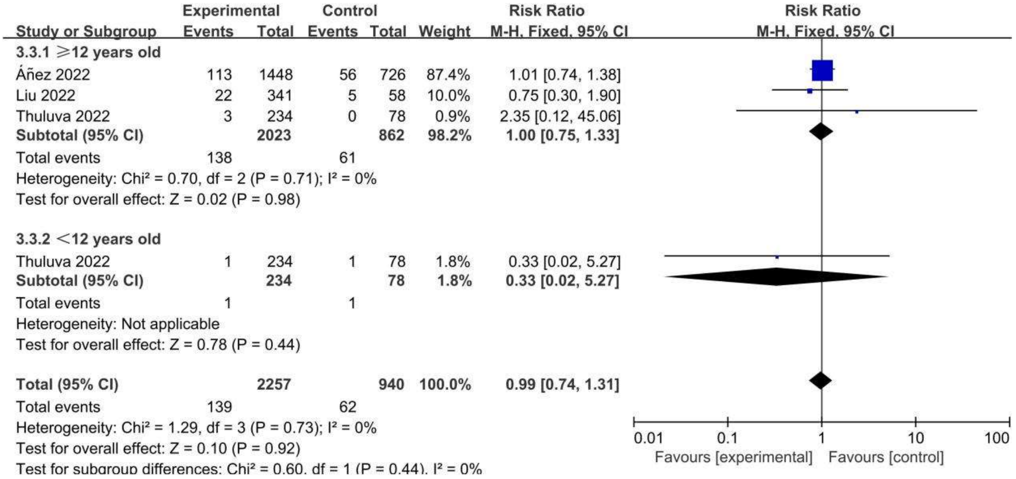

Figure S15. Specific adverse reactions in the subunit vaccine recipients aged 12 -17 years versus 5- 11 years:

	12-17 years old		5-11 years old			Risk Ratio	Risk Ratio
**Study or Subgroup**	**Events**	**Total**	**Events**	**Total**	**Weight**	**M-H, Fixed, 95% CI**	**M-H, Fixed, 95% CI**
**3.4.1 Local pain**							
Thuluva 2022	38	234	30	234	54.5%	1.27 [0.81, 1.97]	
**Subtotal (95% CI)**		**234**		**234**	**54.5%**	**1.27 [0.81, 1.97]**	
Total events	38		30				
Heterogeneity: Not applicable							
Test for overall effect: Z = 1.05 (P = 0.30)							
**3.4.2 Erythema / Redness**							
Thuluva 2022	6	234	8	234	14.5%	0.75 [0.26, 2.13]	
**Subtotal (95% CI)**		**234**		**234**	**14.5%**	**0.75 [0.26, 2.13]**	
Total events	6		8				
Heterogeneity: Not applicable							
Test for overall effect: Z = 0.54 (P = 0.59)							
**3.4.3 Swelling / Hardness**							
Thuluva 2022	6	234	4	234	7.3%	1.50 [0.43, 5.25]	
**Subtotal (95% CI)**		**234**		**234**	**7.3%**	**1.50 [0.43, 5.25]**	
Total events	6		4				
Heterogeneity: Not applicable							
Test for overall effect: Z = 0.63 (P = 0.53)							
**3.4.4 Headache**							
Thuluva 2022	8	234	8	234	14.5%	1.00 [0.38, 2.62]	
**Subtotal (95% CI)**		**234**		**234**	**14.5%**	**1.00 [0.38, 2.62]**	
Total events	8		8				
Heterogeneity: Not applicable							
Test for overall effect: Z = 0.00 (P = 1.00)							
**3.4.5 Fatigue**							
Thuluva 2022	1	234	3	234	5.5%	0.33 [0.03, 3.18]	
**Subtotal (95% CI)**		**234**		**234**	**5.5%**	**0.33 [0.03, 3.18]**	
Total events	1		3				
Heterogeneity: Not applicable							
Test for overall effect: Z = 0.95 (P = 0.34)							
**3.4.6 Myalgia**							
Thuluva 2022	5	234	1	234	1.8%	5.00 [0.59, 42.47]	
**Subtotal (95% CI)**		**234**		**234**	**1.8%**	**5.00 [0.59, 42.47]**	
Total events	5		1				
Heterogeneity: Not applicable							
Test for overall effect: Z = 1.47 (P = 0.14)							
**3.4.7 Nausea / Vomiting**							
Thuluva 2022	3	234	1	234	1.8%	3.00 [0.31, 28.63]	
**Subtotal (95% CI)**		**234**		**234**	**1.8%**	**3.00 [0.31, 28.63]**	
Total events	3		1				
Heterogeneity: Not applicable							
Test for overall effect: Z = 0.95 (P = 0.34)							
**Total (95% CI)**		**1638**		**1638**	**100.0%**	**1.22 [0.87, 1.71]**	
Total events	67		55				
Heterogeneity: Chi² = 4.68, df = 6 (P = 0.59); I² = 0%							
Test for overall effect: Z = 1.14 (P = 0.26)							
Test for subgroup differences: Chi² = 4.68, df = 6 (P = 0.59), I² = 0%							

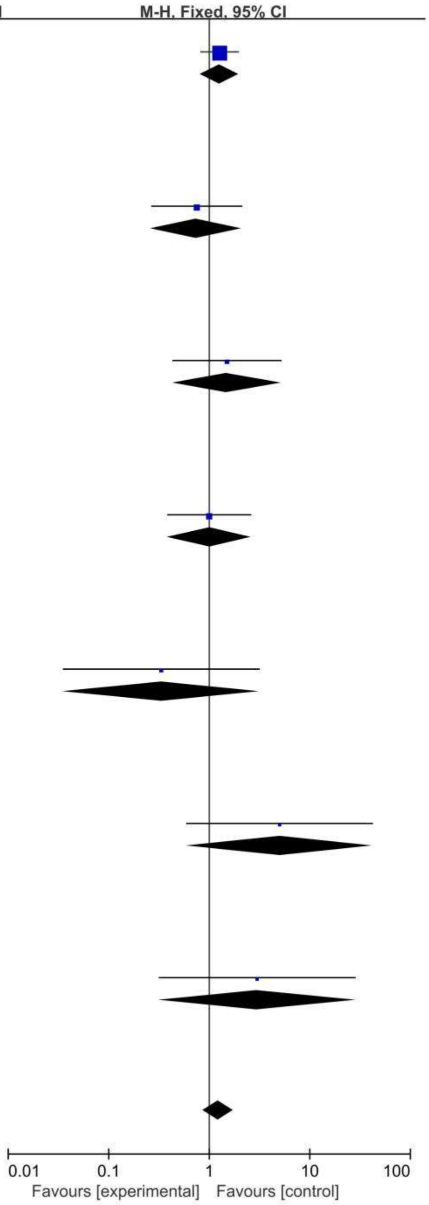

Figure S16. **Seroconversion rate in the vaccine group versus the control group: A) Neutralizing antibody in the inactivated vaccine group; B) Neutralizing antibody in the subunit vaccine group; C) Neutralizing antibody in the adenovirus vaccine group; D) RBD − binding enzyme-linked immunosorbent assay antibody; E) Neutralizing antibody 28 days after dose 2 in the inactivated vaccine group; F) Neutralizing antibody 28 days after dose 3 in the inactivated vaccine group.**

A) Neutralizing antibody in the inactivated vaccine group

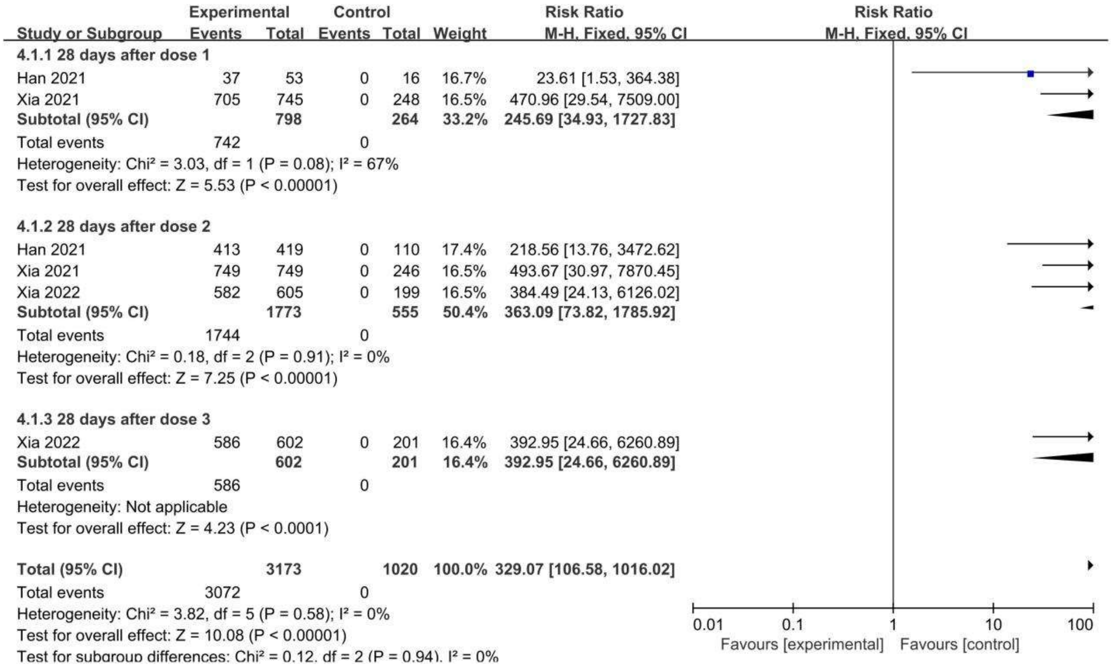

B) Neutralizing antibody in the subunit vaccine group

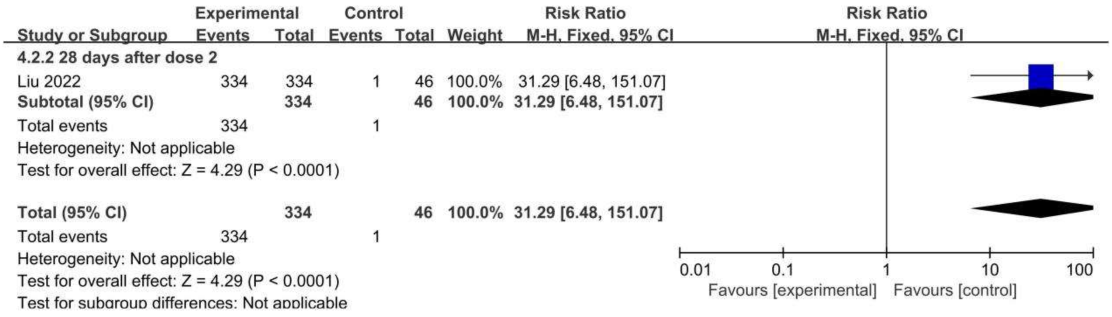

C) Neutralizing antibody in the adenovirus vaccine group

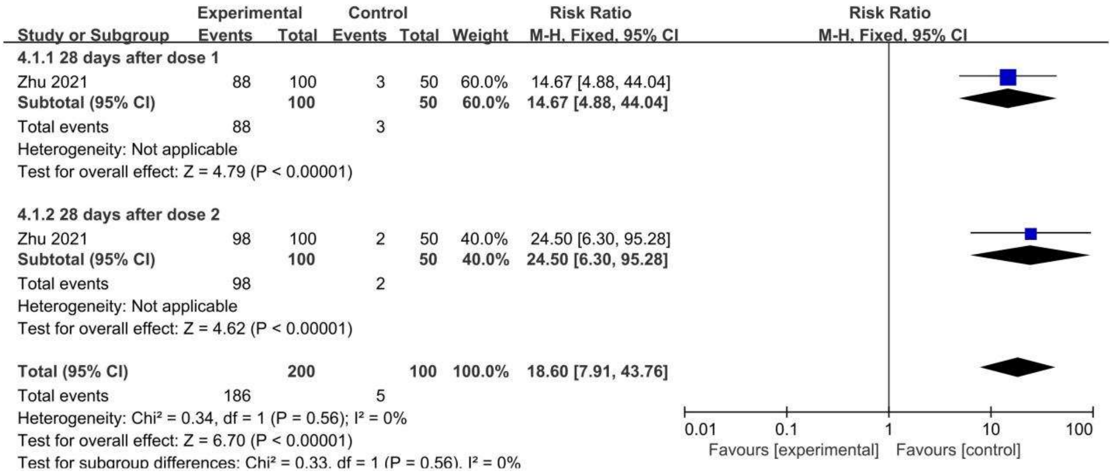

D) RBD – binding enzyme-linked immunosorbent assay antibody in the adenovirus vaccine group

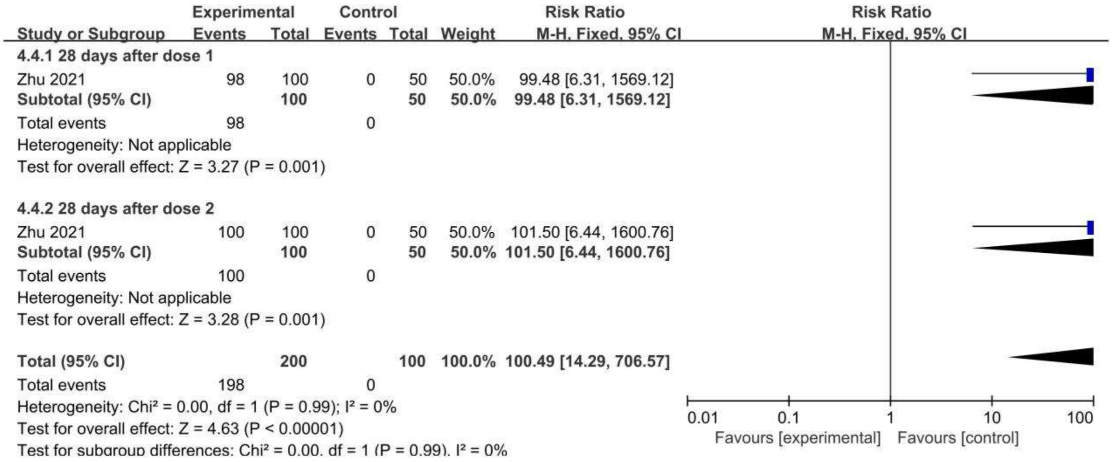

E) Neutralizing antibody 28 days after dose 2 in the inactivated vaccine group

Study or Subgroup	Experimental Events	Experimental Total	Control Events	Control Total	Weight	Risk Ratio M-H, Fixed, 95% CI
**4.2.1 3 - 5 years old**						
Han 2021	109	109	0	29	11.4%	59.73 [3.82, 933.02]
Xia 2021	248	248	0	81	11.0%	163.67 [10.32, 2594.65]
Xia 2022	243	247	0	79	11.0%	157.10 [9.91, 2490.02]
**Subtotal (95% CI)**		**604**		**189**	**33.4%**	**125.90 [25.72, 616.35]**
Total events	600		0			
Heterogeneity: Chi² = 0.34, df = 2 (P = 0.84); I² = 0%						
Test for overall effect: Z = 5.97 (P < 0.00001)						
**4.2.2 6 - 11/12 years old**						
Han 2021	154	155	0	41	11.5%	83.19 [5.29, 1308.27]
Xia 2021	250	250	0	83	10.9%	167.67 [10.57, 2658.51]
Xia 2022	176	179	0	60	10.9%	119.63 [7.57, 1891.05]
**Subtotal (95% CI)**		**584**		**184**	**33.3%**	**122.82 [25.05, 602.16]**
Total events	580		0			
Heterogeneity: Chi² = 0.13, df = 2 (P = 0.94); I² = 0%						
Test for overall effect: Z = 5.93 (P < 0.00001)						
**4.2.3 12/13 - 17 years old**						
Han 2021	150	155	0	40	11.5%	79.11 [5.03, 1243.70]
Xia 2021	251	251	0	82	11.0%	165.67 [10.45, 2626.62]
Xia 2022	163	179	0	60	10.9%	110.82 [7.01, 1752.31]
**Subtotal (95% CI)**		**585**		**182**	**33.4%**	**117.87 [24.04, 577.88]**
Total events	564		0			
Heterogeneity: Chi² = 0.14, df = 2 (P = 0.93); I² = 0%						
Test for overall effect: Z = 5.88 (P < 0.00001)						
**Total (95% CI)**		**1773**		**555**	**100.0%**	**122.20 [48.82, 305.89]**
Total events	1744		0			
Heterogeneity: Chi² = 0.61, df = 8 (P = 1.00); I² = 0%						
Test for overall effect: Z = 10.26 (P < 0.00001)						
Test for subgroup differences: Chi² = 0.00, df = 2 (P = 1.00), I² = 0%						

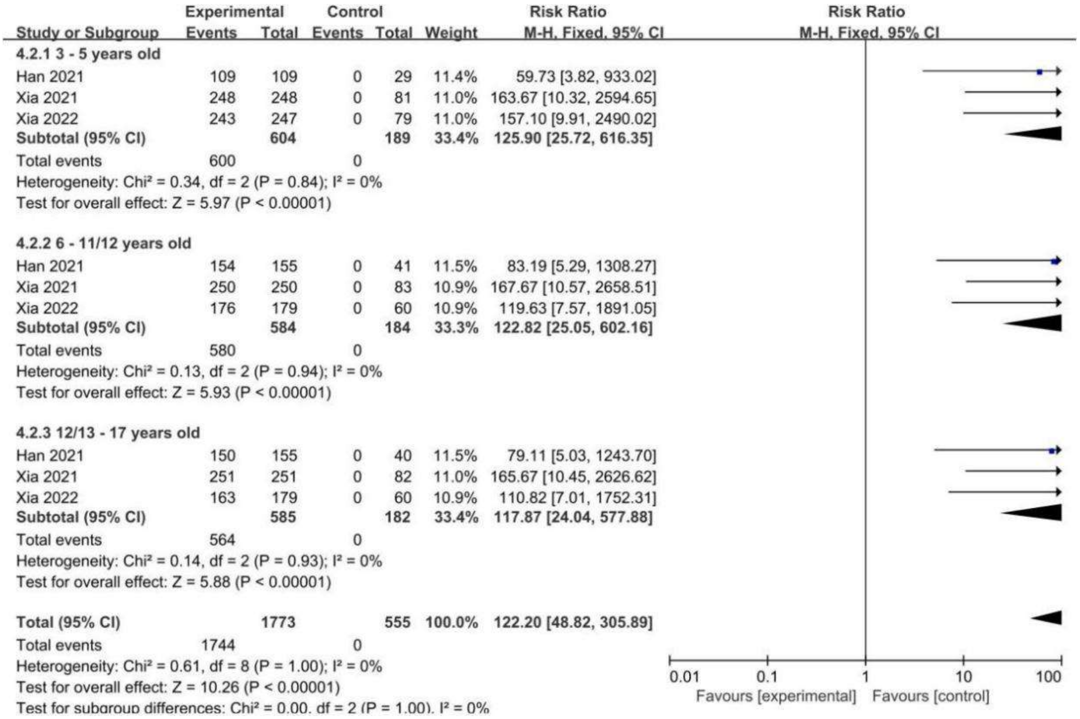

F) Neutralizing antibody 28 days after dose 3 in the inactivated vaccine group

Study or Subgroup	Experimental Events	Experimental Total	Control Events	Control Total	Weight	Risk Ratio M-H, Fixed, 95% CI
**4.3.1 3 - 5 years old**						
Xia 2022	245	245	0	81	33.5%	163.67 [10.32, 2594.58]
**Subtotal (95% CI)**		**245**		**81**	**33.5%**	**163.67 [10.32, 2594.58]**
Total events	245		0			
Heterogeneity: Not applicable						
Test for overall effect: Z = 3.62 (P = 0.0003)						
**4.3.2 6 - 12 years old**						
Xia 2022	176	178	0	60	33.3%	120.30 [7.61, 1901.57]
**Subtotal (95% CI)**		**178**		**60**	**33.3%**	**120.30 [7.61, 1901.57]**
Total events	176		0			
Heterogeneity: Not applicable						
Test for overall effect: Z = 3.40 (P = 0.0007)						
**4.3.3 13 - 17 years old**						
Xia 2022	165	178	0	60	33.3%	112.80 [7.13, 1783.53]
**Subtotal (95% CI)**		**178**		**60**	**33.3%**	**112.80 [7.13, 1783.53]**
Total events	165		0			
Heterogeneity: Not applicable						
Test for overall effect: Z = 3.35 (P = 0.0008)						
**Total (95% CI)**		**601**		**201**	**100.0%**	**132.31 [26.85, 651.98]**
Total events	586		0			
Heterogeneity: Chi² = 0.04, df = 2 (P = 0.98); I² = 0%						
Test for overall effect: Z = 6.00 (P < 0.00001)						
Test for subgroup differences: Chi² = 0.04, df = 2 (P = 0.98), I² = 0%						

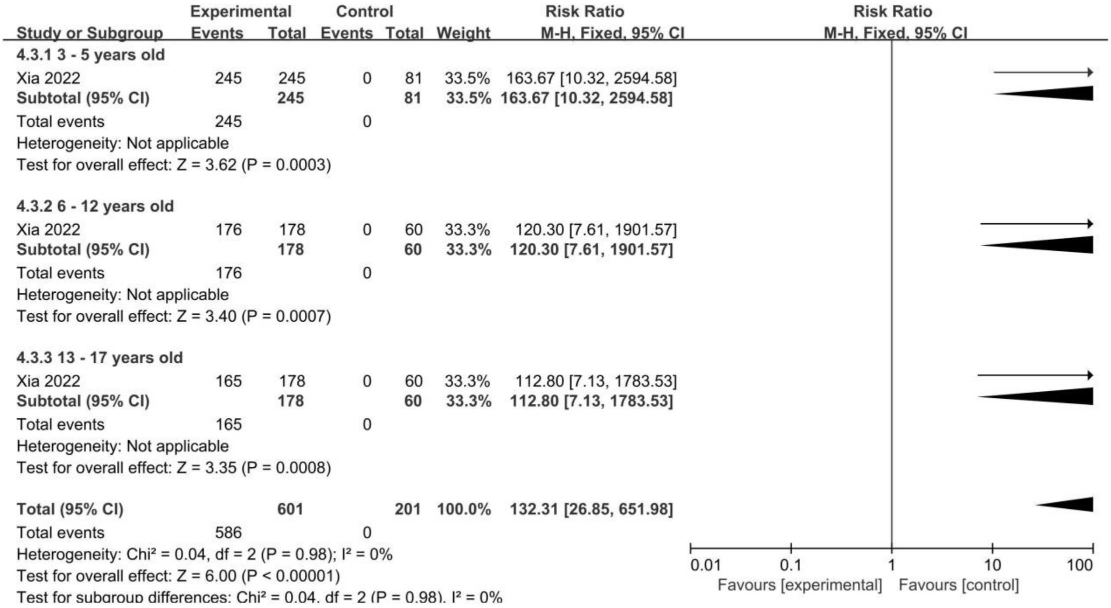

Figure S17. **COVID-19 diagnosed after vaccination in the vaccine group versus the control group: A) COVID-19 after the vaccination; B) COVID-19 after dose 2.**

A) COVID-19 after the vaccination

Study or Subgroup	Experimental Events	Experimental Total	Control Events	Control Total	Weight	Risk Ratio M-H, Random, 95% CI
**5.1.1 After dose 1 to before dose 2**						
Buddy Creech 2022	7	2687	18	880	16.8%	0.13 [0.05, 0.30]
Frenck 2021	3	1131	12	1129	14.2%	0.25 [0.07, 0.88]
**Subtotal (95% CI)**		**3818**		**2009**	**31.0%**	**0.16 [0.08, 0.32]**
Total events	10		30			
Heterogeneity: Tau² = 0.00; Chi² = 0.75, df = 1 (P = 0.39); I² = 0%						
Test for overall effect: Z = 5.05 (P < 0.00001)						
**5.1.2 Within 7 days after dose 2**						
Frenck 2021	0	1131	5	1129	6.2%	0.09 [0.01, 1.64]
**Subtotal (95% CI)**		**1131**		**1129**	**6.2%**	**0.09 [0.01, 1.64]**
Total events	0		5			
Heterogeneity: Not applicable						
Test for overall effect: Z = 1.63 (P = 0.10)						
**5.1.3 7 days after dose 2**						
Frenck 2021	0	1119	18	1110	6.5%	0.03 [0.00, 0.44]
Walter 2021	3	1305	16	663	14.4%	0.10 [0.03, 0.33]
**Subtotal (95% CI)**		**2424**		**1773**	**20.9%**	**0.08 [0.03, 0.24]**
Total events	3		34			
Heterogeneity: Tau² = 0.00; Chi² = 0.78, df = 1 (P = 0.38); I² = 0%						
Test for overall effect: Z = 4.45 (P < 0.00001)						
**5.1.4 14 days after dose 2**						
Ali 2021	1	2139	7	1042	9.3%	0.07 [0.01, 0.56]
Anderson 2022	170	4105	95	1371	19.9%	0.60 [0.47, 0.76]
Buddy Creech 2022	3	2644	4	853	12.7%	0.24 [0.05, 1.08]
**Subtotal (95% CI)**		**8888**		**3266**	**41.9%**	**0.30 [0.09, 0.97]**
Total events	174		106			
Heterogeneity: Tau² = 0.66; Chi² = 5.33, df = 2 (P = 0.07); I² = 62%						
Test for overall effect: Z = 2.01 (P = 0.04)						
**Total (95% CI)**		**16261**		**8177**	**100.0%**	**0.17 [0.07, 0.40]**
Total events	187		175			
Heterogeneity: Tau² = 0.97; Chi² = 30.60, df = 7 (P < 0.0001); I² = 77%						
Test for overall effect: Z = 4.05 (P < 0.0001)						
Test for subgroup differences: Chi² = 2.86, df = 3 (P = 0.41), I² = 0%						

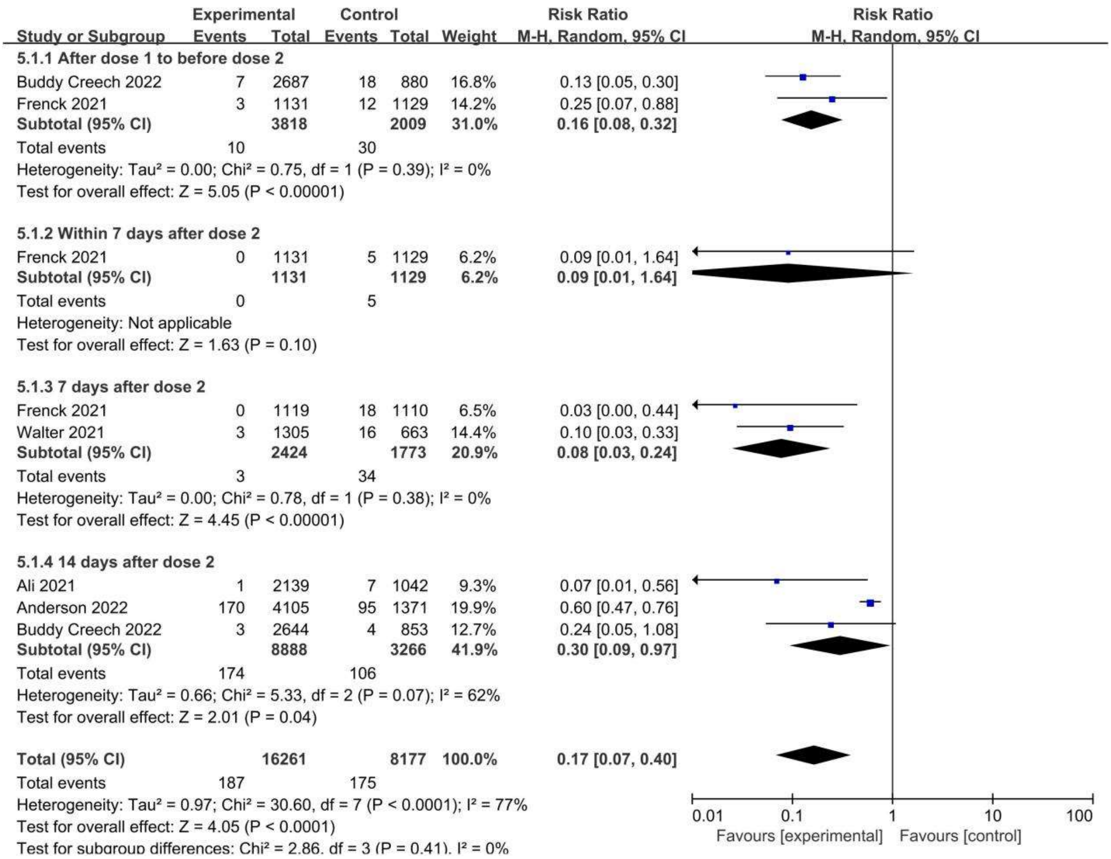

B) COVID-19 after dose 2

Study or Subgroup	Experimental Events	Experimental Total	Control Events	Control Total	Weight	Risk Ratio M-H, Random, 95% CI
**5.2.1 mRNA-1273 vaccine**						
Ali 2021	1	2139	7	1042	16.0%	0.07 [0.01, 0.56]
Anderson 2022	170	4105	95	1371	28.9%	0.60 [0.47, 0.76]
Buddy Creech 2022	3	2644	4	853	20.6%	0.24 [0.05, 1.08]
**Subtotal (95% CI)**		**8888**		**3266**	**65.5%**	**0.30 [0.09, 0.97]**
Total events	174		106			
Heterogeneity: Tau² = 0.66; Chi² = 5.33, df = 2 (P = 0.07); I² = 62%						
Test for overall effect: Z = 2.01 (P = 0.04)						
**5.2.2 BNT162b2 COVID-19 vaccine**						
Frenck 2021	0	1119	18	1110	11.8%	0.03 [0.00, 0.44]
Walter 2021	3	1305	16	663	22.7%	0.10 [0.03, 0.33]
**Subtotal (95% CI)**		**2424**		**1773**	**34.5%**	**0.08 [0.03, 0.24]**
Total events	3		34			
Heterogeneity: Tau² = 0.00; Chi² = 0.78, df = 1 (P = 0.38); I² = 0%						
Test for overall effect: Z = 4.45 (P < 0.00001)						
**Total (95% CI)**		**11312**		**5039**	**100.0%**	**0.16 [0.05, 0.56]**
Total events	177		140			
Heterogeneity: Tau² = 1.39; Chi² = 18.75, df = 4 (P = 0.0009); I² = 79%						
Test for overall effect: Z = 2.87 (P = 0.004)						
Test for subgroup differences: Chi² = 2.72, df = 1 (P = 0.10), I² = 63.3%						

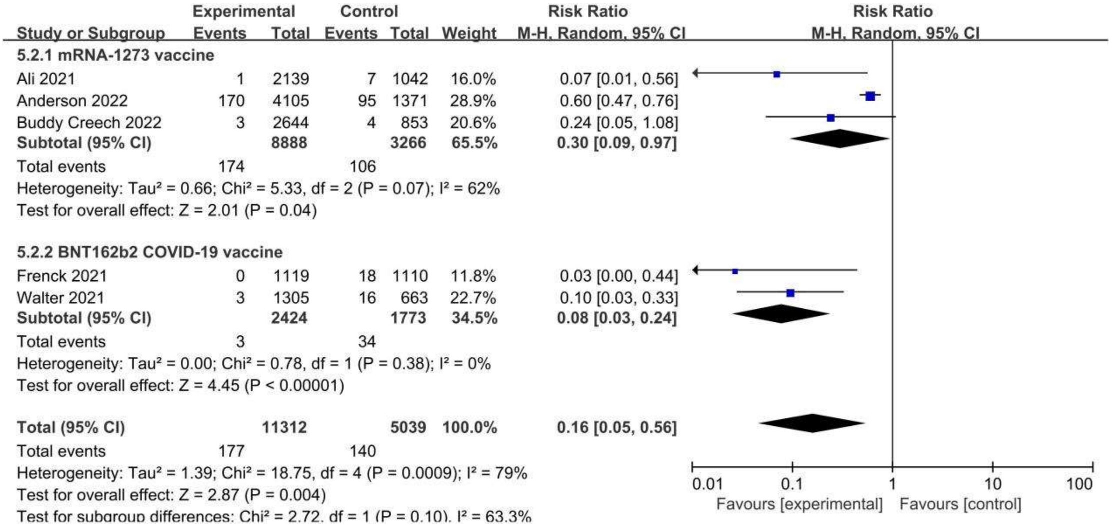

Table S1. Search formula in PubMed.

((((("Infant"[Mesh]) OR (Infants[Title/Abstract])) OR (("Child"[Mesh]) OR (Children[Title/Abstract]))) OR (("Adolescent"[Mesh]) OR ((((((((((((((((Adolescents[Title/Abstract]) OR (Adolescence[Title/Abstract])) OR (Teens[Title/Abstract])) OR (Teen[Title/Abstract])) OR (Teenagers[Title/Abstract])) OR (Teenager[Title/Abstract])) OR (Youth[Title/Abstract])) OR (Youths[Title/Abstract])) OR (Adolescents, Female[Title/Abstract])) OR (Adolescent, Female[Title/Abstract])) OR (Female Adolescent[Title/Abstract])) OR (Female Adolescents[Title/Abstract])) OR (Adolescents, Male[Title/Abstract])) OR (Adolescent, Male[Title/Abstract])) OR (Male Adolescent[Title/Abstract])) OR (Male Adolescents[Title/Abstract]))))) AND (((("COVID-19 Vaccines"[Mesh]) OR (((COVID 19 Vaccines[Title/Abstract]) OR (Vaccines, COVID-19[Title/Abstract])) OR (SARS Coronavirus 2 Vaccines[Title/Abstract])) OR (COVID19 Virus Vaccines[Title/Abstract])) OR (Vaccines, COVID19 Virus[Title/Abstract])) OR (Virus Vaccines, COVID19[Title/Abstract])) OR (COVID19 Virus Vaccine[Title/Abstract])) OR (Vaccine, COVID19 Virus[Title/Abstract])) OR (Virus Vaccine, COVID19[Title/Abstract])) OR (COVID19 Vaccines[Title/Abstract])) OR (Vaccines, COVID19[Title/Abstract])) OR (COVID19 Vaccine[Title/Abstract])) OR (Vaccine, COVID19[Title/Abstract])) OR (SARS-CoV-2 Vaccines[Title/Abstract])) OR (SARS CoV 2 Vaccines[Title/Abstract])) OR (Vaccines, SARS-CoV-2[Title/Abstract])) OR (SARS-CoV-2 Vaccine[Title/Abstract])) OR (SARS CoV 2 Vaccine[Title/Abstract])) OR (Vaccine, SARS-CoV-2[Title/Abstract])) OR (SARS2 Vaccines[Title/Abstract])) OR (Vaccines, SARS2[Title/Abstract])) OR (SARS2 Vaccine[Title/Abstract])) OR (Vaccine, SARS2[Title/Abstract])) OR (Coronavirus Disease 2019 Vaccines[Title/Abstract])) OR (Coronavirus Disease 2019 Vaccine[Title/Abstract])) OR (Coronavirus Disease 2019 Virus Vaccine[Title/Abstract])) OR (Coronavirus Disease 2019 Virus Vaccines[Title/Abstract])) OR (Coronavirus Disease-19 Vaccines[Title/Abstract])) OR (Coronavirus Disease 19 Vaccines[Title/Abstract])) OR (Vaccines, Coronavirus Disease-19[Title/Abstract])) OR (Coronavirus Disease-19 Vaccine[Title/Abstract])) OR (Coronavirus Disease 19 Vaccine[Title/Abstract])) OR (Vaccine, Coronavirus Disease-19[Title/Abstract])) OR (COVID 19 Vaccine[Title/Abstract])) OR (Vaccine, COVID 19[Title/Abstract])) OR (2019-nCoV Vaccine[Title/Abstract])) OR (2019 nCoV Vaccine[Title/Abstract])) OR (Vaccine, 2019-nCoV[Title/Abstract])) OR (2019 Novel Coronavirus Vaccines[Title/Abstract])) OR (2019 Novel Coronavirus Vaccine[Title/Abstract])) OR (2019-nCoV Vaccines[Title/Abstract])) OR (2019 nCoV Vaccines[Title/Abstract])) OR (Vaccines, 2019-nCoV[Title/Abstract])) OR (COVID-19 Vaccine[Title/Abstract])) OR (Vaccine, COVID-19[Title/Abstract])) OR (COVID-19 Virus Vaccines[Title/Abstract])) OR (COVID 19 Virus Vaccines[Title/Abstract])) OR (Vaccines, COVID-19 Virus[Title/Abstract])) OR (Virus Vaccines, COVID-19[Title/Abstract])) OR (COVID-19 Virus Vaccine[Title/Abstract])) OR (COVID 19 Virus Vaccine[Title/Abstract])) OR (Vaccine, COVID-19 Virus[Title/Abstract])) OR (Virus Vaccine, COVID-19[Title/Abstract]))) OR (("COVID-19"[Mesh]) OR ((((((((((((((((((((((((((((((((((((COVID 19[Title/Abstract]) OR (SARS-CoV-2 Infection[Title/Abstract])) OR (Infection, SARS-CoV-2[Title/Abstract])) OR (SARS CoV 2

Infection[Title/Abstract])) OR (SARS-CoV-2 Infections[Title/Abstract])) OR (2019 Novel Coronavirus Disease[Title/Abstract])) OR (2019 Novel Coronavirus Infection[Title/Abstract])) OR (2019-nCoV Disease[Title/Abstract])) OR (2019 nCoV Disease[Title/Abstract])) OR (2019-nCoV Diseases[Title/Abstract])) OR (Disease, 2019-nCoV[Title/Abstract])) OR (COVID-19 Virus Infection[Title/Abstract])) OR (COVID 19 Virus Infection[Title/Abstract])) OR (COVID-19 Virus Infections[Title/Abstract])) OR (Infection, COVID-19 Virus[Title/Abstract])) OR (Virus Infection, COVID-19[Title/Abstract])) OR (Coronavirus Disease 2019[Title/Abstract])) OR (Disease 2019, Coronavirus[Title/Abstract])) OR (Coronavirus Disease-19[Title/Abstract])) OR (Coronavirus Disease 19[Title/Abstract])) OR (Severe Acute Respiratory Syndrome Coronavirus 2 Infection[Title/Abstract])) OR (SARS Coronavirus 2 Infection[Title/Abstract])) OR (COVID-19 Virus Disease[Title/Abstract])) OR (COVID 19 Virus Disease[Title/Abstract])) OR (COVID-19 Virus Diseases[Title/Abstract])) OR (Disease, COVID-19 Virus[Title/Abstract])) OR (Virus Disease, COVID-19[Title/Abstract])) OR (2019-nCoV Infection[Title/Abstract])) OR (2019 nCoV Infection[Title/Abstract])) OR (2019-nCoV Infections[Title/Abstract])) OR (Infection, 2019-nCoV[Title/Abstract])) OR (COVID19[Title/Abstract])) OR (COVID-19 Pandemic[Title/Abstract])) OR (COVID 19 Pandemic[Title/Abstract])) OR (Pandemic, COVID-19[Title/Abstract])) OR (COVID-19 Pandemics[Title/Abstract])))) OR (("SARS-CoV-2"[Mesh]) OR (((((((((((((((((((((((SARS Coronavirus 2[Title/Abstract]) OR (Coronavirus 2, SARS[Title/Abstract])) OR (Coronavirus Disease 2019 Virus[Title/Abstract])) OR (2019 Novel Coronavirus[Title/Abstract])) OR (2019 Novel Coronaviruses[Title/Abstract])) OR (Coronavirus, 2019 Novel[Title/Abstract])) OR (Novel Coronavirus, 2019[Title/Abstract])) OR (Wuhan Seafood Market Pneumonia Virus[Title/Abstract])) OR (SARS-CoV-2 Virus[Title/Abstract])) OR (SARS CoV 2 Virus[Title/Abstract])) OR (SARS-CoV-2 Viruses[Title/Abstract])) OR (Virus, SARS-CoV-2[Title/Abstract])) OR (2019-nCoV[Title/Abstract])) OR (COVID-19 Virus[Title/Abstract])) OR (COVID 19 Virus[Title/Abstract])) OR (COVID-19 Viruses[Title/Abstract])) OR (Virus, COVID-19[Title/Abstract])) OR (Wuhan Coronavirus[Title/Abstract])) OR (Coronavirus, Wuhan[Title/Abstract])) OR (COVID19 Virus[Title/Abstract])) OR (COVID19 Viruses[Title/Abstract])) OR (Virus, COVID19[Title/Abstract])) OR (Viruses, COVID19[Title/Abstract])))))) AND (((randomized controlled trial[Publication Type] OR randomized[Title/Abstract] OR placebo[Title/Abstract])))

Table S2. Search formula in Embase.

#19. #10 AND #17 AND #18	582	21 Oct 2022
#18. 'randomized controlled trial':ab,ti OR 'randomized':ab,ti OR 'placebo':ab,ti	1,075,276	21 Oct 2022
#17. #11 OR #12 OR #13 OR #14 OR #15 OR #16	340,714	21 Oct 2022
#16. 'sars-cov-2':ab,ti OR 'coronavirus disease 2019 virus':ab,ti OR '2019 novel coronavirus':ab,ti OR	102,278	21 Oct 2022

	'2019 novel coronaviruses':ab,ti OR 'coronavirus, 2019 novel':ab,ti OR 'novel coronavirus, 2019':ab,ti OR 'wuhan seafood market pneumonia virus':ab,ti OR 'sars-cov-2 virus':ab,ti OR 'sars cov 2 virus':ab,ti OR 'sars-cov-2 viruses':ab,ti OR 'virus, sars-cov-2':ab,ti OR '2019-ncov':ab,ti OR 'covid-19 virus':ab,ti OR 'covid 19 virus':ab,ti OR 'covid-19 viruses':ab,ti OR 'virus, covid-19':ab,ti OR 'wuhan coronavirus':ab,ti OR 'coronavirus, wuhan':ab,ti OR 'sars coronavirus 2':ab,ti OR 'coronavirus 2, sars':ab,ti OR 'severe acute respiratory syndrome coronavirus 2':ab,ti		
#15.	'severe acute respiratory syndrome coronavirus 2'/exp	77,665	21 Oct 2022
#14.	'covid-19':ab,ti OR 'covid 19':ab,ti OR 'covid-19 virus disease':ab,ti OR 'covid 19 virus disease':ab,ti OR 'covid-19 virus diseases':ab,ti OR 'disease, covid-19 virus':ab,ti OR 'virus disease, covid-19':ab,ti OR 'covid-19 virus infection':ab,ti OR 'covid 19 virus infection':ab,ti OR 'covid-19 virus infections':ab,ti OR 'infection, covid-19 virus':ab,ti OR 'virus infection, covid-19':ab,ti OR '2019-ncov infection':ab,ti OR '2019 ncov infection':ab,ti OR '2019-ncov infections':ab,ti OR 'infection, 2019-ncov':ab,ti OR 'coronavirus disease-19':ab,ti OR 'coronavirus disease 19':ab,ti OR '2019 novel coronavirus disease':ab,ti OR '2019 novel coronavirus infection':ab,ti OR '2019-ncov disease':ab,ti OR '2019 ncov disease':ab,ti OR '2019-ncov diseases':ab,ti OR 'disease, 2019-ncov':ab,ti OR 'covid19':ab,ti OR 'coronavirus disease 2019':ab,ti OR 'disease 2019, coronavirus':ab,ti OR 'sars coronavirus 2 infection':ab,ti OR 'sars-cov-2 infection':ab,ti OR 'infection, sars-cov-2':ab,ti OR 'sars cov 2 infection':ab,ti OR 'sars-cov-2 infections':ab,ti OR	289,367	21 Oct 2022

'covid-19 pandemic':ab,ti OR 'covid 19 pandemic':ab,ti OR 'covid-19 pandemics':ab,ti OR 'pandemic, covid-19':ab,ti		
#13. 'coronavirus disease 2019'/exp	262,480	21 Oct 2022
#12. 'covid-19 vaccines':ab,ti OR 'covid 19 vaccines':ab,ti OR 'vaccines, covid-19':ab,ti OR 'covid-19 virus vaccines':ab,ti OR 'covid 19 virus vaccines':ab,ti OR 'vaccines, covid-19 virus':ab,ti OR 'virus vaccines, covid-19':ab,ti OR 'covid-19 virus vaccine':ab,ti OR 'covid 19 virus vaccine':ab,ti OR 'vaccine, covid-19 virus':ab,ti OR 'virus vaccine, covid-19':ab,ti OR 'covid19 virus vaccines':ab,ti OR 'vaccines, covid19 virus':ab,ti OR 'virus vaccines, covid19':ab,ti OR 'covid19 virus vaccine':ab,ti OR 'vaccine, covid19 virus':ab,ti OR 'virus vaccine, covid19':ab,ti OR 'covid19 vaccines':ab,ti OR 'vaccines, covid19':ab,ti OR 'covid19 vaccine':ab,ti OR 'vaccine, covid19':ab,ti OR 'sars-cov-2 vaccines':ab,ti OR 'sars cov 2 vaccines':ab,ti OR 'vaccines, sars-cov-2':ab,ti OR 'sars-cov-2 vaccine':ab,ti OR 'sars cov 2 vaccine':ab,ti OR 'vaccine, sars-cov-2':ab,ti OR 'sars2 vaccines':ab,ti OR 'vaccines, sars2':ab,ti OR 'sars2 vaccine':ab,ti OR 'vaccine, sars2':ab,ti OR 'coronavirus disease 2019 vaccines':ab,ti OR 'coronavirus disease 2019 vaccine':ab,ti OR 'coronavirus disease 2019 virus vaccine':ab,ti OR 'coronavirus disease 2019 virus vaccines':ab,ti OR 'coronavirus disease-19 vaccines':ab,ti OR 'coronavirus disease 19 vaccines':ab,ti OR 'vaccines, coronavirus disease-19':ab,ti OR 'coronavirus disease-19 vaccine':ab,ti OR 'coronavirus disease 19 vaccine':ab,ti OR 'vaccine, coronavirus disease-19':ab,ti OR 'covid 19 vaccine':ab,ti OR 'vaccine, covid 19':ab,ti OR '2019-ncov vaccine':ab,ti OR '2019 ncov vaccine':ab,ti OR 'vaccine, 2019-ncov':ab,ti OR '2019 novel coronavirus vaccines':ab,ti OR '2019 novel coronavirus vaccine':ab,ti OR '2019-ncov vaccines':ab,ti OR '2019 ncov vaccines':ab,ti OR 'vaccines, 2019-ncov':ab,ti OR 'covid-19 vaccine':ab,ti	15,561	21 Oct 2022

OR 'vaccine, covid-19':ab,ti OR 'sars coronavirus 2 vaccines':ab,ti		
#11. 'sars-cov-2 vaccine'/exp	26,007	21 Oct 2022
#10. #3 OR #6 OR #9	4,561,445	21 Oct 2022
#9. #7 OR #8	1,978,126	21 Oct 2022
#8. 'adolescents':ab,ti OR 'adolescence':ab,ti OR 'teens':ab,ti OR 'teen':ab,ti OR 'teenagers':ab,ti OR 'teenager':ab,ti OR 'youth':ab,ti OR 'youths':ab,ti OR 'adolescents, female':ab,ti OR 'adolescent, female':ab,ti OR 'female adolescent':ab,ti OR 'female adolescents':ab,ti OR 'adolescents, male':ab,ti OR 'adolescent, male':ab,ti OR 'male adolescent':ab,ti OR 'male adolescents':ab,ti	452,000	21 Oct 2022
#7. 'adolescent'/exp	1,855,848	21 Oct 2022
#6. #4 OR #5	3,578,055	21 Oct 2022
#5. 'children':ab,ti	1,599,278	21 Oct 2022
#4. 'child'/exp	3,242,540	21 Oct 2022
#3. #1 OR #2	1,346,803	21 Oct 2022
#2. 'infants':ab,ti	365,023	21 Oct 2022
#1. 'infant'/exp	1,249,634	21 Oct 2022

Table S3. Search formula in the Cochrane library.

#1.MeSH descriptor: [Infant] explode all trees
#2.(Infants):ti,ab,kw
#3. #1 OR #2
#4. MeSH descriptor: [Child] explode all trees
#5. (Children):ti,ab,kw
#6. #4 OR #5
#7. MeSH descriptor: [Adolescent] explode all trees
#8. (Adolescents):ti,ab,kw OR (Adolescence):ti,ab,kw OR (Teens):ti,ab,kw OR (Teen):ti,ab,kw OR (Teenagers):ti,ab,kw OR (Teenager):ti,ab,kw OR (Youth):ti,ab,kw OR (Youths):ti,ab,kw OR (Adolescents, Female):ti,ab,kw OR (Adolescent, Female):ti,ab,kw OR (Female Adolescent):ti,ab,kw OR (Female Adolescents):ti,ab,kw OR (Adolescents, Male):ti,ab,kw OR (Adolescent, Male):ti,ab,kw OR (Male Adolescent):ti,ab,kw OR (Male Adolescents):ti,ab,kw
#9. #7 OR #8
#10. #3 OR #6 OR #9
#11. MeSH descriptor: [COVID-19 Vaccines] explode all trees
#12. (COVID 19 Vaccines):ti,ab,kw OR (Vaccines, COVID-19):ti,ab,kw OR (SARS Coronavirus 2 Vaccines):ti,ab,kw OR (COVID19 Virus Vaccines):ti,ab,kw OR (Vaccines, COVID19

Virus):ti,ab,kw OR (Virus Vaccines, COVID19):ti,ab,kw OR (COVID19 Virus Vaccine):ti,ab,kw OR (Vaccine, COVID19 Virus):ti,ab,kw OR (Virus Vaccine, COVID19):ti,ab,kw OR (COVID19 Vaccines):ti,ab,kw OR (Vaccines, COVID19):ti,ab,kw OR (COVID19 Vaccine):ti,ab,kw OR (Vaccine, COVID19):ti,ab,kw OR (SARS-CoV-2 Vaccines):ti,ab,kw OR (SARS CoV 2 Vaccines):ti,ab,kw OR (Vaccines, SARS-CoV-2):ti,ab,kw OR (SARS-CoV-2 Vaccine):ti,ab,kw OR (SARS CoV 2 Vaccine):ti,ab,kw OR (Vaccine, SARS-CoV-2):ti,ab,kw OR (SARS2 Vaccines):ti,ab,kw OR (Vaccines, SARS2):ti,ab,kw OR (SARS2 Vaccine):ti,ab,kw OR (Vaccine, SARS2):ti,ab,kw OR (Coronavirus Disease 2019 Vaccines):ti,ab,kw OR (Coronavirus Disease 2019 Vaccine):ti,ab,kw OR (Coronavirus Disease 2019 Virus Vaccine):ti,ab,kw OR (Coronavirus Disease 2019 Virus Vaccines):ti,ab,kw OR (Coronavirus Disease-19 Vaccines):ti,ab,kw OR (Coronavirus Disease 19 Vaccines):ti,ab,kw OR (Vaccines, Coronavirus Disease-19):ti,ab,kw OR (Coronavirus Disease-19 Vaccine):ti,ab,kw OR (Coronavirus Disease 19 Vaccine):ti,ab,kw OR (Vaccine, Coronavirus Disease-19):ti,ab,kw OR (COVID 19 Vaccine):ti,ab,kw OR (Vaccine, COVID 19):ti,ab,kw OR (2019 nCoV Vaccine):ti,ab,kw OR (2019 Novel Coronavirus Vaccines):ti,ab,kw OR (2019 Novel Coronavirus Vaccine):ti,ab,kw OR (2019 nCoV Vaccines):ti,ab,kw OR (COVID-19 Vaccine):ti,ab,kw OR (Vaccine, COVID-19):ti,ab,kw OR (COVID-19 Virus Vaccines):ti,ab,kw OR (COVID 19 Virus Vaccines):ti,ab,kw OR (Vaccines, COVID-19 Virus):ti,ab,kw OR (Virus Vaccines, COVID-19):ti,ab,kw OR (COVID-19 Virus Vaccine):ti,ab,kw OR (COVID 19 Virus Vaccine):ti,ab,kw OR (Vaccine, COVID-19 Virus):ti,ab,kw OR (Virus Vaccine, COVID-19):ti,ab,kw

#13. #11 OR #12

#14. MeSH descriptor: [COVID-19] explode all trees

#15. (COVID 19):ti,ab,kw OR (SARS-CoV-2 Infection):ti,ab,kw OR (Infection, SARS-CoV-2):ti,ab,kw OR (SARS CoV 2 Infection):ti,ab,kw OR (SARS-CoV-2 Infections):ti,ab,kw OR (2019 Novel Coronavirus Disease):ti,ab,kw OR (2019 Novel Coronavirus Infection):ti,ab,kw OR (2019 nCoV Disease):ti,ab,kw OR (COVID-19 Virus Infection):ti,ab,kw OR (COVID-19 Virus Infections):ti,ab,kw OR (Infection, COVID-19 Virus):ti,ab,kw OR (Virus Infection, COVID-19):ti,ab,kw OR (Coronavirus Disease 2019):ti,ab,kw OR (Disease 2019, Coronavirus):ti,ab,kw OR (Coronavirus Disease-19):ti,ab,kw OR (Coronavirus Disease 19):ti,ab,kw OR (Severe Acute Respiratory Syndrome Coronavirus 2 Infection):ti,ab,kw OR (SARS Coronavirus 2 Infection):ti,ab,kw OR (COVID-19 Virus Disease):ti,ab,kw OR (COVID 19 Virus Disease):ti,ab,kw OR (COVID-19 Virus Diseases):ti,ab,kw OR (Disease, COVID-19 Virus):ti,ab,kw OR (Virus Disease, COVID-19):ti,ab,kw OR (2019 nCoV Infection):ti,ab,kw OR (COVID19):ti,ab,kw OR (COVID-19 Pandemic):ti,ab,kw OR (COVID 19 Pandemic):ti,ab,kw OR (Pandemic, COVID-19):ti,ab,kw OR (COVID-19 Pandemics):ti,ab,kw

#16. #14 OR #15

#17. MeSH descriptor: [SARS-CoV-2] explode all trees

#18. (SARS Coronavirus 2):ti,ab,kw OR (Coronavirus 2, SARS):ti,ab,kw OR (Coronavirus Disease 2019 Virus):ti,ab,kw OR (2019 Novel Coronavirus):ti,ab,kw OR (2019 Novel Coronaviruses):ti,ab,kw OR (Coronavirus, 2019 Novel):ti,ab,kw OR (Novel Coronavirus, 2019):ti,ab,kw OR (Wuhan Seafood Market Pneumonia Virus):ti,ab,kw OR (SARS-CoV-2 Virus):ti,ab,kw OR (SARS CoV 2 Virus):ti,ab,kw OR (SARS-CoV-2 Viruses):ti,ab,kw OR (Virus, SARS-CoV-2):ti,ab,kw OR (COVID-19 Virus):ti,ab,kw OR (COVID 19 Virus):ti,ab,kw OR (COVID-19 Viruses):ti,ab,kw OR (Virus, COVID-19):ti,ab,kw OR (Wuhan Coronavirus):ti,ab,kw OR (Coronavirus, Wuhan):ti,ab,kw OR (COVID19 Virus):ti,ab,kw OR (COVID19 Viruses):ti,ab,kw OR

(Virus, COVID19):ti,ab,kw OR (Viruses, COVID19):ti,ab,kw

#19. #17 OR #18

#20. #13 OR #16 OR #19

#21. #10 AND #20

Table S4. Specific adverse reactions in the inactivated vaccine group versus the control group after dose 1 and dose 2.

		After dose 1			After dose 2		
	No. of studies	RR (95% CI)	I^2	p-value	RR (95% CI)	I^2	p-value
**Overall**	2	1.76 [1.20, 2.57]	38	<0.05	2.18 [1.30, 3.67]	0	<0.05
**Local pain**	2	21.53 [3.00, 154.35]	0	<0.05	6.84 [1.96, 23.90]	0	<0.05
**Erythema or Redness**	1 (after dose 1）	1.34 [0.15, 11.89]	Not applicable	>0.05	1.21 [0.30, 4.83]	0	>0.05
	2 (after dose 2)						
**Swelling or Hardness**	2	0.50 [0.10, 2.42]	22	>0.05	2.24 [0.28, 18.17]	0	>0.05
**Fever**	2	1.54 [0.84, 2.82]	0	>0.05	1.64 [0.64, 4.24]	0	>0.05
**Headache**	2	1.04 [0.33, 3.32]	48	>0.05	1.21 [0.20, 7.38]	16	>0.05
**Fatigue**	2	1.11 [0.19, 6.54]	0	>0.05	0.79 [0.03, 19.23]	Not applicable	>0.05
**Myalgia**	2	2.09 [0.26, 16.84]	0	>0.05	0.89 [0.09, 8.49]	0	>0.05
**Nausea or Vomiting**	1	0.52 [0.10, 2.82]	Not applicable	>0.05	0.78 [0.08, 7.46]	Not applicable	>0.05
**Diarrhea**	2 (after dose 1）	0.46 [0.17, 1.27]	0	>0.05	0.26 [0.02, 4.14]	Not applicable	>0.05
	1 (after dose 2)						

[1] RR, Risk ratio ; CI, confidence interval; $p<0.05$.

Table S5. Specific adverse reactions in the subunit vaccine group versus the control group after dose 1 and dose 2.

		After dose 1			After dose 2		
	No. of studies	RR (95% CI)	I^2	p-value	RR (95% CI)	I^2	p-value
**Overall**	1	1.66 [1.26, 2.17]	19	<0.05	1.40 [1.02, 1.92]	12	<0.05
**Local pain**	1	2.91 [1.74, 4.84]	Not applicable	<0.05	1.97 [1.23, 3.16]	Not applicable	<0.05
**Erythema or Redness**	1	0.86 [0.04, 17.74]	Not applicable	>0.05	1.21 [0.30, 4.83]	Not applicable	>0.05
**Swelling or Hardness**	1	2.59 [0.15, 44.71]	Not applicable	>0.05	2.24 [0.28, 18.17]	Not applicable	>0.05
**Fever**	1	1.21 [0.06, 23.08]	Not applicable	>0.05	1.64 [0.64, 4.24]	Not applicable	>0.05
**Headache**	1	0.93 [0.48, 1.78]	Not applicable	>0.05	1.21 [0.20, 7.38]	16	>0.05
**Fatigue**	1	1.47 [0.78, 2.77]	Not applicable	>0.05	0.79 [0.03, 19.23]	Not applicable	>0.05
**Myalgia**	1	1.12 [0.61, 2.05]	Not applicable	>0.05	0.89 [0.09, 8.49]	Not applicable	>0.05
**Nausea or Vomiting**	1	1.45 [0.34, 6.09]	Not applicable	>0.05	0.78 [0.08, 7.46]	Not applicable	>0.05
**Diarrhea**	1	1.45 [0.53, 3.92]	Not applicable	>0.05	0.26 [0.02, 4.14]	Not applicable	>0.05

[1] RR, Risk ratio ; CI, confidence interval; $p<0.05$.

Table S6. Specific adverse reactions in the adenovirus vector vaccine group versus the control group after dose 1 and dose 2.

	After dose 1				After dose 2		
	No. of studies	RR (95% CI)	I^2	p-value	RR (95% CI)	I^2	p-value
**Overall**	1	5.27 [2.80, 9.91]	0	<0.05	6.21 [2.40, 16.11]	0	<0.05
**Local pain**	1	5.67 [1.83, 17.55]	Not applicable	<0.05	14.64 [0.89, 240.57]	Not applicable	>0.05
**Erythema or Redness**	1	2.52 [0.12, 51.61]	Not applicable	>0.05	Not estimable	Not applicable	Not applicable
**Swelling or Hardness**	1	4.54 [0.25, 82.78]	Not applicable	>0.05	4.54 [0.25, 82.78]	Not applicable	>0.05
**Fever**	1	7.00 [1.74, 28.21]	Not applicable	<0.05	6.50 [0.87, 48.29]	Not applicable	>0.05
**Headache**	1	14.64 [0.89, 240.57]	Not applicable	>0.05	8.58 [0.51, 145.79]	16	>0.05
**Fatigue**	1	2.25 [0.51, 10.02]	Not applicable	>0.05	6.56 [0.38, 114.24]	Not applicable	>0.05
**Myalgia**	1	4.54 [0.25, 82.78]	Not applicable	>0.05	1.51 [0.06, 36.53]	Not applicable	>0.05
**Arthralgia**	1	1.51 [0.06, 36.53]	Not applicable	>0.05	1.51 [0.06, 36.53]	Not applicable	>0.05
**Nausea or Vomiting**	1	6.56 [0.38, 114.24]	Not applicable	>0.05	5.55 [0.31, 98.50]	Not applicable	>0.05

[1] RR, Risk ratio; CI, confidence interval; $p<0.05$.

Table S7. Specific adverse reactions in inactivated vaccine recipients of different ages after whole vaccinations.

	≥ 12 years				<12 years			
	No. of studies	RR (95% CI)	I^2	p-value	No. of studies	RR (95% CI)	I^2	p-value
**Local pain**	2	1.56 [0.88, 2.77]	92	>0.05	2	1.02 [0.57, 1.84]	84	>0.05
**Erythema or Redness**	2	0.29 [0.04, 2.18]	0	>0.05	2	0.57 [0.17, 1.91]	Not applicable	>0.05
**Swelling or Hardness**	2	0.72 [0.16, 3.15]	45	>0.05	2	1.27 [0.22, 7.55]	0	>0.05
**Fever**	2	1.10 [0.40, 3.06]	0	>0.05	2	1.04 [0.54, 1.99]	0	>0.05
**Headache**	2	1.04 [0.26, 4.07]	0	>0.05	2	0.28 [0.05, 1.69]	0	>0.05
**Nausea or Vomiting**	2	1.33 [0.06, 27.13]	Not applicable	>0.05	2	0.68 [0.10, 4.51]	0	>0.05
**Diarrhea**	2	0.43 [0.07, 2.57]	0	>0.05	2	0.21 [0.05, 0.93]	0	<0.05

[1] RR, Risk ratio; CI, confidence interval; $p<0.05$.

Table S8. Specific adverse reactions in subunit vaccine recipients of different ages after whole vaccinations.

	≥ 12 years				<12 years			
	No. of studies	RR (95% CI)	I^2	p-value	No. of studies	RR (95% CI)	I^2	p-value
**Local pain**	3	2.31 [1.99, 2.67]	90	<0.05	1	0.59 [0.34, 1.01]	Not applicable	=0.05
**Erythema or Redness**	3	1.19 [0.55, 2.58]	0	>0.05	1	0.44 [0.16, 1.24]	Not applicable	>0.05
**Swelling or Hardness**	3	3.11 [1.16, 8.30]	0	<0.05	1	0.33 [0.09, 1.30]	Not applicable	>0.05
**Headache**	3	1.17 [1.01, 1.35]	46	<0.05	1	1.33 [0.29, 6.15]	Not applicable	>0.05
**Nausea or Vomiting**	3	1.00 [0.75, 1.33]	0	>0.05	1	0.33 [0.02, 5.27]	Not applicable	>0.05
**Fatigue**	3	1.51 [1.27, 1.81]	0	<0.05	1	2.35 [0.12, 45.06]	Not applicable	>0.05
**Myalgia**	3	2.03 [1.71, 2.41]	70	<0.05	1	1.01 [0.04, 24.51]	Not applicable	>0.05

[1] RR, Risk ratio ; CI, confidence interval; $p<0.05$.
